# Tissue enrichment analysis for C. elegans genomics

**DOI:** 10.1186/s12859-016-1229-9

**Published:** 2016-09-13

**Authors:** David Angeles-Albores, Raymond Y. N. Lee, Juancarlos Chan, Paul W. Sternberg

**Affiliations:** HHMI and California Institute of Technology, Division of Biology and Biological Engineering, 1200 E California Blvd, Pasadena, 91125 USA

**Keywords:** Gene ontology, Anatomy ontology, WormBase, RNA-seq, High-throughput biology

## Abstract

**Background:**

Over the last ten years, there has been explosive development in methods for measuring gene expression. These methods can identify thousands of genes altered between conditions, but understanding these datasets and forming hypotheses based on them remains challenging. One way to analyze these datasets is to associate ontologies (hierarchical, descriptive vocabularies with controlled relations between terms) with genes and to look for enrichment of specific terms. Although Gene Ontology (GO) is available for *Caenorhabditis elegans*, it does not include anatomical information.

**Results:**

We have developed a tool for identifying enrichment of *C. elegans* tissues among gene sets and generated a website GUI where users can access this tool. Since a common drawback to ontology enrichment analyses is its verbosity, we developed a very simple filtering algorithm to reduce the ontology size by an order of magnitude. We adjusted these filters and validated our tool using a set of 30 gold standards from Expression Cluster data in WormBase. We show our tool can even discriminate between embryonic and larval tissues and can even identify tissues down to the single-cell level. We used our tool to identify multiple neuronal tissues that are down-regulated due to pathogen infection in *C. elegans*.

**Conclusions:**

Our Tissue Enrichment Analysis (TEA) can be found within WormBase, and can be downloaded using Python’s standard pip installer. It tests a slimmed-down *C. elegans* tissue ontology for enrichment of specific terms and provides users with a text and graphic representation of the results.

**Electronic supplementary material:**

The online version of this article (doi:10.1186/s12859-016-1229-9) contains supplementary material, which is available to authorized users.

## Background

RNA-seq and other high-throughput methods in biology have the ability to identify thousands of genes that are altered between conditions. These genes are often correlated in their biological characteristics or functions, but identifying these functions remains challenging. To interpret these long lists of genes, biologists need to abstract genes into concepts that are biologically relevant to form hypotheses about what is happening in the system. One such abstraction method relies on Gene Ontology (GO). GO provides a controlled set of hierarchically ordered terms [1, 2] that provide detailed descriptions about the molecular, cellular or biochemical functions of any gene. For a given gene list, existing software programs can query whether a particular term is enriched [3–6]. One area of biological significance that GO does not include is anatomy. One way to address this shortcoming is to use a ‘tissue ontology’ that provides a complete anatomical description for an organism (e.g.‘tissue’, ‘organ’ or ‘specific cell’), in this case for *C. elegans*. Such an ontology has been described previously for this organism [7]. Cells and tissues are physiologically relevant units with broad, relatively well-understood functionalities amenable to hypothesis formation. The *C. elegans* database, WormBase [8], maintains a curated list of gene expression data from the literature. Here we provide a new framework that analyzes a user-input list for enrichment of specific cells and tissues.

Another problem frequently associated with GO enrichment analysis is that it is often difficult to interpret due to the large number of terms associated with a given gene (which we refer to as ‘result verbosity’). DAVID, a common tool for GO enrichment analysis, clusters enriched terms into broad categories [9], whereas PANTHER [3, 10] attempts to solve this issue by employing a manually reduced ontology, GOslim (pers. comm., H. Yu and P. Thomas). To reduce verbosity, we have filtered our ontology using a small set of well-defined criteria to remove terms that do not contribute additional information. To our knowledge, such filtering has not been performed in an algorithmic fashion for a biological ontology before; indeed, DAVID does not employ term trimming a priori of testing, but rather fuzzy clustering *post* testing to reduce the number of ontology terms. Other pruning methods do exist (see for example [11, 12]), but the pruning is query-dependent or generates a brand new ‘brief ontology’ which satisfies a set of logic relationships and has certain connectivity requirements. We do not propose to regenerate a new ‘brief ontology’, but instead we use our approach to select those nodes that have sufficient annotated evidence for statistical testing. We believe our trimming methodology strikes a good balance between detailed tissue calling and conservative testing.

We have developed a tool that tests a user-provided list of genes for term enrichment using a nematode-specific tissue ontology. This ontology, which is not a module of Gene Ontology, is verbose. We select nodes from the ontology for statistical testing using an algorithmic approach, outlined below, that reduces multiple hypothesis testing issues by limiting testing to terms that are well-annotated. The results are provided to the user in a GUI that includes a table of results and an automatically generated bar-chart. This software addresses a previously unmet need in the *C. elegans* community for a tool that reliably and specifically links gene expression with changes in specific cells, organs or tissues in the worm.

## Results

### Generating a gene-tissue dictionary by specific node selection

#### Reducing term redundancy through a similarity metric

**Fig. 1:**
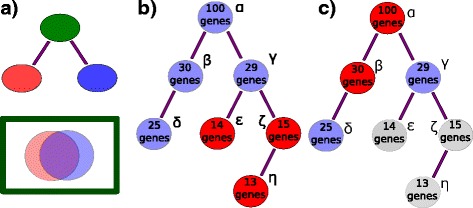
Schematic representation of trimming filters for an acyclical ontology. **a** The parent node (*green*) contains at least as many annotations as the union of the two sisters. These two sisters share annotations extensively, as expressed by the overlap in the Venn diagram, so they qualify for removal. **b** Nodes with less than a threshold number of genes are trimmed (*red*) and discarded from the dictionary. Here, the example threshold is 25 genes. Nodes *ε*,*ζ*,*η*, shown in red are removed. **c** Parent nodes are removed recursively, starting from the root, if all their daughter nodes have more than the threshold number of annotations. Nodes in *grey* (*ε*,*ζ*,*η*) were removed in the previous step. Nodes *α*,*β* shown in *red* are trimmed because each one has a complete daughter set. Only nodes *γ* and *δ* will be used to generate the static dictionary

For our tool, we employ a previously generated cell and tissue ontology for *C. elegans* [7], which is maintained and curated by WormBase. This ontology contains thousands of anatomiy terms, but not every term is equally well-annotated. As a first step to generate our tissue enrichment software, we wished to select tissue terms that were reasonably well-annotated, yet specific enough to provide insight and not redundant with other terms. For example, nematodes have a number of neurons that are placed symmetrically along the left/right body axis, and are functionally similar. These left/right neuronal pairs (which are sisters in the ontology) have almost identical annotations, with at most one or two gene differences between them, and therefore we cannot have statistical confidence in differentiating between them. As a result, testing these sister terms provides no additional information compared with testing only the parent node to these sisters. To identify redundancy, we defined two possible similarity metrics (see “Methods” section and Fig. 1a) that can be used to identify ontology sisters that have very high similarity between them. Intuitively, a set of sisters can be considered very similar if they share most gene annotations. Within a given set of sisters, we can calculate a similarity score for a single node by counting the number of unique annotations it contains and dividing by the total number of unique annotations in the sister set. Having assigned to each sister a similarity score, we can identify the **average** similarity score for this set of sisters, and if this average value exceeds a threshold, these sisters are not considered testable candidates. An alternative method is check whether **any** of the scores exceeds a predetermined threshold, and if so remove this sister set from the ontology. We referred to these two scoring criteria as ‘**avg**’ and ‘**any**’ respectively.

#### Terminal branch terms and parent terms can be safely removed in an algorithmic fashion

Another problem arises from the ontology being scarcely populated. Many nodes have 0–10 annotations, which we consider too few to accurately test. To solve this issue, we implemented another straightforward node selection strategy. For a given terminal node, we test whether the node has more than a threshold number of annotations. If it does not, the node is not used for statistical testing. The next higher node in the branch is tested and removed recursively until a node that satisfies the condition is found. At that point, no more nodes can be removed from that branch. This completion is guaranteed by the structure of the ontology: parent nodes inherit all of the annotations of all of their descendants, so the number of annotated terms monotonically increases with increasing term hierarchy (see Fig. 1b). In this way, we ensure that our term dictionary includes only those tissues that are considered sufficiently well annotated for statistical purposes.

Additionally, we reasoned that for any parent node if all its daughters were selected for testing, there was no additional benefit to test the parent. We removed parent nodes from the analysis if all their daughter nodes passed the annotation threshold (see Fig. 1c). We called this a ceiling filter. Applying these three filters reduced the number of ontology terms by an order of magnitude.

#### Filtering greatly reduces the number of nodes used for analysis

By itself, each of these filters can reduce the number of nodes employed for analysis, but applying the filters in different orders removes different numbers of nodes (not all the filters are commutative). We chose to always execute annotation and similarity thresholding first, followed by the ceiling filter. For validation (see below) we made a number of different dictionaries. The original ontology has almost 6,000 terms of which 1675 have at least 5 gene annotations. After filtering, dictionary sizes ranged from 21 to a maximum of 460 terms, which shows the number of terms in a scarcely annotated ontology can be reduced by an order of magnitude through the application of a few simple filters (see Table 1). These filters were used to compile a static dictionary that we employ for all analyses (see “Validation of the algorithm and optimizing parameter selection” section for details). Our trimming pipeline is applied as part of each new WormBase release. This ensures that the ontology database we are using remains up-to-date with regards to both addition or removal of specific terms as well as with regard to gene expression annotations.

**Table 1:** Parameter specifications and number of tissues for all dictionaries

Annotation cutoff	Similarity threshold	Method	No. of terms in dictionary
25	0.9	any	460
25	0.9	avg	461
25	0.95	any	466
25	0.95	avg	468
25	1	any	476
25	1	avg	476
33	0.9	any	261
33	0.9	avg	255
33	0.95	any	261
33	0.95	avg	262
33	1	any	247
33	1	avg	247
50	0.9	any	83
50	0.9	avg	77
50	0.95	any	82
50	0.95	avg	81
50	1	any	70
50	1	avg	70
100	0.9	any	45
100	0.9	avg	35
100	0.95	any	42
100	0.95	avg	36
100	1	any	21
100	1	avg	21

The ‘Method’ column refers to the trimming criterion for the similarity metric. We used two such criteria, ‘any’ and ‘avg’.‘any’: For a given sister set, if any sister had a similarity exceeding the corresponding threshold, all sisters were removed from the final dictionary. ‘avg’: For a given sister set, if the average similarity across all the sisters in the set was greater than the corresponding threshold, all sisters were removed from the final dictionary

### Tissue enrichment testing via a hypergeometric model

Having built a static dictionary, we generated a Python script that implements a significance testing algorithm based on the hypergeometric model. Briefly, the hypergeometric model tests the probability of observing *n*_*i*_ occurences of a tissue *i* in a list of size *M* if there are *m*_*i*_ labels for that tissue in a dictionary of total size *N* that are drawn without replacement. Mathematically, this is expressed as:

1 $$\begin{array}{@{}rcl@{}} \mathrm{P}(n_{i} | N, m_{i}, M) = \frac{{m_{i} \choose n_{i}} {M-m_{i} \choose N - n_{i}}}{{N \choose n_{i}}}{.} \end{array} $$

Although a user will input gene IDs, we test the number of ocurrences of a term within the gene list, so a single gene can contribute to multiple terms. Due to the discrete nature of the hypergeometric distribution, this algorithm can generate artifacts when the list is small. To avoid spurious results, a tissue is never considered significant if there are no annotations for it in the user-provided list.

Once the *p*-values for each term have been calculated, we apply a standard FDR correction using a Benjamini-Hochberg step-up algorithm [13]. FDR corrected *p*-values are called *q*-values. Genes that have a *q*-value less than a given alpha are considered significant. Our default setting is an alpha of 0.1, which is a standard threshold broadly agreed upon by the scientific community (see for example [14–16]). This threshold cannot be altered in the web GUI, but is user tunable through our command-line implementation.

**Fig. 2:**
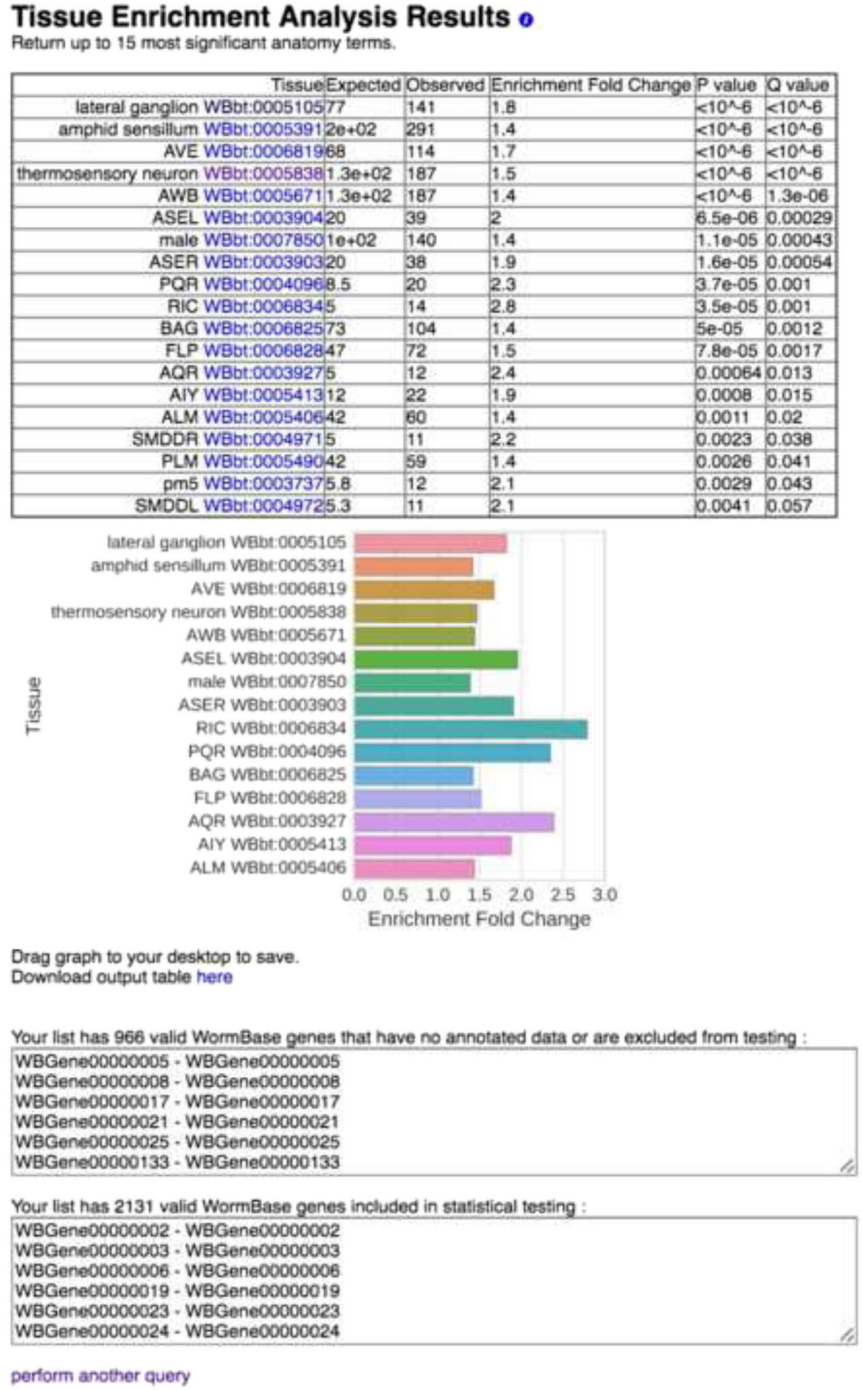
Screenshot of results from the web GUI. After inputting a gene-list, the user is provided with the results. An HTML table is output with hyperlinks to the ontology terms. A publication-ready graph is provided below, which can be saved by dragging to the desktop. The graph is colored for better visualization; color is not intended to convey information. The graph and the table show anatomy terms in human-readable format, followed by their unique WBbt ID. Finally, lists of the genes used and discarded for the analysis are also presented

**Fig. 3:**
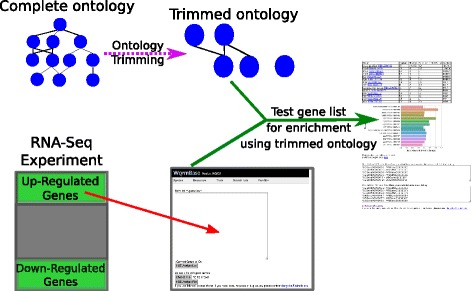
TEA Workflow. The complete ontology is annotated continuously by WormBase curators. After each update, the ontology is processed to remove uninformative terms, and the remaining terms are used for statistical testing. Users can select a gene list and input it into our tool using our WormBase portal. The gene list is tested for enrichment using the trimmed ontology, and results are output in tabular and graphic formats for analysis

Users input a gene list using any valid gene name for *C. elegans*. These names are processed into standard WormBase gene IDs (WBGene IDs). The program returns a table containing all the enriched terms and associated information such as number of terms in gene list and expected number of terms. Finally, the program can also return a bar chart of the enrichment fold change for the fifteen tissues with the lowest measured *q*-values. The bars in the graph are sorted in ascending order of *q*-value and then in descending order of fold-change. Bars are colored for ease of viewing, and color does not convey information. Our software is implemented in an easy to use GUI (see Fig. 2; alternatively, users can interface with TEA via python, see Additional file 1). Anatomy terms are displayed in human-readable format followed by their unique ontology ID (WBbt ID). In summary, each time the ontology annotations are updated, a new trimmed ontology is generated using our filters; in parallel, users can submit their gene lists through WormBase for testing, with results output in a number of formats (see Fig. 3).

### Validation of the algorithm and optimizing parameter selection

We wanted to select a dictionary that included enough terms to be specific beyond the most basic *C. elegans* tissues, yet would minimize the number of spurious results and which had a good dynamic range in terms of enrichment fold-change. Larger tissues are correlated with better annotation, so increasing term specificity is associated with losses in statistical power. To help us select an appropriate dictionary and validate our tool, we used a set of 30 gold standards based on microarray and RNA-seq literature which are believed to be enriched in specific tissues [17–24]. These data sets are annotated gene lists derived from the corresponding Expression Cluster data in WormBase. Some of these studies have been used to annotate gene expression, and so they did not constitute an independent testing set. To correct this flaw, we built a clean dictionary that specifically excluded all annotation evidence that came from these studies.

**Fig. 4:**
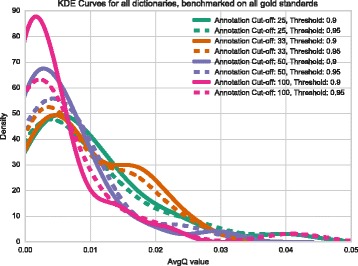
Kernel density estimates (KDE) for 30 gold standard datasets. We ran TEA on 30 datasets we believed to be enriched in particular tissues and pooled all the results to observe the distribution of *q*-values. The mode of the distribution for dictionaries with annotation cut-offs of 100 and 50 genes are very similar; however, when the cut-off is lowered to 25 genes, the mode of the distribution shifts to the left, potentially signalling a decrease in measurement power

As a first attempt to select a dictionary, we generated all possible combinations of dictionaries with minimal annotations of 10, 25, 33, 50 and 100 genes and similarity cutoffs of 0.9, 0.95 and 1, using ‘avg’ or ‘any’ similarity thresholding methods (see Table 1). The number of remaining ontology terms was inversely correlated to the minimum annotation cutoff, and was largely insensitive to the similarity threshold in the range we explored. Next, we analyzed all 30 datasets using each dictionary. Because of the large number of results, instead of analyzing each set of terms individually, we measured the average *q*-value for significantly enriched terms in each dataset without regard for the perceived accuracy of the terms that tested significant. We found that the similarity threshold mattered relatively little for any dictionary. We also noticed that the ‘any’ thresholding method resulted in tighter histograms with a mode closer to 0. For this reason, we chose the ‘any’ method for dictionary generation. The average *q*-value increased with decreasing annotation cut-off (see Fig. 4), which reflects the decreasing statistical power associated with fewer annotations per term, but we remained agnostic as to how significant is the trade-off between power and term specificity. Based on these observations, we ruled out the dictionary with the 100 gene annotation cut-off: it had the fewest terms and its *q*-values were not low enough in our opinion to compensate for the trade-off in specificity.

To select between dictionaries generated between 50, 33 and 25 annotation cut-offs, and also to ensure the terms that are selected as enriched by our algorithm are reasonable, we looked in detail at the enrichment analysis results. Most results were comparable and expected. For some sets, all dictionaries performed well. For example, in our ‘all neuron enriched sets’ [18, 20] all terms were neuron-related regardless of the dictionary used (see Table 2). On the other hand, for a set enriched for germline precursor expression in the embryo [18], the 50 cutoff dictionary was only able to identify ‘oocyte WBbt:006797’, which is not a germline precursor although it is germline related; whereas the two smaller dictionaries singled out actual germline precursor cells—at the 33 cutoff, our tool identified the larval germline precursor cells ‘Z2’ and ‘Z3’ as enriched, and at the 25 gene cutoff the embryonic germline precursor terms ‘P_4_’,‘P_3_’ and ‘P_2_’ were identified in addition to ‘Z2’ and ‘Z3’. We also queried an intestine precursor set [18]. Notably, this gene set yielded no enrichment when using the 25 cutoff dictionary, nor when using the 50 cutoff dictionary. However, the 33 cutoff dictionary identified the E lineage, which is the intestinal precursor lineage in *C. elegans*, as enriched. Both of these results capture specific aspects of *C. elegans* that are well known to developmental biologists.

**Table 2:** Comparison of results for a GABAergic neuronal-enriched gene set from Watson [20] showing that results are similar regardless of annotation cutoff

Tissue	Q value33	Q value50	Enrichment fold change33	Enrichment fold change50
Nerve ring WBbt:0006749	0.005	0.0015	2.7	2.7
Nervous system WBbt:0005735	0.005	0.0015	1.4	1.4
Dorsal nerve cord WBbt:0006750	0.005	0.0015	3.8	3.8
Retrovesicular ganglion WBbt:0005656	0.011	0.0034	5	5
Ventral nerve cord WBbt:0005829	-	0.022	-	2.4

We ran the same gene list on a dictionary with a minimum annotation cutoff of 50, similarity threshold of 0.95 and similarity method ‘any’ versus another with a minimum annotation cutoff of 33, similarity threshold of 0.95 and similarity method ‘any’. In the table, columns are labeled with their significance value (*Q*-value) or enrichment fold change followed by a hyphen and a number which indicates which the cutoff for the dictionary that was used for testing. Not all tissues are present in either dictionary. Hyphens denote not-applicable values, which occurs when a particular tissue is not present in both dictionaries

Not all queries worked equally well. For example, a number of intestinal sets [18, 21] were not enriched in intestine-related terms in any dictionary, but were enriched for pharynx and hypodermis. We were surprised that intestinal gene sets performed poorly, since the intestine is a relatively well-annotated tissue.

**Fig. 5:**
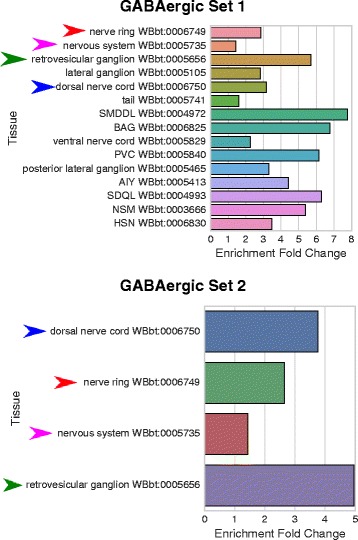
Independently derived gene sets show similar results when tested with the same dictionary. Set 1) GABAergic gene set from Watson [20]. Set 2) GABAergic gene set from Spencer [18]. Arrowheads highlight identical terms between both analyses. All terms refer to neurons or neuronal tissues and are GABA-associated. Dictionary with cutoff: 33; threshold: 0.95; method: ‘any’

We assessed the internal agreement of our tool by using independent gene sets that we expected to be enriched in the same tissues. We used two pan-neuronal sets [18, 20]; two PVD sets [18, 24]; and two GABAergic sets [18, 19]. Overall, the tool has good internal agreement. On most sets, the same terms were enriched, although order was somewhat variable (see Fig. 5), and most high-scoring terms were preserved between sets. All comparisons can be found online in our Github repository (see Availability of data and materials). The complete list of gene sets and results can also be found in Additional files 2, 3 and 4. Overall, the dictionary generated by a 33 gene annotation cutoff with 0.95 redundancy threshold using the ‘any’ criterion performed best, with a good balance between specificity, verbosity and accuracy, so we selected this parameter set to generate our static dictionary. As of this publication, the testable dictionary contains 261 terms.

### Applying the tool

**Fig. 6:**
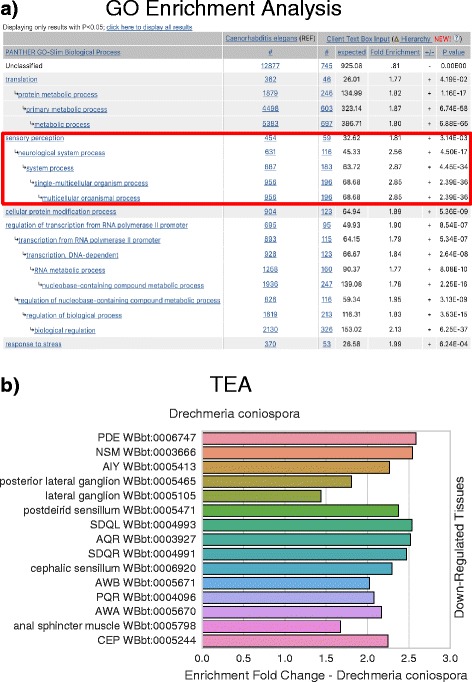
*D. coniospora* gene enrichment analysis and tissue enrichment analysis results. We compared and contrasted the results from a gene enrichment analysis program, pantherDB, with TEA by analyzing genes that were significantly down-regulated when *C. elegans* was exposed to *D. coniospora* in a previously published dataset by Engelmann et al. [25] with both tools. **a** pantherDB screenshot of results, sorted by *p*-value. Only top hits shown. **b** TEA results, sorted by *q*-value (lowest on top) and fold-change. Both pantherDB and TEA identify terms associated with neurons (*red square*). The two analyses provide complementary, not redundant, information

We applied our tool to the RNA-seq datasets developed by Engelmann et al. [25] to gain further understanding of their underlying biology. Engelmann et al. exposed young adult worms to 5 different pathogenic bacteria or fungi for 24 h, after which mRNA was extracted from the worms for sequencing. We ran TEA on the genes Engelmann et al. identified as up- or down-regulated. Initially we noticed that genes that are down-regulated tend to be twice as better annotated on average than genes that were up-regulated, suggesting that our understanding of the worm immune system is scarce, in spite of important advances made over the last decade. Up-regulated tissues, when detected, almost always included the hypodermis and excretory duct. Three of the five samples showed enrichment of neuronal tissues or neuronal precursor tissues among the down-regulated genes. As an independent verification, we also performed GO analysis using PANTHER on the down-regulated genes for *D. coniospora*. These results also showed enrichment in terms associated with neurons (see Fig. 6). A possible explanation for this neuronal association might be that the infected worms are sick and the neurons are beginning to shut down; an alternative hypothesis would be that the worm is down-regulating specific neuronal pathways as a behavioral response against the pathogen. Indeed, several studies [26, 27] have provided evidence that *C. elegans* uses chemosensory neurons to identify pathogens. Our results highlight the involvement of various *C. elegans* neuronal tissues in pathogen defense.

## Discussion

We have presented a tissue enrichment analysis tool that employs a standard hypergeometric model to test the *C. elegans* tissue ontology. We use a hypergeometric function to test a user-provided gene list for enrichment of anatomical terms in *C. elegans*. Our hope is that the physiological relevance of anatomical terms will enable researchers to make hypotheses about high-dimensionality data. Specifically, we believe an enriched term may broadly suggest one of two hypotheses: if a list is enriched in a particular anatomical region, that anatomical region is affected by the experimental treatment; alternatively, the anatomical regions that are enriched reflect biologically relevant interactions between tissues. We believe the first hypothesis is a reasonable one to make in the case of whole-worm RNA-seq data for example, whereas the second hypothesis may be more plausible in cases where a researcher already knows what tissues a particular gene list came from, as may be the case in single-cell RNA-seq.

Our tool relies on an annotation dictionary that is continuously updated primarily with data from single gene qualitative analyses, does not require retraining and does not require ranked genes. To our knowledge, this is the first tool that tests tissue enrichment in *C. elegans* via the hypergeometric method, but similar projects exist for humans and zebrafish [28, 29], highlighting the relevance of our tool for high-dimensionality biology. Chikina et al. [30] have previously reported a tissue enrichment model for *C. elegans*based on a Support Vector Machine classifier that has been trained on microarray studies. SVMs are powerful tools, but they require continuous retraining as more tissue expression data becomes available. Moreover, classifiers require that data be rank-ordered by some metric, something which is not possible for certain studies. Furthermore, this tissue enrichment tool provides users with enrichment results for only 6 large tissues. In contrast, our tool routinely tests a much larger number of terms, and we have shown it can even accurately identify enrichment of embryonic precursor lineages for select data sets.

We have also presented the first, to our knowledge, ontology term filtering algorithm applied to biomedical ontologies. This algorithm, which is very easy to execute, identifies terms that have specificity and statistical power for hypothesis testing. Due to the nature of all ontologies as hierarchical, acyclical graphs with term inheritance, term annotations are correlated along any given branch. This correlation reduces the benefits of including all terms for statistical analysis: for any given term along a branch, if that term passes significance, there is a high probability that many other terms along that branch will also pass significance. If the branch is enriched by random chance, error propagation along a branch means that many more false positives will follow. Thus, a researcher might be misled by the number of terms of correlated function and assign importance to this finding; the fact that the branching structure of GO amplifies false positive signals is a powerful argument for either reducing branch length or branch intracorrelation, or both. On the other hand, if a term is actually enriched, we argue that there is little benefit to presenting the user with additional terms along that branch. Instead, a user will benefit most from testing sparsely along the tree at a suitable specificity for hypothesis formation. Related terms of the same level should only be tested when there is sufficient annotation to differentiate, with statistical confidence, whether one term is enriched above the other. Our algorithm reduces branch length by identifying and removing nodes that are insufficiently annotated and parents that are likely to include sparse information.

We endeavoured to benchmark our tool well, but our analysis cannot address problems related to spurious term enrichment. Although we were unable to determine false-positive and false-negative rates, we do not believe this should deter scientists from using our tool. Rather, we encourage researchers to use our tool as a guide, integrating evidence from multiple sources to inform the most likely hypotheses. As with any other tool based on statistical sampling, our analysis is most vulnerable to bias in the data set. For example, expression reports are negatively biased against germline expression because of the difficulties associated with expressing transgenes in this tissue [31]. As time passes, we are certain the accuracy and power of this tool will improve thanks to the continuing efforts of the worm research community; indeed, without the community reports of tissue expression in the first place, this tool would not be possible.

## Conclusions

We have built a tissue enrichment tool that employs a tissue ontology previously developed by WormBase. We use a simple algorithm to identify the best ontology terms for statistical testing and in this way minimize multiple testing problems. Our tool is available within WormBase or can be downloaded for offline use via ‘pip install’.

## Methods

### Fetching annotation terms

We used WormBase-curated gene expression data, which includes annotated descriptions of spatial-temporal expression patterns of genes, to build our dictionary. Gene lists per anatomy term were extracted from a Solr document store of gene expression data from the WS252 database provided by WormBase [8]. We used the Solr document store because it provided a convenient access to expression data that included inferred annotations. That is, for each anatomy term, the expression gene list includes genes that were directly annotated to the term, as well as those that were annotated to the term’s descendant terms (if there were any). Descendant terms were those connected with the focus term by is_a/part_of relationship chains defined in the anatomy term ontology hierarchy.

### Filtering nodes

#### Defining a similarity metric

To identify redundant sisters, we defined the following similarity metric:

2 $$\begin{array}{@{}rcl@{}} s_{i} = \frac{|g_{i}|}{|\bigcup_{i= 0}^{k} g_{i}|} \end{array} $$

Where *s*_*i*_ is the similarity for a tissue *i* with *k* sisters; *g*_*i*_ refers to the set of tissues associated with tissue *i* and |*g*| refers to the cardinality of set *g*. For a given set of sisters, we called them redundant if they exceeded a given similarity threshold. We envisioned two possible criteria and built different dictionaries using each one. Under a threshold criteron ‘any’ with parameter *S* between (0,1), a given set of sisters *j* was considered redundant if the condition

3 $$\begin{array}{@{}rcl@{}} s_{i, j} > S \end{array} $$

was true for any sister *i* in set *j*. Under a threshold criterion ‘avg’ with parameter *S*, a given set of sisters *j* was considered redundant if the condition

4 $$\begin{array}{@{}rcl@{}} \mathrm{E}[s_{i}]_{j} > S \end{array} $$

was true for the set of sisters *j* (see Fig. 1a).

Since nodes can have multiple parents (and therefore multiple sister sets), a complete set of similarity scores was calculated before trimming the ontology, and nodes were removed from the ontology if they exceeded the similarity threshold at least once in any comparison.

#### Implementation

All scripts were written in Python 3.5. Our software relies on the pandas, NumPy, Seaborn and SciPy modules to perform all statistical testing and data handling [32–34].

## Additional files

Additional file 1 TEA Tutorial. Tutorial for users interested in using our software within a python script. (PDF 161 kb)

Additional file 2 Folder Structure for SI files 3 and 4. A file detailing the folder structure of the zipped folders 3 and 4. (PDF 138 kb)

Additional file 3 Golden Gene Sets. A list of all the genes used for our benchmarking process. (ZIP 74 kb)

Additional file 4 Results. A folder containing a complete version of the results we generated for this paper. (ZIP 1597 kb)

## Abbreviations

GO: Gene ontology
TEA: Tissue enrichment analysis
WBbt ID: A unique ID assigned to reference ontology terms
WBgene ID: A unique ID assigned to reference nematode genes
