# Supplementary material for: Tissue enrichment analysis for C. elegans genomics
Source: BMC Bioinformatics. 2016 Sep 13;17(1):366. doi: 10.1186/s12859-016-1229-9 (PMC5020436; doi:10.1186/s12859-016-1229-9)
Supplement: Additional file 1 — TEA Tutorial. Tutorial for users interested in using our software within a python script. (PDF 161 kb) [file 12859_2016_1229_MOESM1_ESM.pdf]

TEA Tutorial

April 25, 2016

1 An IPython Introduction to Using TEA for C. elegans researchers

All of the code below was written by David Angeles-Albores. Should you find any errors, typos, or just have general comments, please contact:

dangeles at caltech dt edu

The work here was submitted and accepted for publication on . . . . Please cite **Tissue Enrichment Analysis for C. elegans Genomics** if this notebook was useful for you in your research.

Please note: I have tried to make this tutorial as complete as possible, with a brief introduction to Pandas dataframes and showing how I typically prepare my dataframes for analysis. Experienced users will want to skip this and go straight to **Calling TEA**. However, this tutorial is by no means a complete introduction to Python or Pandas - in fact, it's more like a super fast crash course. I apologize for this, and in the future I will consider improving the tutorial.

1.0.1 Best of Luck!

– David Angeles-Albores

2 Introduction

2.0.1 What is TEA meant for?

TEA is meant to provide straightforward analysis of large gene lists for C. elegans researchers. We hope that TEA will function as a hypothesis generator, or alternatively, as a way of understanding the biology behind a dataset.

2.0.2 How is TEA different from GO?

Great question. GO is primarily a molecular/cellular ontology, whereas TEA works from TO, the C. elegans tissue ontology. I believe tissues are, in some senses, fundamental units in biology. Often, it is the case that tissues, not cells, have been studied for considerably longer time, and as a result we have a better intuition for what the function of a tissue is, as compared to the molecular function of a list of genes. In other words, I think GO analysis and TEA are similar, but my guess is that the results from TEA will be easier to interpret, and as a result easier to use for hypotheiss generation.

2.0.3 What TEA is not:

TEA is NOT meant to be used as a quantitative tool!

At best, TEA is a very good guess about what tissues are being affected in your dataset. At worst, TEA is a guess about what tissues are being affected in your dataset. TEA is working directly from the WormBase-curated expression dataset. As a result, we have the very best, most up to date annotations in the world. On the other hand, please remember these annotations suffer from bias. For example, the ASE, ASK and ASI neurons have been very well studied and are quite well annotated, but the individual intestinal

cells have not been generally well studied! Thus, our annotations are significantly biased by the research community's interests.

Please use TEA carefully, and always use it as a guiding tool for your research, and never as the final say on anything.

2.0.4 What do you need to do to run this tool?

The gist of the algorithm is:
Get your gene list into WBIDs
Call our analysis function
Call the plotting function
Done.

2.1 Batch users:

This script runs on Python > 3.5.

Dependencies: scipy (all), pandas, numpy, matplotlib and seaborn

If you have pip, do

```
pip install tissue_enrichment_analysis
```

to install the library in your computer.

Import the module. You may find that the **numpy** and **pandas** modules are also often very useful.

For the purposes of this journal, the file structure I'm working with is the following:

src - the folder this file lives in input - a folder that contains all my input files. Also contains

Engelmann - folder containing the files i will be using

```
In [6]: import tissue_enrichment_analysis as tea #the main library for this tutorial
        import pandas as pd
        import os
        import importlib as imp
        import numpy as np
        import seaborn as sns
        import matplotlib.pyplot as plt

        #to make IPython plot inline, not req'd if you're not working with an Ipython notebook
        %matplotlib inline
```

Now let's import our dataset. Here, I will use a dataset I obtained from **Engelmann et al, 2011 (PLOS One)**.

Specifically, this is data from an RNA-seq experiment they performed. Briefly, young adult worms were placed in D. coniospora fungus for 24, cleaned and then RNA-seq'ed.

```
In [19]: dfDcon= pd.read_csv('../input/Engelmann/coniospora_Engelmann_2011.csv') #Don't forget to chang
```

Let's visualize the first five lines of the dataframe to see what's in it

```
In [20]: print('This dataframe has {0} columns and {1} rows'.format(dfDcon.shape[1], dfLum.shape[0]))
         dfDcon.head()
```

```
This dataframe has 9 columns and 27322 rows
```

```
Out[20]:   GenePublicName SequenceNameGene SequenceNameTranscript        DC      OP50  \
         0         2L52.1           2L52.1                 2L52.1  0,080068   0,07456
         1        2RSSE.1          2RSSE.1                2RSSE.1  0,639852  0,586729
         2        2RSSE.2          2RSSE.2                2RSSE.2  0,080266  0,081083
```

```
3          3R5.1           3R5.1                3R5.1  0,270145  0,209694
4          nas-6          4R79.1              4R79.1a  0,000617  0,000749

   Ratio_DCvsOP50  log2_Ratio_DCvsOP50  Infection_upregulated  \
0  1.073868970660             0.102818                    NaN
1  1.090540948206             0.125044                    NaN
2  0.989921488245            -0.014614                    NaN
3  1.288281972779             0.365448                    NaN
4  0.824236379271            -0.278870                    NaN

   Infection_downregulated
0                      NaN
1                      NaN
2                      NaN
3                      NaN
4                      NaN
```

Ok. Clearly we can see the dataframe has a few different columns. Of particular interest to use are the columns 'Infection_upregulated' and 'Infection_downregulated', since these are the genes they identified as significantly altered by the treatment relative to an OP50 control. Let's analyze the genes that are upregulated first and see what they can do.

Before we can analyze anything, notice that they don't list WBIDs anywhere. We need to turn the names into WBIDs before we can continue.

To do this, I will load another file containing all the WBID-human readable relationships into a new dataframe called names

In [21]:
```
names= pd.read_csv('../input/Engelmann/c_elegans.PRJNA13758.WS241.livegeneIDs.unmaprm.txt',
                    sep= '\t',comment= '#')
```

Let's take a look at it:

In [22]:
```
print('The length of this dataframe is:{0}'.format(len(names)))
names.head()
```

```
The length of this dataframe is:46788
```

Out[22]:

	WBID	HumanReadable	GeneName
0	WBGene00000001	aap-1	Y110A7A.10
1	WBGene00000002	aat-1	F27C8.1
2	WBGene00000003	aat-2	F07C3.7
3	WBGene00000004	aat-3	F52H2.2
4	WBGene00000005	aat-4	T13A10.10

The Engelmann names look like they are GeneNames.

Next, I'm going to generate a lambda function. This function will take a single argument 'x'. 'x' should the be the column containing the names we want to convert into WBIDs. Once we provide 'x', this function will look in the GeneName column of the names dataframe to see whether a particular entry can be found in the GeneName column.

For every entry it can find, g returns True. Else, it returns False

In [23]:
```
g= lambda x: (names.GeneName.isin(x))
```

Let's try our new function out!

```
In [24]: #Remember, dfLum is the dataframe. dfLum['SequenceNameGene'] is the column we want.#
         #We store the result in a variable called 'translate'
         translate= g(dfDcon['SequenceNameGene'])
         #I only want to show the first 5 rows, so I'm going to add [0:5] after translate, since 'g' re
         print(translate[0:5])
```

```
0    True
1    True
2    True
3    True
4    True
Name: GeneName, dtype: bool
```

Great! Now we can get the WBIDs by simple indexing:

```
In [25]: wbids= names[translate].WBID # names[translate] gets rows for every gene name that was found b
         #The .WBID after names[] tells the computer to get the WBID colum
```

```
In [26]: print('wbids has {} gene IDS. The original dataframe has {} genes'.format(len(wbids), dfLum.sha
         wbids.head() #let's see what we found
```

```
wbids has 19702 gene IDS. The original dataframe has 27322 genes
```

```
Out[26]: 0    WBGene00000001
         1    WBGene00000002
         2    WBGene00000003
         3    WBGene00000004
         4    WBGene00000005
         Name: WBID, dtype: object
```

Hmmm. We lost quite a few genes. Let's quickly check to make sure those aren't important

```
In [27]: not_found= dfDcon[~dfDcon.SequenceNameGene.isin(names[translate].GeneName)]
         not_found.head()
```

```
Out[27]:     GenePublicName SequenceNameGene SequenceNameTranscript        DC  \
         2          2RSSE.2          2RSSE.2                2RSSE.2  0,080266
         7           6R55.1           6R55.1                6R55.1a    1,6612
         8           6R55.1           6R55.1                6R55.1b   1,66481
         197        B0212.6          B0212.6                B0212.6  0,015785
         416        B0310.4          B0310.4                B0310.4         0

                  OP50  Ratio_DCvsOP50  log2_Ratio_DCvsOP50  Infection_upregulated  \
         2    0,081083  0.989921488245            -0.014614                    NaN
         7     1,38576  1.198764576839             0.261548                    NaN
         8     1,38919  1.198403386146             0.261114                    NaN
         197  0,007612  2.073554738456             1.052106                    1.0
         416         0             NaN                  NaN                    NaN

              Infection_downregulated
         2                        NaN
         7                        NaN
         8                        NaN
         197                      NaN
         416                      NaN
```

A quick search in WormBase shows that these genes have been merged into other genes. Hmmmm.. This could be a problem.

To figure out if it really is a problem, let's look at how many of those genes are upregulated during infection.

```
In [28]: print('There are {0} upregulated genes, of which {1} can\'t be found in the names dictionary'.
                dfDcon[dfDcon.Infection_upregulated == 1].shape[0], not_found[not_found.Infection_upregul


         print('{0:.2}% could not be found'.format(
                  not_found[not_found.Infection_upregulated == 1].shape[0]/dfDcon[dfDcon.Infection_upreg

There are 1692 upregulated genes, of which 31 can't be found in the names dictionary
0.018% could not be found
```

Great! So there's almost no loss in our gene name conversion. Now we can go ahead and extract all the IDs that we can find to use for our enrichment analysis

```
In [37]: translate= g(dfLum[dfLum.Infection_downregulated == 1]['SequenceNameGene'])
         wbids= names[translate].WBID

In [38]: print(wbids.head())

2     WBGene00000003
4     WBGene00000005
5     WBGene00000006
19    WBGene00000021
36    WBGene00000038
Name: WBID, dtype: object
```

See how the list changed from before? Great! Now we can put this into TEA

3 Calling TEA

TEA works by comparing your gene-list to a reference tissue expression ''dictionary". In order for us to run TEA, we first need to fetch the dictionary. That's done easily enough:

```
In [33]: tissue_df= tea.fetch_dictionary() #this downloads the tissue dictionary we want

In [35]: tissue_df.head()

Out[35]:              wbid  ray 2 WBbt:0006945  Cpapa WBbt:0005962  \
         0  WBGene00003681                0.0                 0.0
         1  WBGene00000023                0.0                 0.0
         2  WBGene00022837                0.0                 0.0
         3  WBGene00003905                0.0                 0.0
         4  WBGene00003983                0.0                 0.0

            ABarappa WBbt:0006005  ABprpppap WBbt:0006237  Cpaaa WBbt:0006212  \
         0                    0.0                     0.0                 0.0
         1                    0.0                     0.0                 0.0
         2                    0.0                     0.0                 0.0
         3                    0.0                     0.0                 0.0
         4                    0.0                     0.0                 0.0

            pm6 WBbt:0003724  ventral nerve cord WBbt:0005829  ABalpaap WBbt:0005934  \
```

```
0              0.0                              0.0                      0.0
1              0.0                              0.0                      0.0
2              0.0                              0.0                      0.0
3              0.0                              0.0                      0.0
4              0.0                              0.0                      0.0

   retrovesicular ganglion WBbt:0005656          ...            \
0                                    0.0         ...
1                                    0.0         ...
2                                    0.0         ...
3                                    0.0         ...
4                                    0.0         ...

   ABprpappa WBbt:0006088  MSaapp WBbt:0006425  \
0                     0.0                  0.0
1                     0.0                  0.0
2                     0.0                  0.0
3                     0.0                  0.0
4                     0.0                  0.0

   thermosensory neuron WBbt:0005838  postdeirid sensillum WBbt:0005471  \
0                                0.0                                  0.0
1                                0.0                                  0.0
2                                0.0                                  0.0
3                                0.0                                  0.0
4                                0.0                                  0.0

   ABprappp WBbt:0006702  AWA WBbt:0005670  ray 9 WBbt:0006954  \
0                    0.0               0.0                 0.0
1                    0.0               0.0                 0.0
2                    0.0               0.0                 0.0
3                    0.0               0.0                 0.0
4                    0.0               0.0                 0.0

   ABprappaa WBbt:0006350  nociceptor neuron WBbt:0008434  \
0                     0.0                             0.0
1                     0.0                             0.0
2                     0.0                             0.0
3                     0.0                             0.0
4                     0.0                             0.0

   ABpraapa WBbt:0006302
0                    0.0
1                    0.0
2                    0.0
3                    0.0
4                    0.0

[5 rows x 261 columns]
```

Quick technical note: We could have placed the dictionary inside the other functions and call them from the inside, but we want you to be able to access the dictionary. Why? Well, you might imagine that you want to get all the genes that are specifically expressed in a tissue, or you may want to take a look at what tissues are included, etc. . .

In other words, we want you to be able to get your hands on this data! It's up to date, it's easy and it works beautifully.

Now that we have the dictionary, we can run the enrichment analysis. Just so you know what's going on when you call it, the function has the following args.:

enrichment_analysis(gene_list, tissue_df, alpha= 0.05, aname= ", save= False, show= True)

Most of these you can ignore. Mainly, you'll want to assign:

gene_list = your gene list

tissue_df = the result from fetch_dictionary()

alpha= your desired q-value threshold

aname= if you want to save the result to your python interpret, give it a name and complete path

save= if you want to save your file, you must set this to True

This function returns 2 things:

df_res – a dataframe with all the results

unused – a list of all the genes that were discarded from the analysis

For now, let's jsut run the analysis and show it here:

```
In [39]: df_res, unused= tea.enrichment_analysis(wbids, tissue_df, show= True, save= False)
```

```
Executing script

                                          Tissue   Expected  Observed  \
0   posterior lateral ganglion WBbt:0005465  20.460474      37.0
5                         PDE WBbt:0006747   5.416008      14.0
6                         AIY WBbt:0005413   9.267391      21.0
7             lateral ganglion WBbt:0005105  61.140711      88.0
9                         NSM WBbt:0003666   5.897431      15.0
8         postdeirid sensillum WBbt:0005471   5.897431      14.0
11                       SDQL WBbt:0004993   4.332806      11.0
2                        SDQR WBbt:0004991   4.453162      11.0
4           cephalic sensillum WBbt:0006920   5.656719      13.0
10                        AQR WBbt:0003927   3.971739      10.0
13                        AWB WBbt:0005671   8.906324      18.0
12                        PQR WBbt:0004096   6.739921      14.0
1                         AWA WBbt:0005670   5.536364      12.0
3        anal sphincter muscle WBbt:0005798  15.525889      26.0

    Enrichment Fold Change   Q value
0                 1.808365  0.016980
5                 2.584930  0.016980
6                 2.266010  0.016980
7                 1.439303  0.016980
9                 2.543480  0.016980
8                 2.373915  0.018099
11                2.538770  0.024781
2                 2.470155  0.028501
4                 2.298152  0.028501
10                2.517789  0.028501
13                2.021036  0.028501
12                2.077176  0.041767
1                 2.167488  0.045186
3                 1.674622  0.046300
```

Voila! We got our results. Great! But what if we didn't want to show them?'

```
In [40]: df_res, unused= tea.enrichment_analysis(wbids, tissue_df, show= False, save= False)
```

```
Executing script
```

We could still look at the results by typing df_res.head():

```
In [41]: df_res.head()
```

```
Out[41]:                                   Tissue   Expected  Observed  \
         0  posterior lateral ganglion WBbt:0005465  20.460474      37.0
         5                        PDE WBbt:0006747   5.416008      14.0
         6                        AIY WBbt:0005413   9.267391      21.0
         7            lateral ganglion WBbt:0005105  61.140711      88.0
         9                        NSM WBbt:0003666   5.897431      15.0

            Enrichment Fold Change  Q value
         0                1.808365  0.01698
         5                2.584930  0.01698
         6                2.266010  0.01698
         7                1.439303  0.01698
         9                2.543480  0.01698
```

What about the unused genes? Let's see how many of those there are:

```
In [42]: print('{0} were discarded from the analysis'.format(len(unused)))
```

```
4451 were discarded from the analysis
```

Ouch! That's a lot! Don't like it? Make GFP reporters and let WormBase know where they are expressed. **Seriously. Do it! You'd be helping the whole community a lot!**

Now let's plot the results

```
In [43]: tea.plot_enrichment_results(df_res, title= 'Exercise', save= False)
```

```
Out[43]: <matplotlib.axes._subplots.AxesSubplot at 0x11ab54160>
```

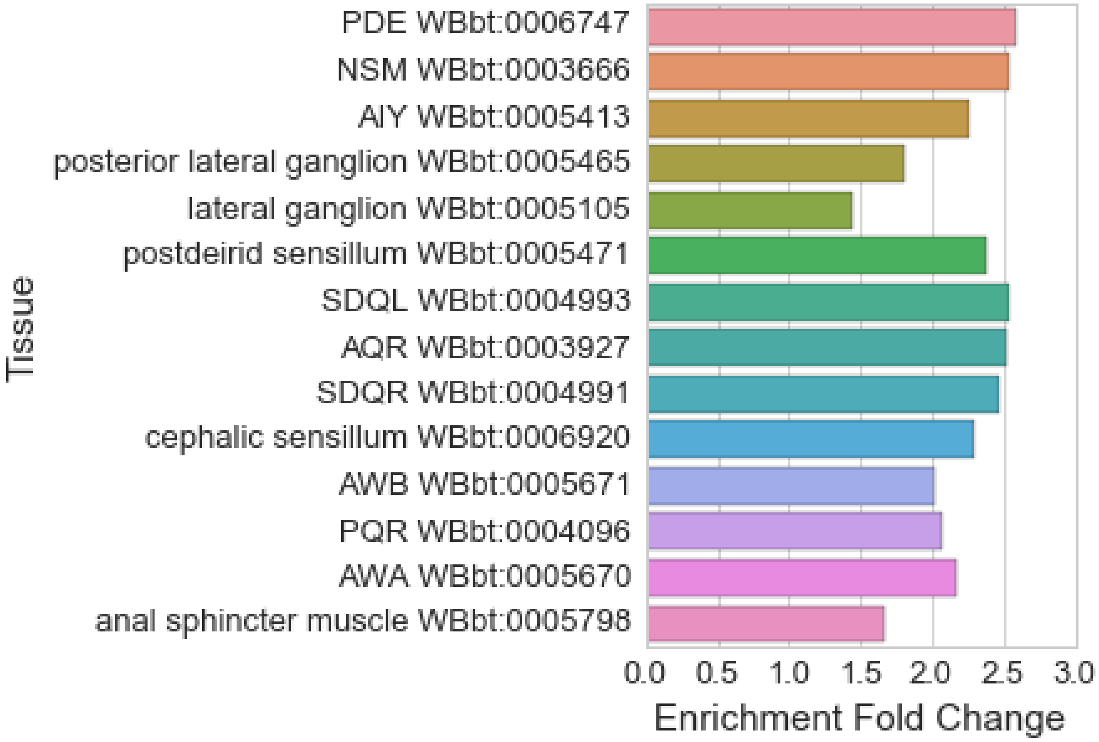

Voila! We've analyzed our data! Yay! :D

If we wanted to save our plot, we would type:

```
In [ ]: tea.plot_enrichment_results(df_res, title= 'Exercise', save= True, dirGraphs= 'example_graph_di
        #This will save the graph in the corresponding directory. If no directory is specified, the gra
        #to the current working directory.
```
