# Supplementary material for: Tissue enrichment analysis for C. elegans genomics
Source: BMC Bioinformatics. 2016 Sep 13;17(1):366. doi: 10.1186/s12859-016-1229-9 (PMC5020436; doi:10.1186/s12859-016-1229-9)
Supplement: Additional file 2 — Folder Structure for SI files 3 and 4. A file detailing the folder structure of the zipped folders 3 and 4. (PDF 138 kb) [file 12859_2016_1229_MOESM2_ESM.pdf]

Folder Structure

In the SI, we provide the entire output of our tests for the interested reader. We provide two folders. The gene set folder contains all the gene sets used to benchmark our tool. Gene sets are named according to a common schema. Files are named
`WBPaperID_AnatomyTermEnriched(HumanReadable)_AnatomyTermWBbtID_NumberOfGenesInSet.csv`.

The results folder contains all the results generated by our analysis. The results folder is organized as follows (bolded text refers to a folder, otherwise files are listed):

- **Engelmann**
- **HGT25_any_Results**
- **HGT33_any_Results**
- **HGT50_any_Results**
- **HGT100_any_Results**
- **SummaryInformation**
- **comparisons**
- test_list_efaecelis.txt

HGTXX_any_Results

The folders titled **HGTXX_any_Results** contain the enrichment results of all the 30 golden sets tested with a dictionary with cutoff **XX**, similarity threshold= 0.95 and thresholding method 'any'.

The folders contain two kinds of files, '.pdf' and '.csv' files. The pdf files contain the bar charts for each analysis, whereas the csv files contain the complete output for a particular geneset. There is also a file called 'empty.txt' that contains the names of any gene sets where no terms were enriched.

Files are titled according to a common schema. For example, the file
`WBPaper00013489_Ray_Enriched_WBbt_0006941_25.csv`
refers to the *WormBase Paper 0013489*, which should be enriched in '*Ray Enriched (WBbt:0006941)*' and contains *25* genes. The gene set that was used for this analysis is contained in the SI folder named '*golden gene sets*' and is contained in a homologously named csv file.

Engelmann

Contains all graphs pertaining to the data from Engelmann 2011.

Comparisons

Contains csv files of the comparisons between gene sets or within dictionaries. The file nomenclature is:
`neuronal_comparison_33_WBPaper00024970_with_WBPaper0037950_complete.csv`
Refers to a 'neuronal comparison' using dictionary 33, comparing papers 24970 with 37950. The word complete at the end of the analysis refers to the fact that this table contains the complete table of results.

Alternatively, when comparing two dictionaries, the nomenclature is:
`neuronal_comparison_GABAergic_33-50_WBPaper0037950_complete.csv`
Which refers to a 'GABAergic neuronal' lists, comparing dictionaries with cutoffs of 33-50 (all other parameters are the same) using paper 37950.

The complete version of table 2 in the paper is
`neuronal_comparison_33_WBPaper00024970_with_WBPaper0037950_complete.csv`

Summary Info

This folder contains

- ExecutiveSummary.csv — a table containing summary information ofthe results of all the enrichment analysis we performed. The table contains the parameters of the dictionary used to run the analysis (cutoff, similarity threshold and thresholding method), the name of the gene set analysed, the number of terms tested, the number of genes submitted for analysis, the number of terms that tested significant, the average fold change of terms that tested significant, the average q-value of terms that tested significant and the number of genes in the dictionary.
- TissueNumbers.csv — A table showing the number of terms in each dictionary that was generated.
- avgFoldChange.pdf — KDE of the average fold change value for each dictionary
- avgQKDE_method=any.pdf — KDE of the average q-value for each dictionary
- avgQKDE_method=avg.pdf — KDE of the average fold change value for each dictionary
- fractissuesKDE_method=any.pdf — KDE of the average number of tissues in the dictionary that tested significant for each dictionary.

test_list_efaecalis.txt

This file was used to generate figure 2 in the text and is a slightly processed version of Engelmann's downregulated genes for *E. faecalis* (this list was processed into WBIDs).
