# Supplementary material for: Tissue enrichment analysis for C. elegans genomics
Source: BMC Bioinformatics. 2016 Sep 13;17(1):366. doi: 10.1186/s12859-016-1229-9 (PMC5020436; doi:10.1186/s12859-016-1229-9)
Supplement: Additional file 4 — Results. A folder containing a complete version of the results we generated for this paper. (ZIP 1597 kb) [file 12859_2016_1229_MOESM4_ESM.zip › output/Engelmann/Graphs/Enterococcus faecalisEnrichment.pdf]

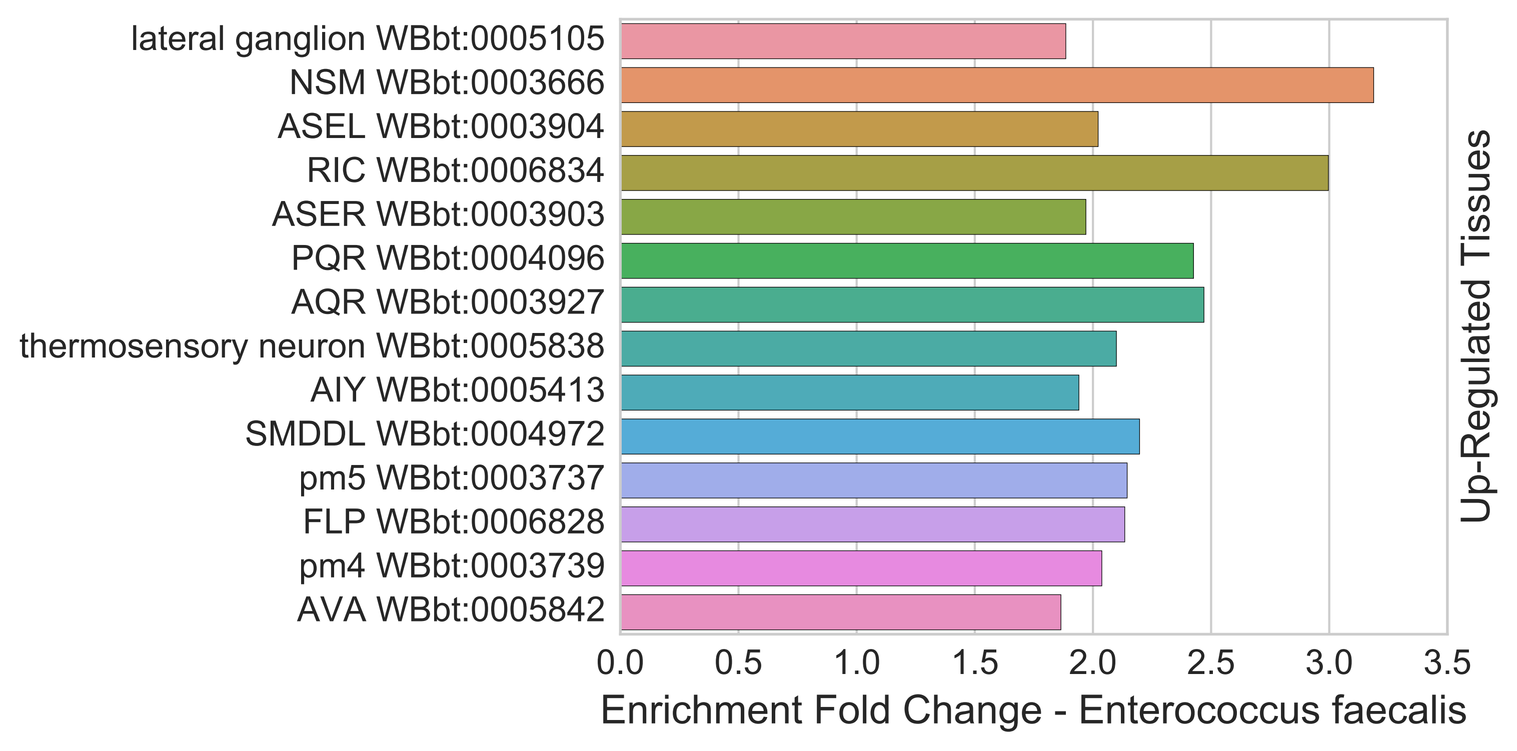
lateral ganglion WBbt:0005105
NSM WBbt:0003666
ASEL WBbt:0003904
RIC WBbt:0006834
ASER WBbt:0003903
PQR WBbt:0004096
AQR WBbt:0003927
thermosensory neuron WBbt:0005838
AIY WBbt:0005413
SMDDL WBbt:0004972
pm5 WBbt:0003737
FLP WBbt:0006828
pm4 WBbt:0003739
AVA WBbt:0005842
0.0
0.5
1.0
1.5
2.0
2.5
3.0
3.5
Enrichment Fold Change - Enterococcus faecalis
Up-Regulated Tissues
