# Supplementary material for: Tissue enrichment analysis for C. elegans genomics
Source: BMC Bioinformatics. 2016 Sep 13;17(1):366. doi: 10.1186/s12859-016-1229-9 (PMC5020436; doi:10.1186/s12859-016-1229-9)
Supplement: Additional file 4 — Results. A folder containing a complete version of the results we generated for this paper. (ZIP 1597 kb) [file 12859_2016_1229_MOESM4_ESM.zip › output/HGT33_any_Results/WBPaper00031003_24hr_muscle_enriched_WBbt_0003675_918.pdf]

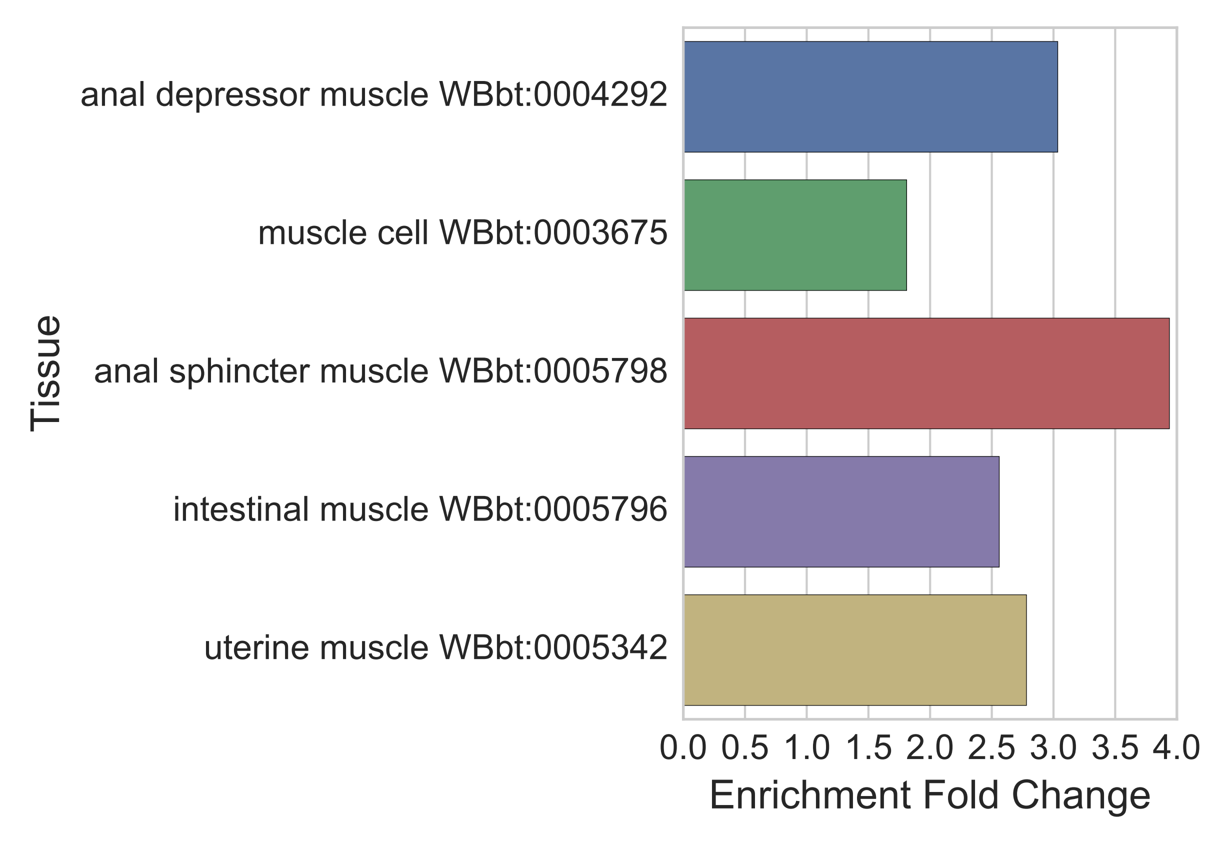
anal depressor muscle WBbt:0004292
muscle cell WBbt:0003675
anal sphincter muscle WBbt:0005798
intestinal muscle WBbt:0005796
uterine muscle WBbt:0005342
Tissue
0.0 0.5 1.0 1.5 2.0 2.5 3.0 3.5 4.0
Enrichment Fold Change
