# Supplementary material for: Tissue enrichment analysis for C. elegans genomics
Source: BMC Bioinformatics. 2016 Sep 13;17(1):366. doi: 10.1186/s12859-016-1229-9 (PMC5020436; doi:10.1186/s12859-016-1229-9)
Supplement: Additional file 4 — Results. A folder containing a complete version of the results we generated for this paper. (ZIP 1597 kb) [file 12859_2016_1229_MOESM4_ESM.zip › output/HGT33_any_Results/WBPaper00037950_GABAergic-motor-neurons_larva_enriched_WBbt_0005190_132.pdf]

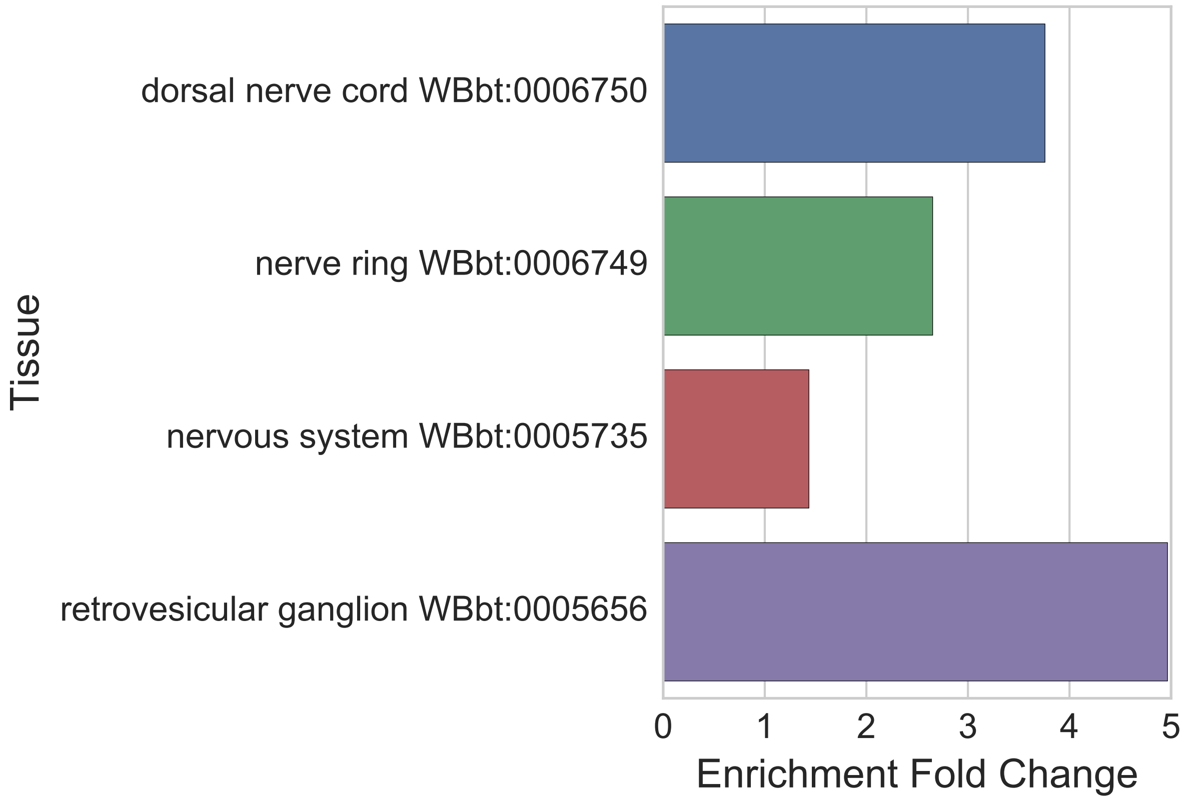
dorsal nerve cord WBbt:0006750
nerve ring WBbt:0006749
nervous system WBbt:0005735
retrovesicular ganglion WBbt:0005656
Tissue
0
1
2
3
4
5
Enrichment Fold Change
