# Supplementary material for: Tissue enrichment analysis for C. elegans genomics
Source: BMC Bioinformatics. 2016 Sep 13;17(1):366. doi: 10.1186/s12859-016-1229-9 (PMC5020436; doi:10.1186/s12859-016-1229-9)
Supplement: Additional file 4 — Results. A folder containing a complete version of the results we generated for this paper. (ZIP 1597 kb) [file 12859_2016_1229_MOESM4_ESM.zip › output/HGT50_any_Results/WBPaper00037950_AVA-neuron_embryo_enriched_WBbt_0005842_534.pdf]

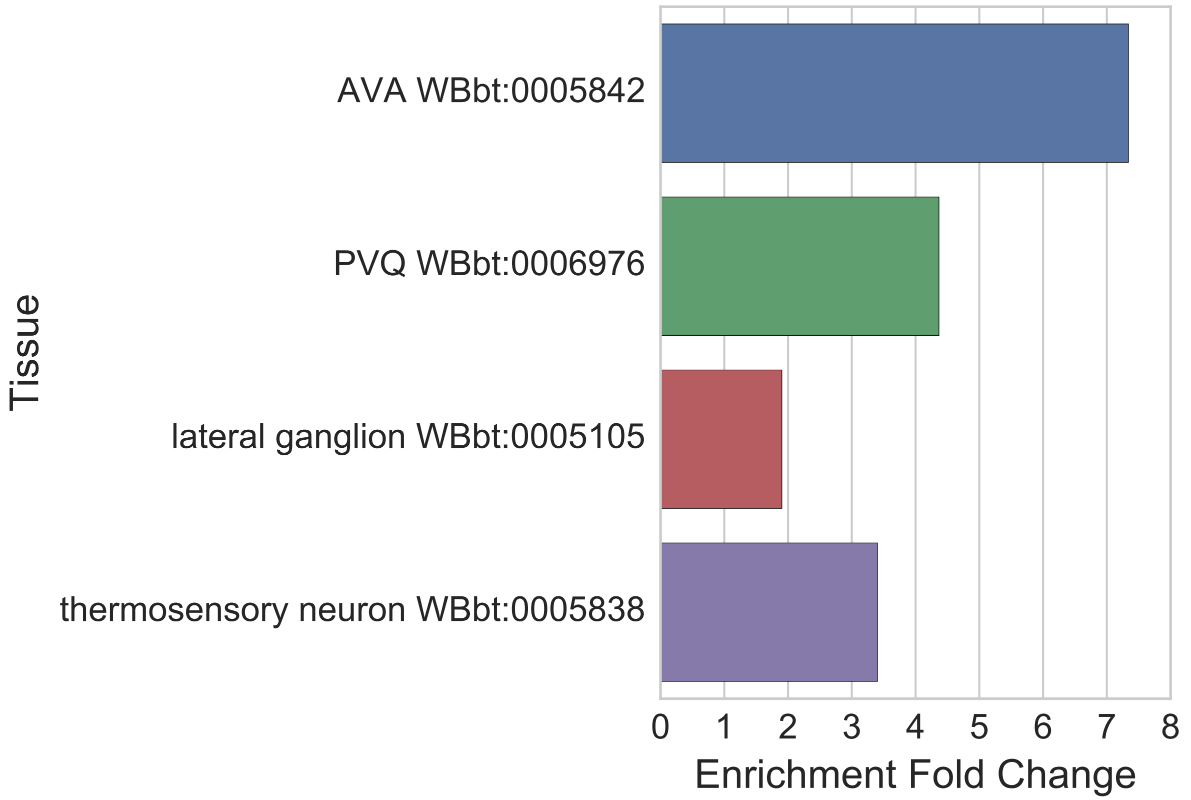
AVA WBbt:0005842
PVQ WBbt:0006976
lateral ganglion WBbt:0005105
thermosensory neuron WBbt:0005838
Tissue
0
1
2
3
4
5
6
7
8
Enrichment Fold Change
