# Supplementary figures and images for: Tissue enrichment analysis for C. elegans genomics
Source: BMC Bioinformatics. 2016 Sep 13;17(1):366. doi: 10.1186/s12859-016-1229-9 (PMC5020436; doi:10.1186/s12859-016-1229-9)

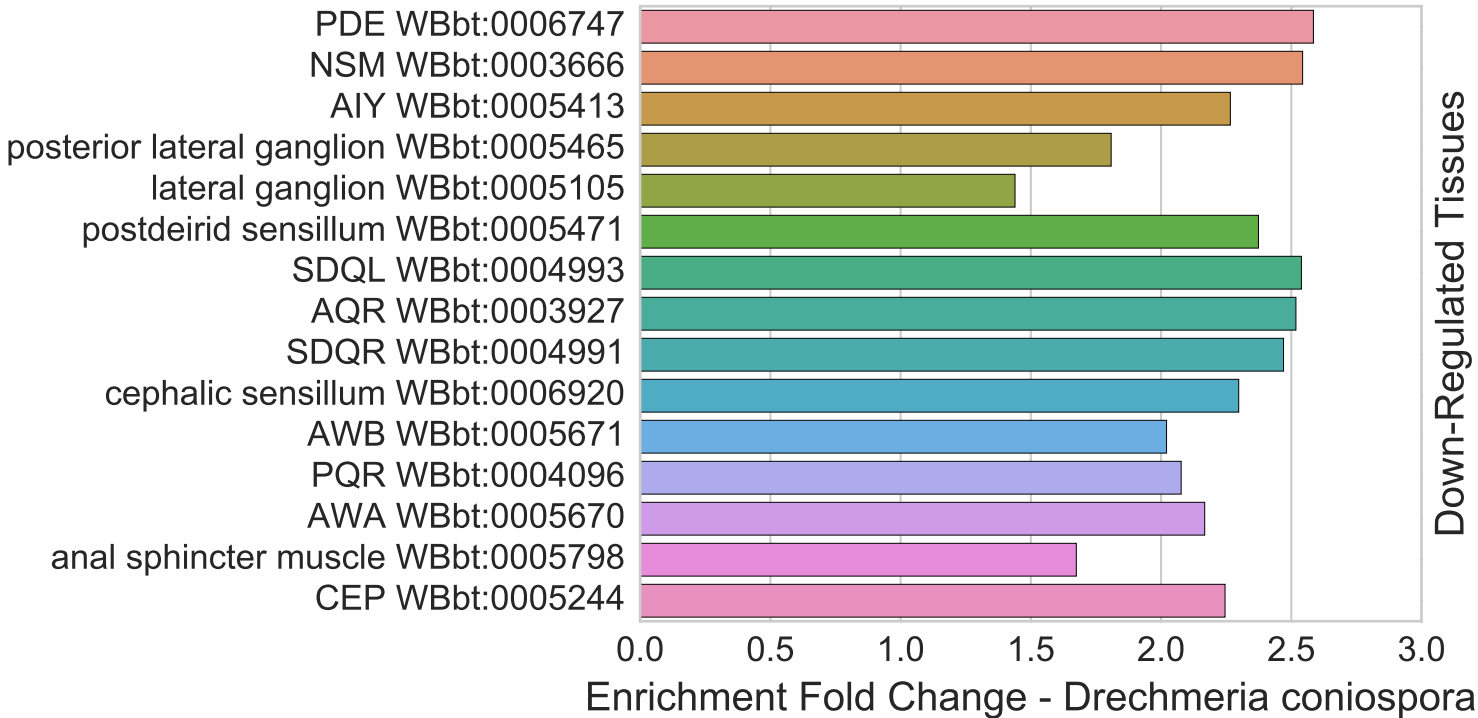

Supplement: Additional file 4 — Results. A folder containing a complete version of the results we generated for this paper. (ZIP 1597 kb) [file 12859_2016_1229_MOESM4_ESM.zip › output/Engelmann/Graphs/Drechmeria coniosporaEnrichment.pdf]

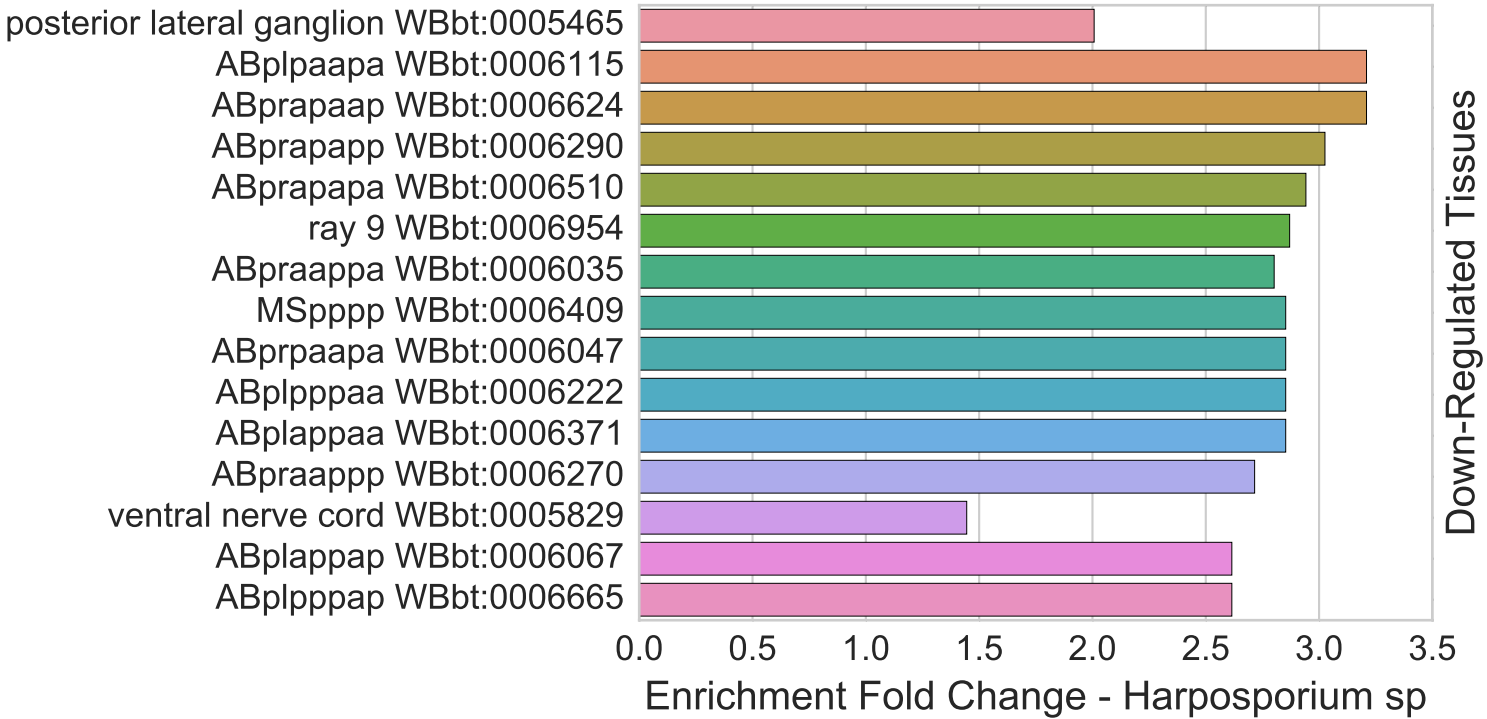

Supplement: Additional file 4 — Results. A folder containing a complete version of the results we generated for this paper. (ZIP 1597 kb) [file 12859_2016_1229_MOESM4_ESM.zip › output/Engelmann/Graphs/Harposporium spEnrichment.pdf]

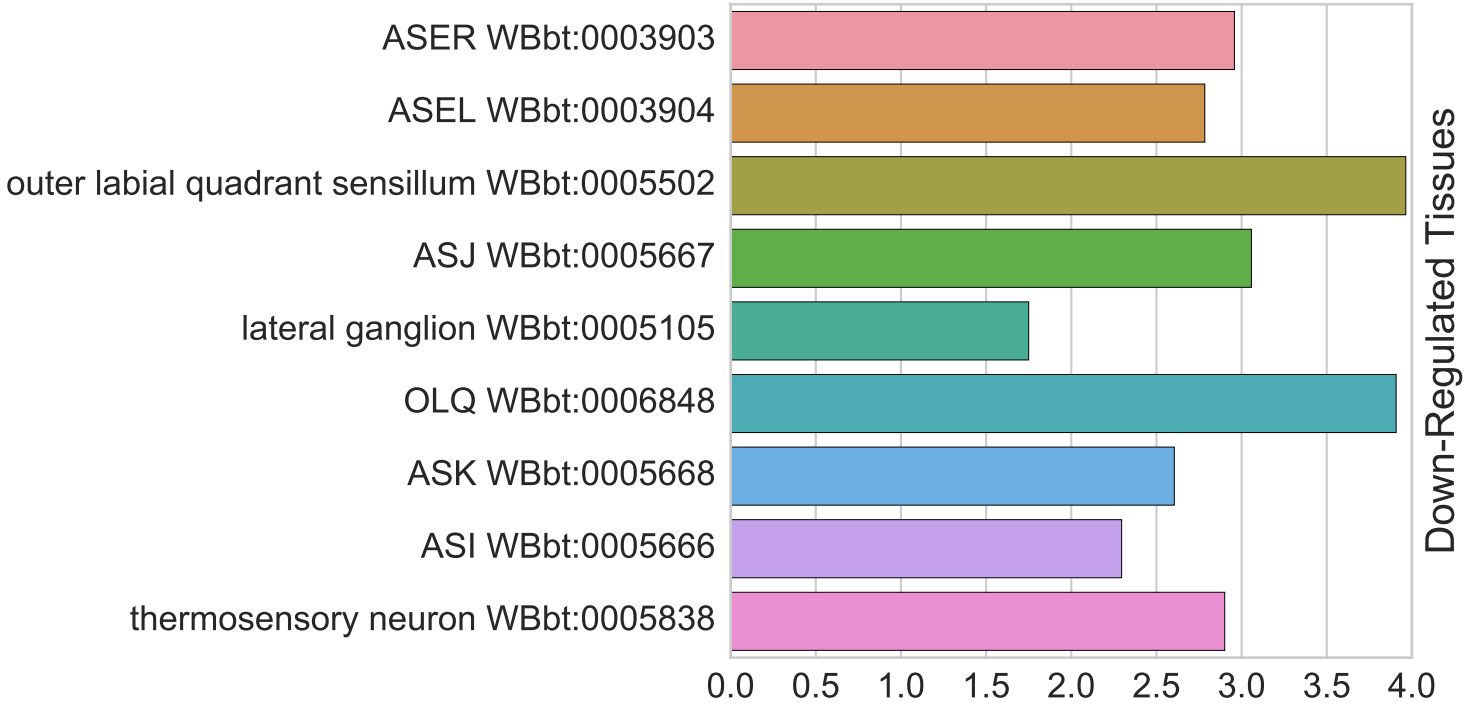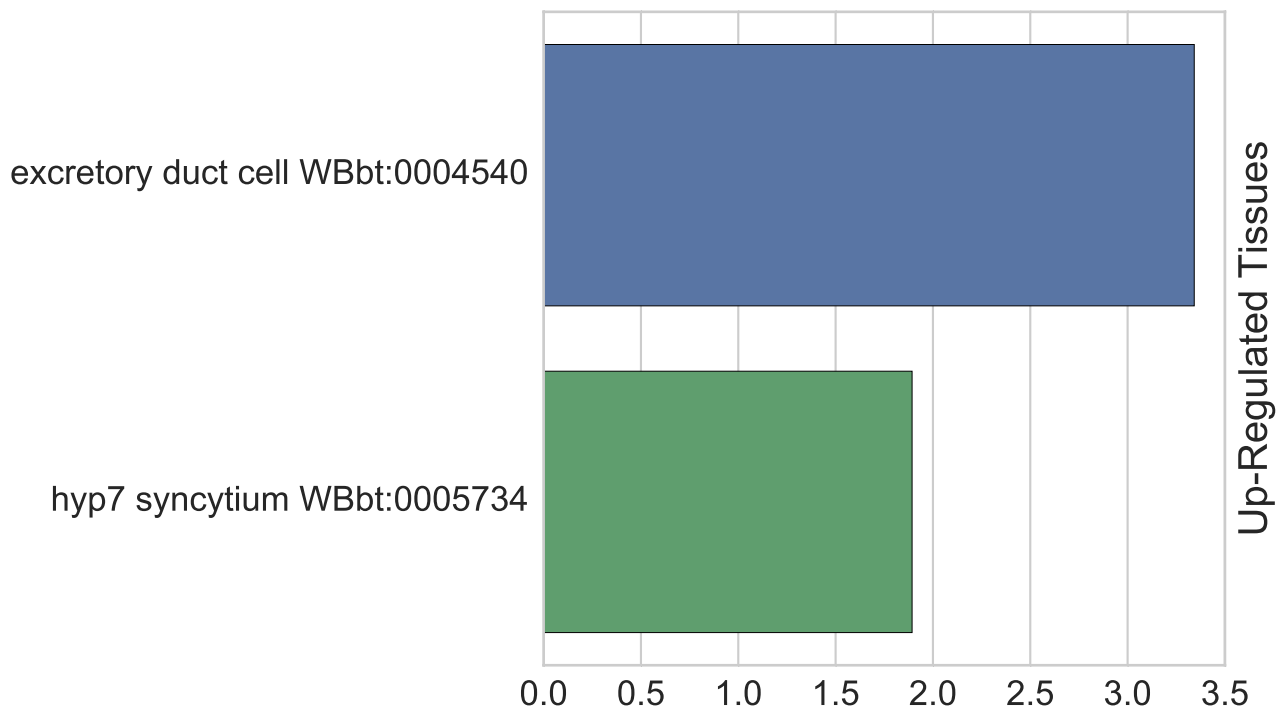

Enrichment Fold Change - *Serratia marcescens*

Supplement: Additional file 4 — Results. A folder containing a complete version of the results we generated for this paper. (ZIP 1597 kb) [file 12859_2016_1229_MOESM4_ESM.zip › output/Engelmann/Graphs/Serratia marcescensEnrichment.pdf]

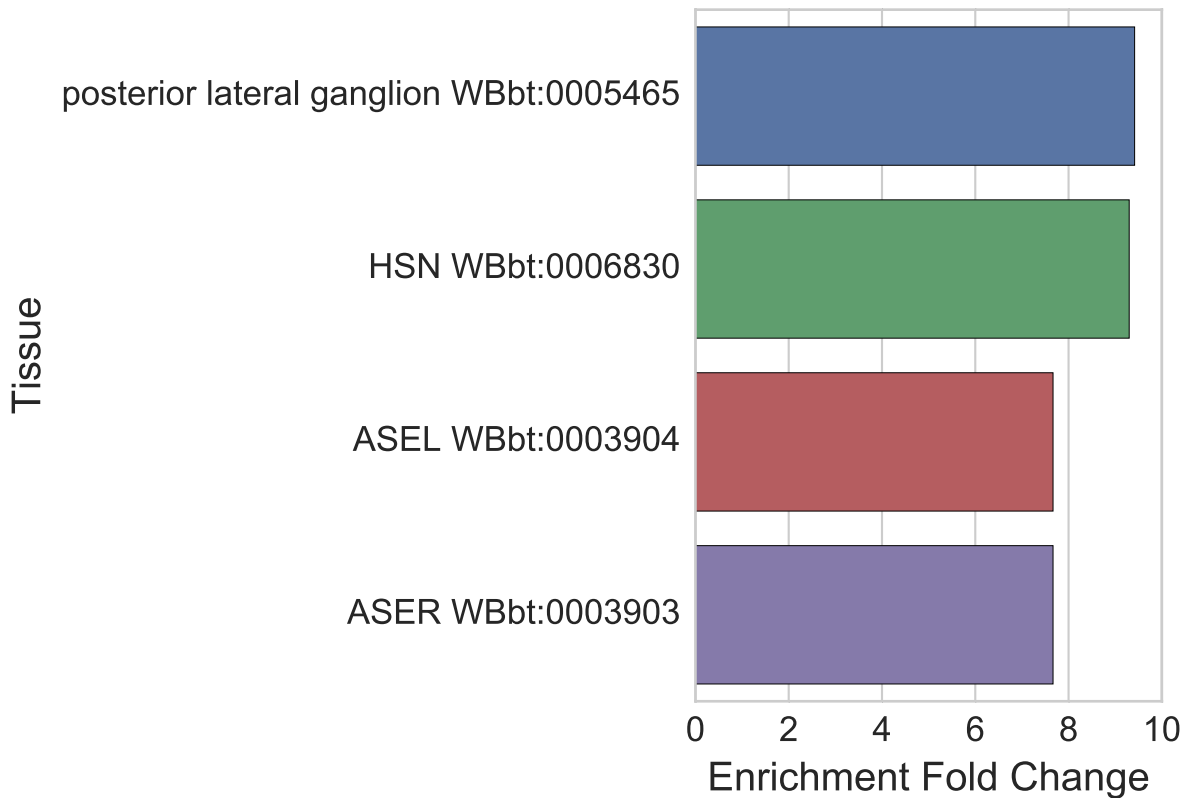

Supplement: Additional file 4 — Results. A folder containing a complete version of the results we generated for this paper. (ZIP 1597 kb) [file 12859_2016_1229_MOESM4_ESM.zip › output/HGT100_any_Results/WBPaper00013489_Ray_Enriched_WBbt_0006941_25.pdf]

Tissue

pharynx WBbt:0003681

0.0 0.2 0.4 0.6 0.8 1.0 1.2 1.4 1.6 1.8

Enrichment Fold Change

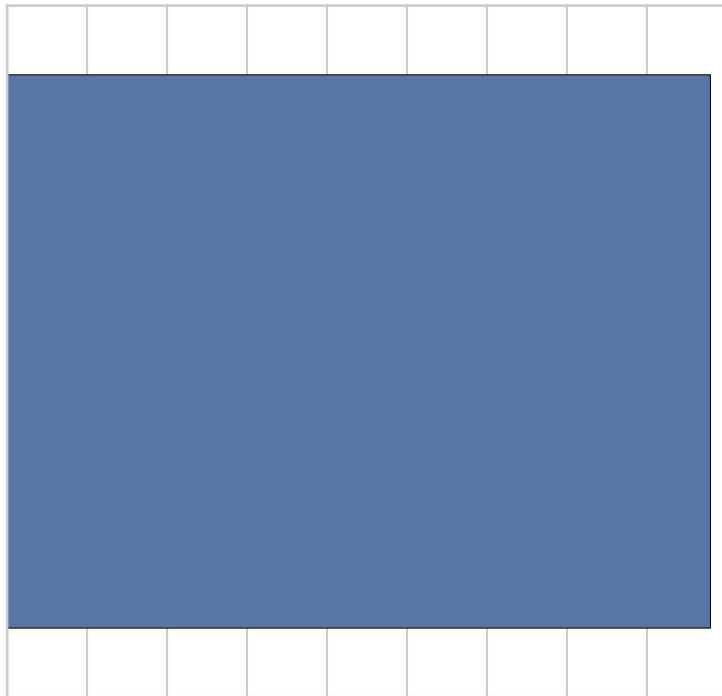

Supplement: Additional file 4 — Results. A folder containing a complete version of the results we generated for this paper. (ZIP 1597 kb) [file 12859_2016_1229_MOESM4_ESM.zip › output/HGT100_any_Results/WBPaper00024505_pharyngeal_enriched_WBbt_0003681_329.pdf]

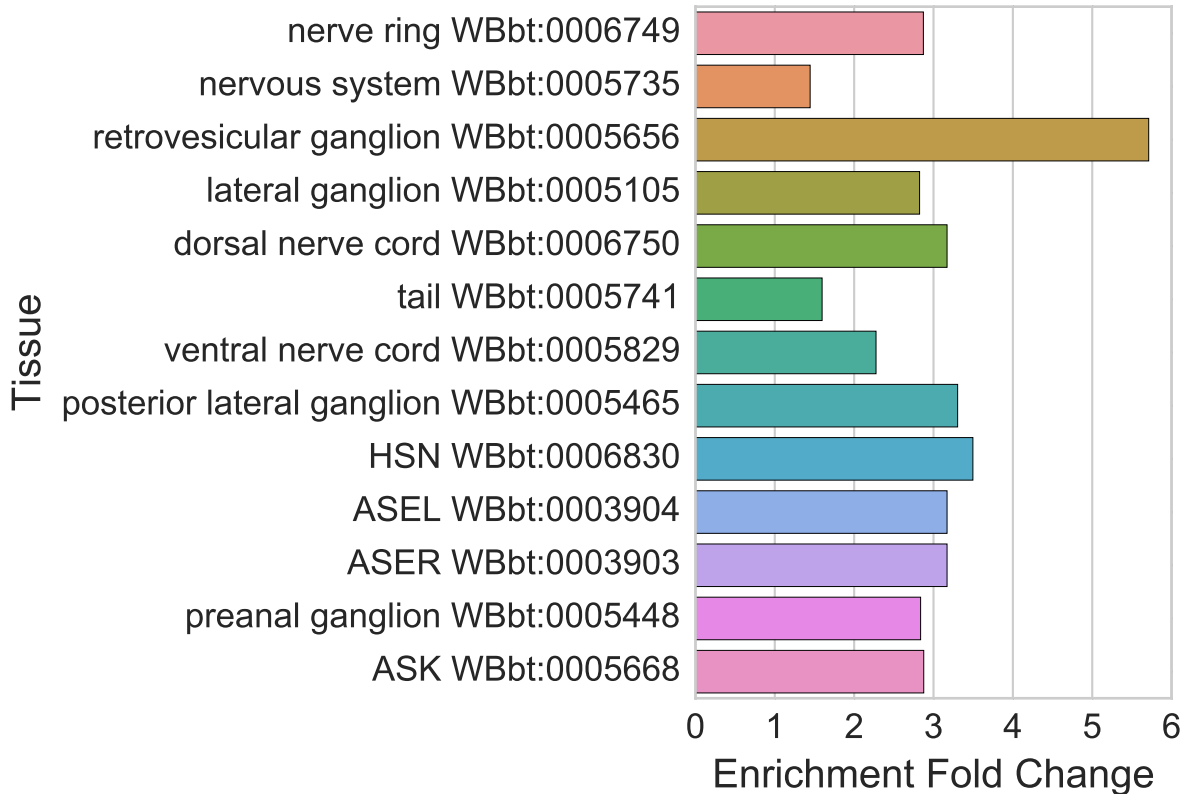

Supplement: Additional file 4 — Results. A folder containing a complete version of the results we generated for this paper. (ZIP 1597 kb) [file 12859_2016_1229_MOESM4_ESM.zip › output/HGT100_any_Results/WBPaper00024970_GABAergic_neuron_specific_WBbt_0005190_247.pdf]

Tissue

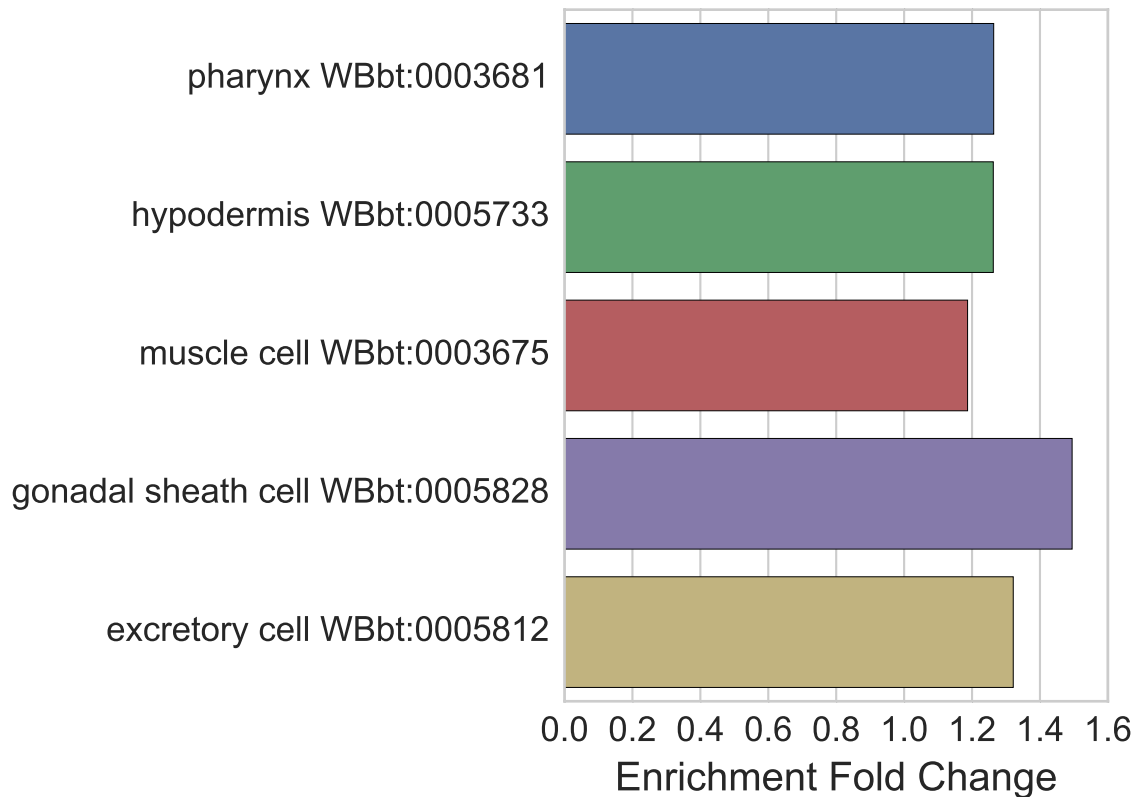

Supplement: Additional file 4 — Results. A folder containing a complete version of the results we generated for this paper. (ZIP 1597 kb) [file 12859_2016_1229_MOESM4_ESM.zip › output/HGT100_any_Results/WBPaper00026980_intestine_enriched_WBbt_0005772_1970.pdf]

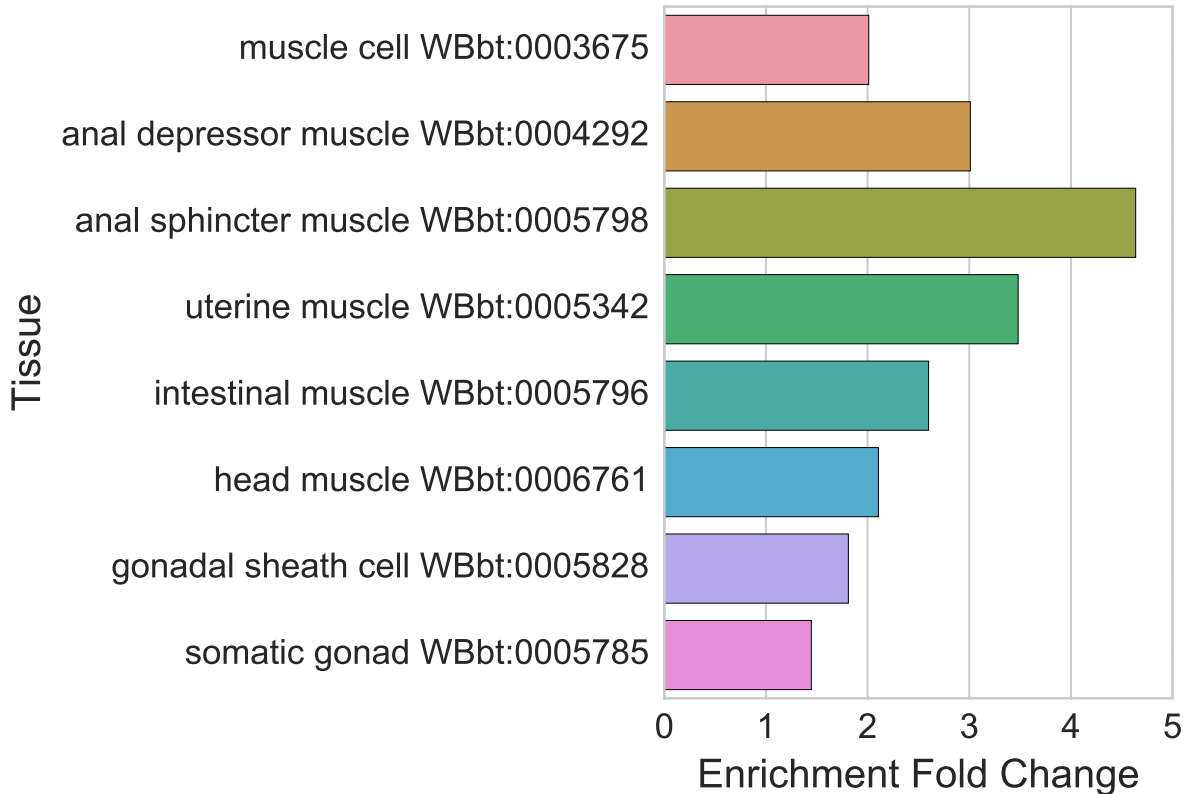

Supplement: Additional file 4 — Results. A folder containing a complete version of the results we generated for this paper. (ZIP 1597 kb) [file 12859_2016_1229_MOESM4_ESM.zip › output/HGT100_any_Results/WBPaper00031003_0hr_muscle_enriched_WBbt_0003675_761.pdf]

Tissue

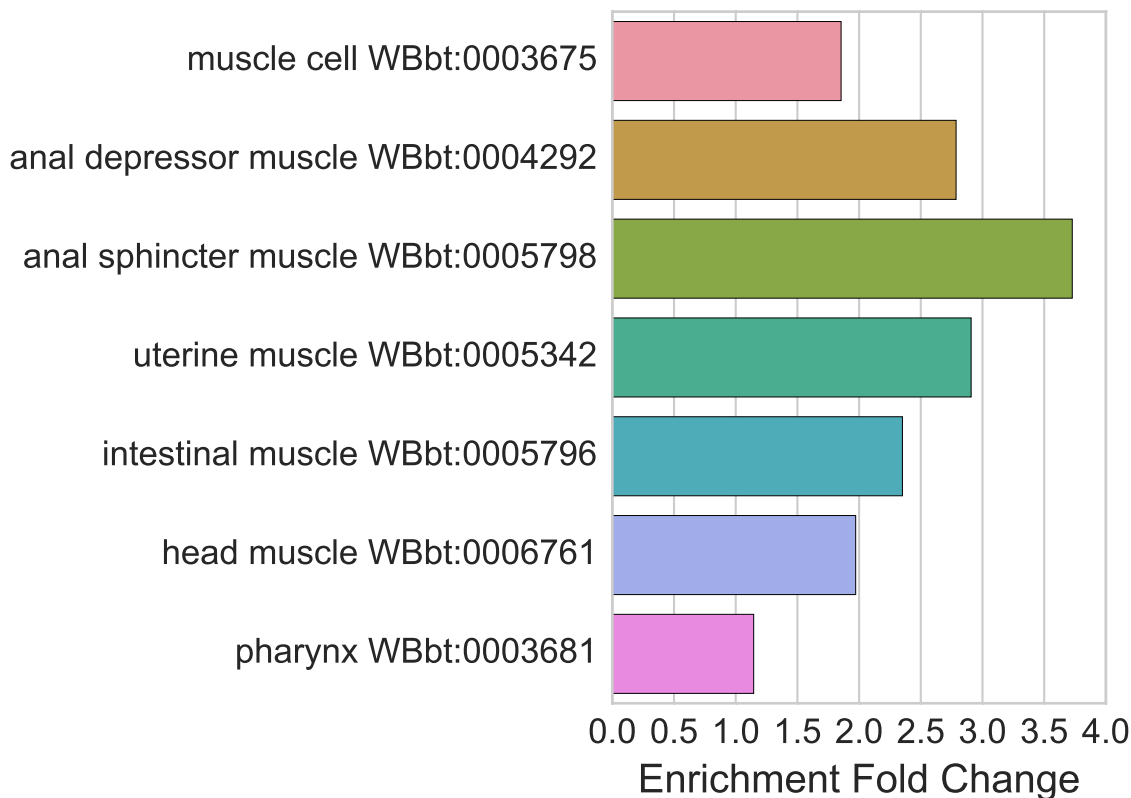

Supplement: Additional file 4 — Results. A folder containing a complete version of the results we generated for this paper. (ZIP 1597 kb) [file 12859_2016_1229_MOESM4_ESM.zip › output/HGT100_any_Results/WBPaper00031003_total_muscle_enriched_WBbt_0003675_1285.pdf]

Tissue

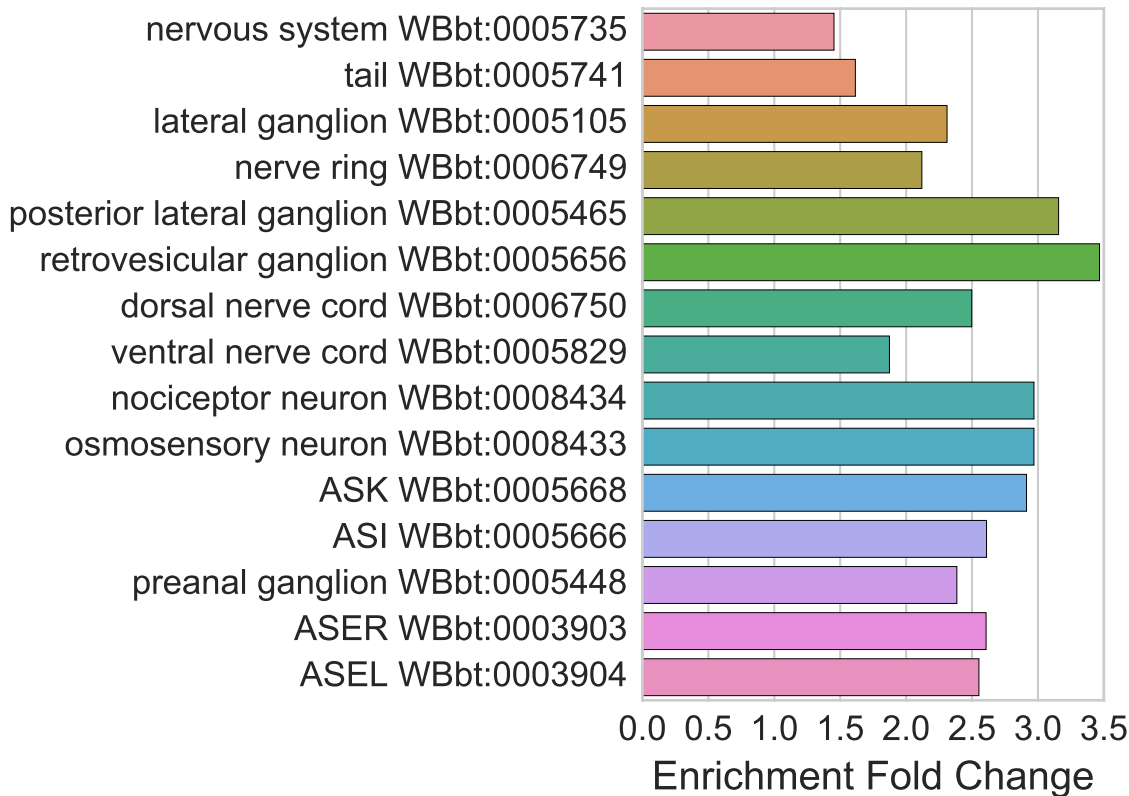

Supplement: Additional file 4 — Results. A folder containing a complete version of the results we generated for this paper. (ZIP 1597 kb) [file 12859_2016_1229_MOESM4_ESM.zip › output/HGT100_any_Results/WBPaper00031532_Larva_Pan_Neuronal_Enriched_WBbt_0003679_1603.pdf]

Tissue

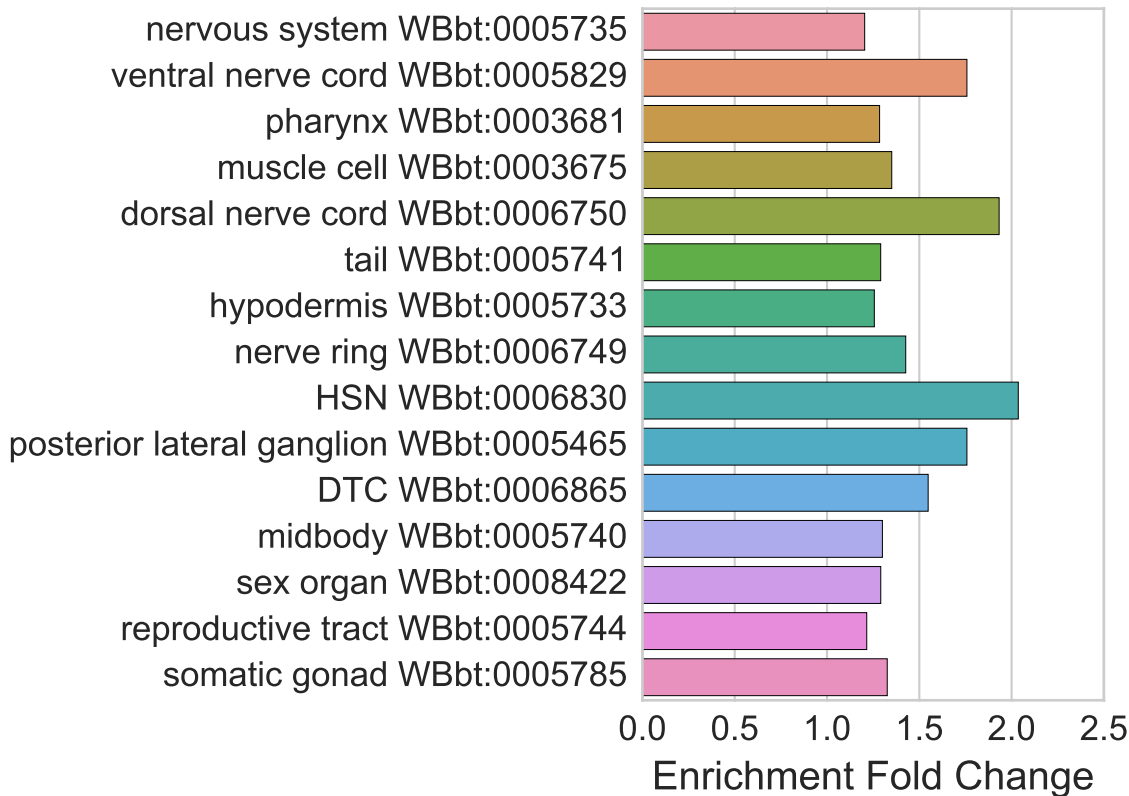

Supplement: Additional file 4 — Results. A folder containing a complete version of the results we generated for this paper. (ZIP 1597 kb) [file 12859_2016_1229_MOESM4_ESM.zip › output/HGT100_any_Results/WBPaper00036375_enriched_in_PVD_OLL_WBbt_0006831_2180.pdf]

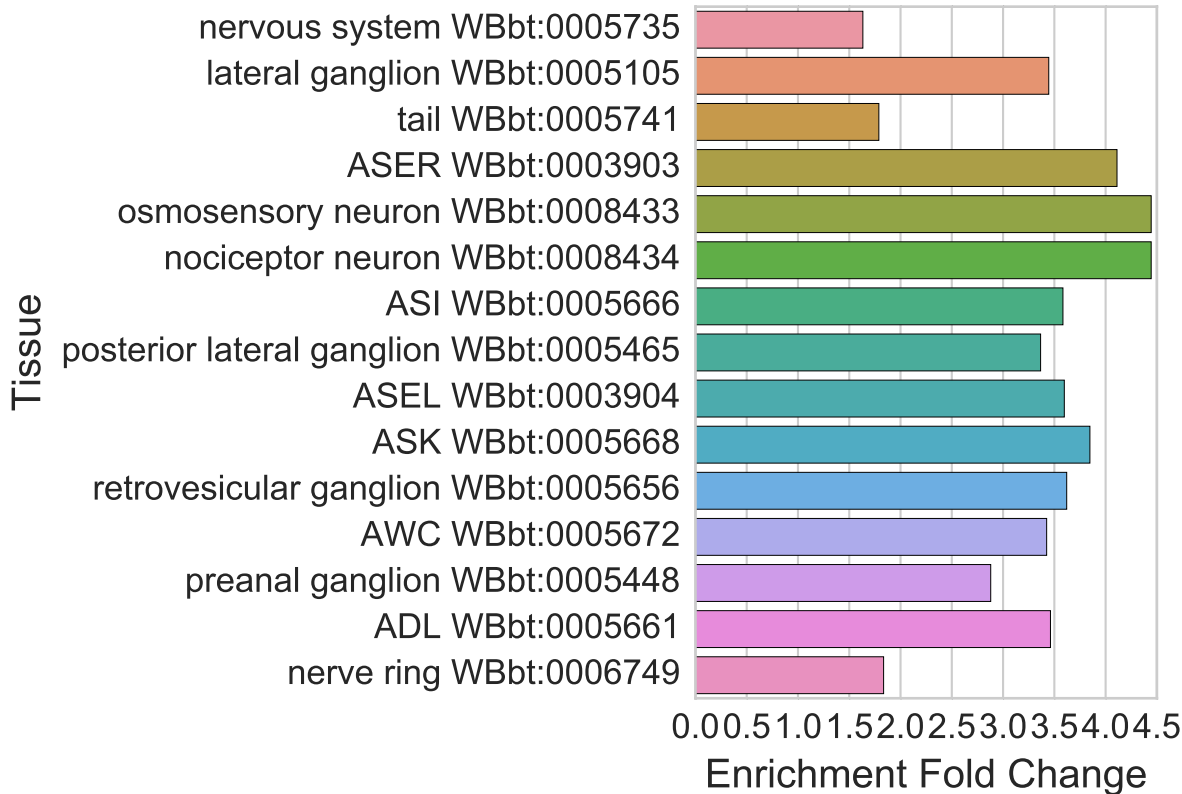

Supplement: Additional file 4 — Results. A folder containing a complete version of the results we generated for this paper. (ZIP 1597 kb) [file 12859_2016_1229_MOESM4_ESM.zip › output/HGT100_any_Results/WBPaper00037950_all-neurons_larva_enriched_WBbt_0003679_1013.pdf]

Tissue

lateral ganglion WBbt:0005105

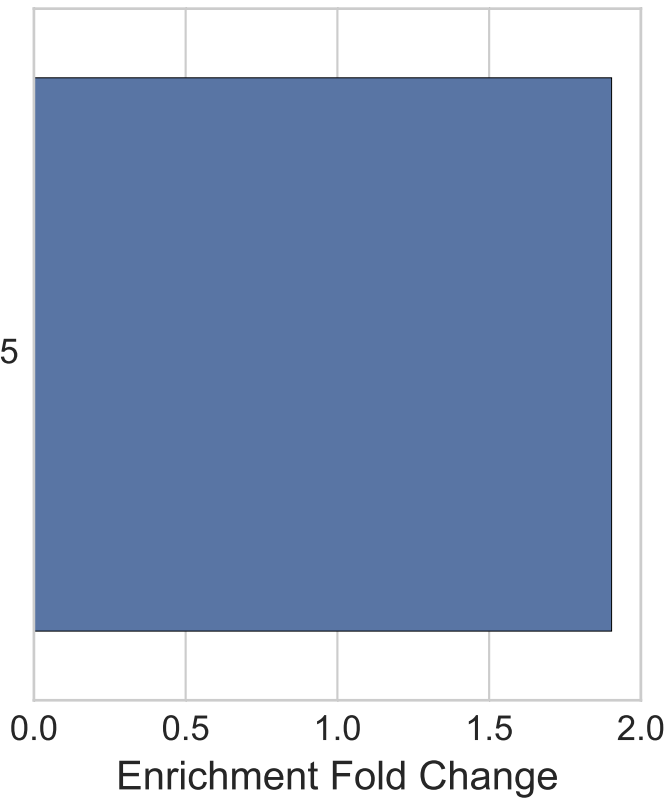

Supplement: Additional file 4 — Results. A folder containing a complete version of the results we generated for this paper. (ZIP 1597 kb) [file 12859_2016_1229_MOESM4_ESM.zip › output/HGT100_any_Results/WBPaper00037950_AVA-neuron_embryo_enriched_WBbt_0005842_534.pdf]

Tissue

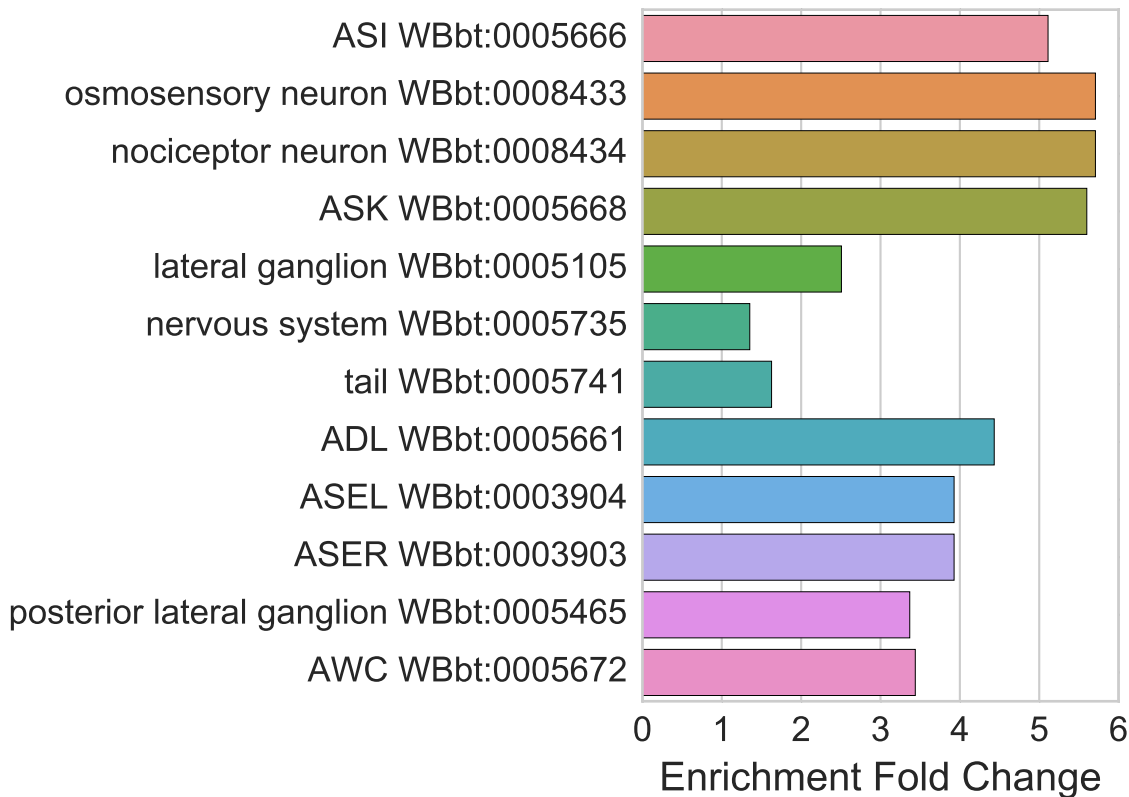

Supplement: Additional file 4 — Results. A folder containing a complete version of the results we generated for this paper. (ZIP 1597 kb) [file 12859_2016_1229_MOESM4_ESM.zip › output/HGT100_any_Results/WBPaper00037950_BAG-neuron_embryo_enriched_WBbt_0006825_454.pdf]

Tissue

coelomic system WBbt:0005749

pharynx WBbt:0003681

0.0 0.5 1.0 1.5 2.0 2.5 3.0 3.5 4.0 4.5

Enrichment Fold Change

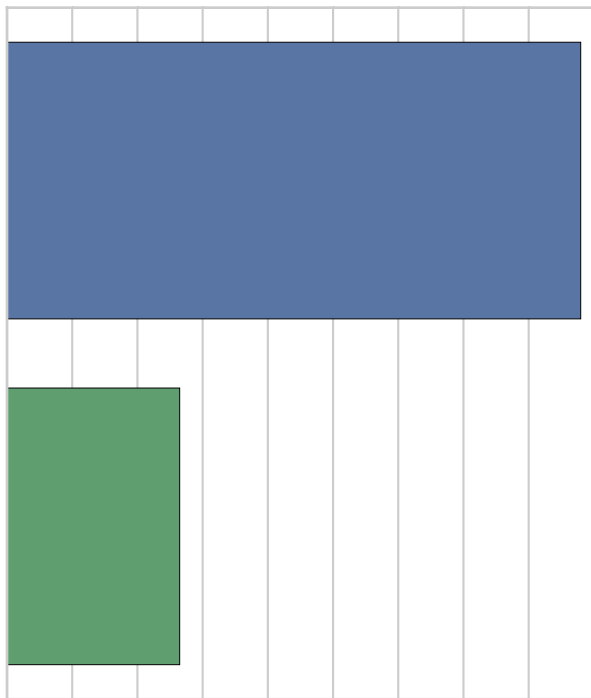

Supplement: Additional file 4 — Results. A folder containing a complete version of the results we generated for this paper. (ZIP 1597 kb) [file 12859_2016_1229_MOESM4_ESM.zip › output/HGT100_any_Results/WBPaper00037950_coelomocytes_embryo_enriched_WBbt_0005751_570.pdf]

Tissue

coelomic system WBbt:0005749

0

1

2

3

4

5

Enrichment Fold Change

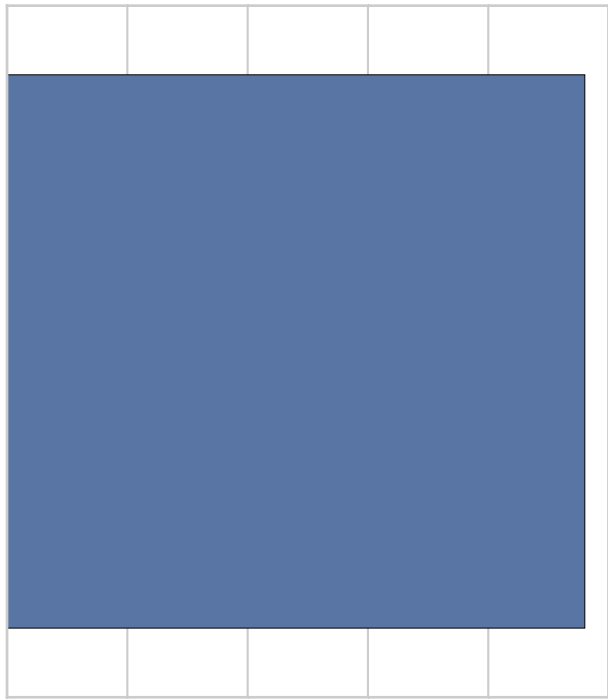

Supplement: Additional file 4 — Results. A folder containing a complete version of the results we generated for this paper. (ZIP 1597 kb) [file 12859_2016_1229_MOESM4_ESM.zip › output/HGT100_any_Results/WBPaper00037950_coelomocytes_larva_enriched_WBbt_0005751_229.pdf]

Tissue

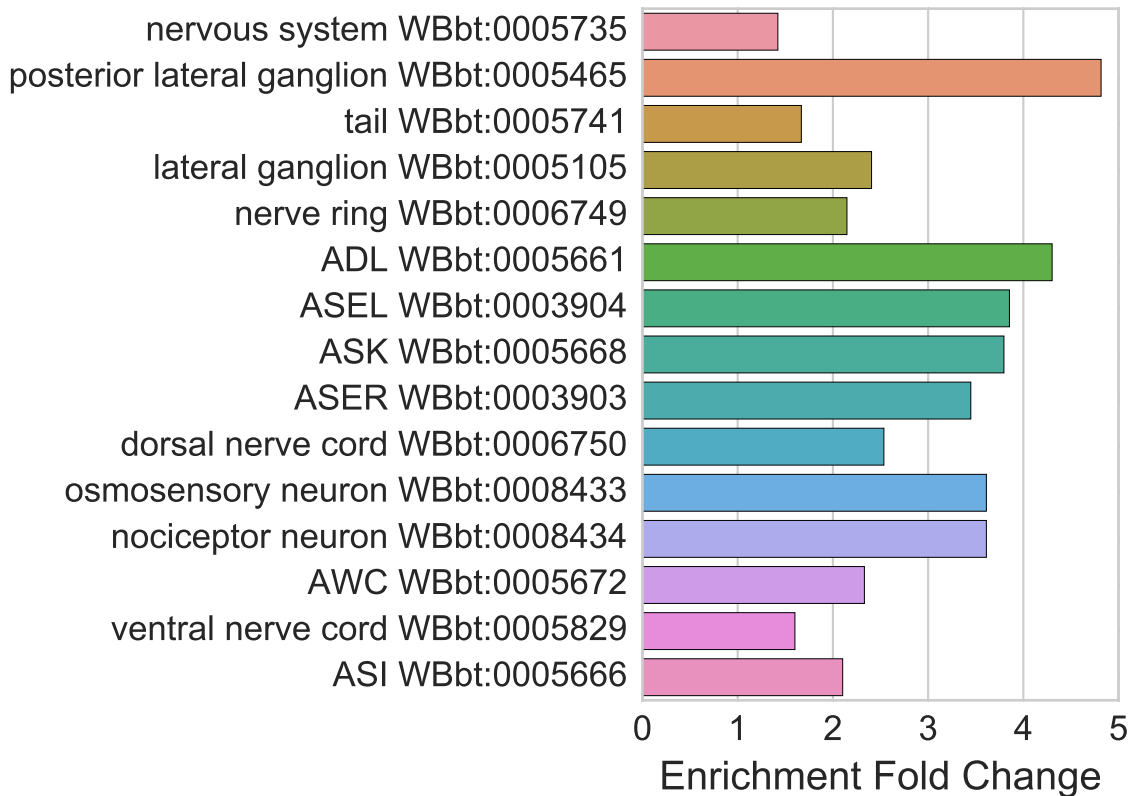

Supplement: Additional file 4 — Results. A folder containing a complete version of the results we generated for this paper. (ZIP 1597 kb) [file 12859_2016_1229_MOESM4_ESM.zip › output/HGT100_any_Results/WBPaper00037950_dopaminergic-neurons_embryo_enriched_WBbt_0006746_466.pdf]

Tissue

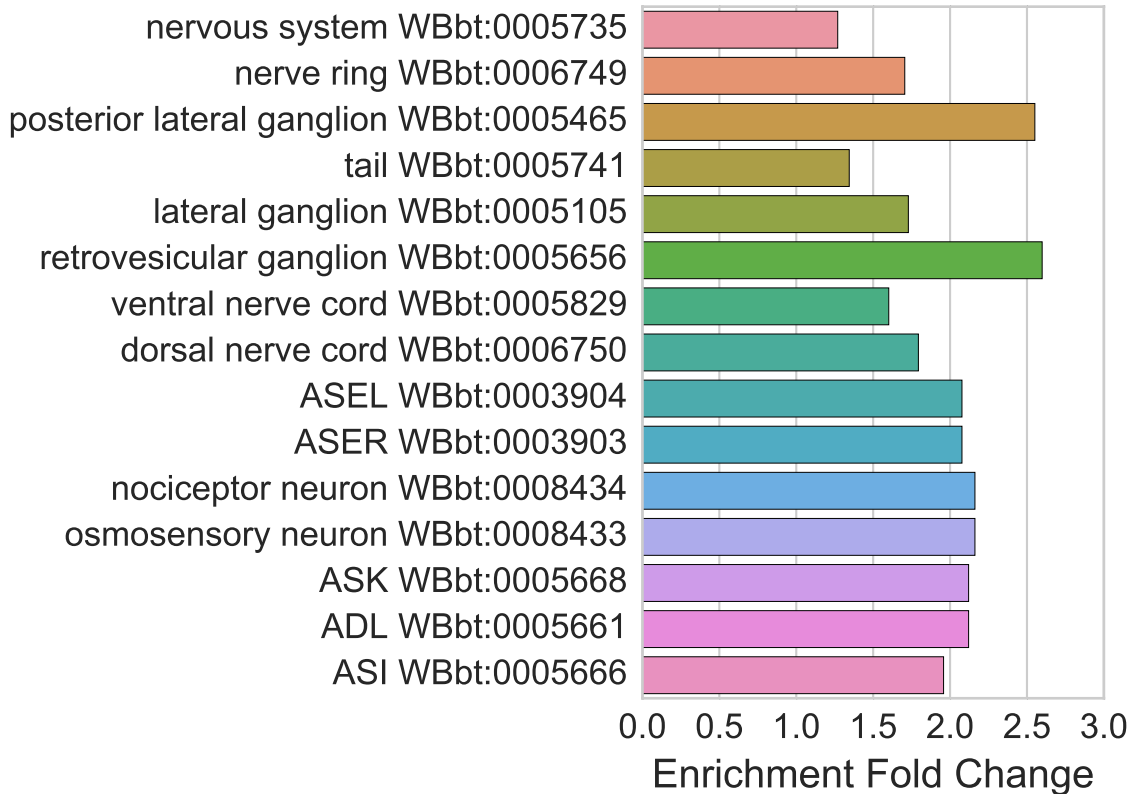

Supplement: Additional file 4 — Results. A folder containing a complete version of the results we generated for this paper. (ZIP 1597 kb) [file 12859_2016_1229_MOESM4_ESM.zip › output/HGT100_any_Results/WBPaper00037950_dopaminergic-neurons_larva_enriched_WBbt_0006746_1230.pdf]

Tissue

excretory cell WBbt:0005812

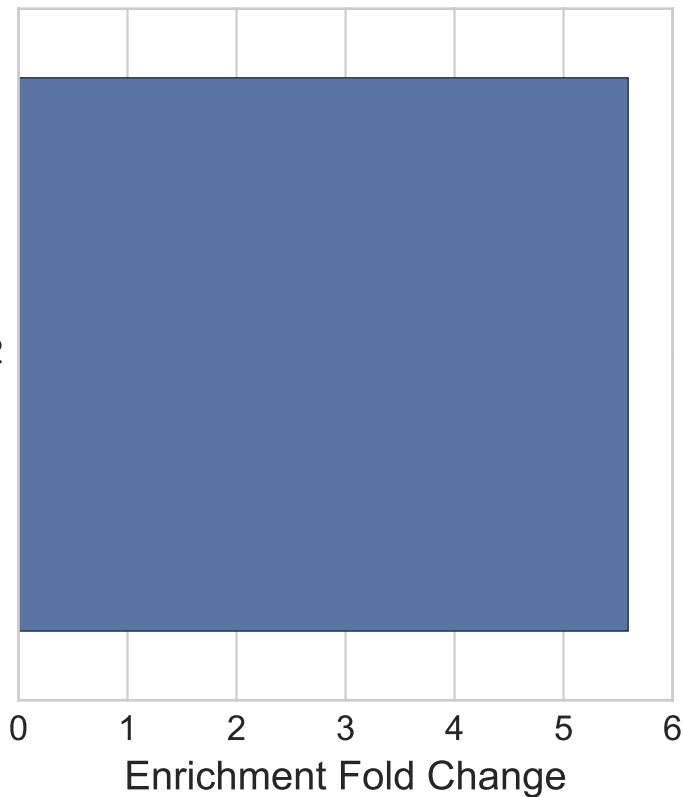

Supplement: Additional file 4 — Results. A folder containing a complete version of the results we generated for this paper. (ZIP 1597 kb) [file 12859_2016_1229_MOESM4_ESM.zip › output/HGT100_any_Results/WBPaper00037950_excretory-cell_larva_enriched_WBbt_0005812_528.pdf]

Tissue

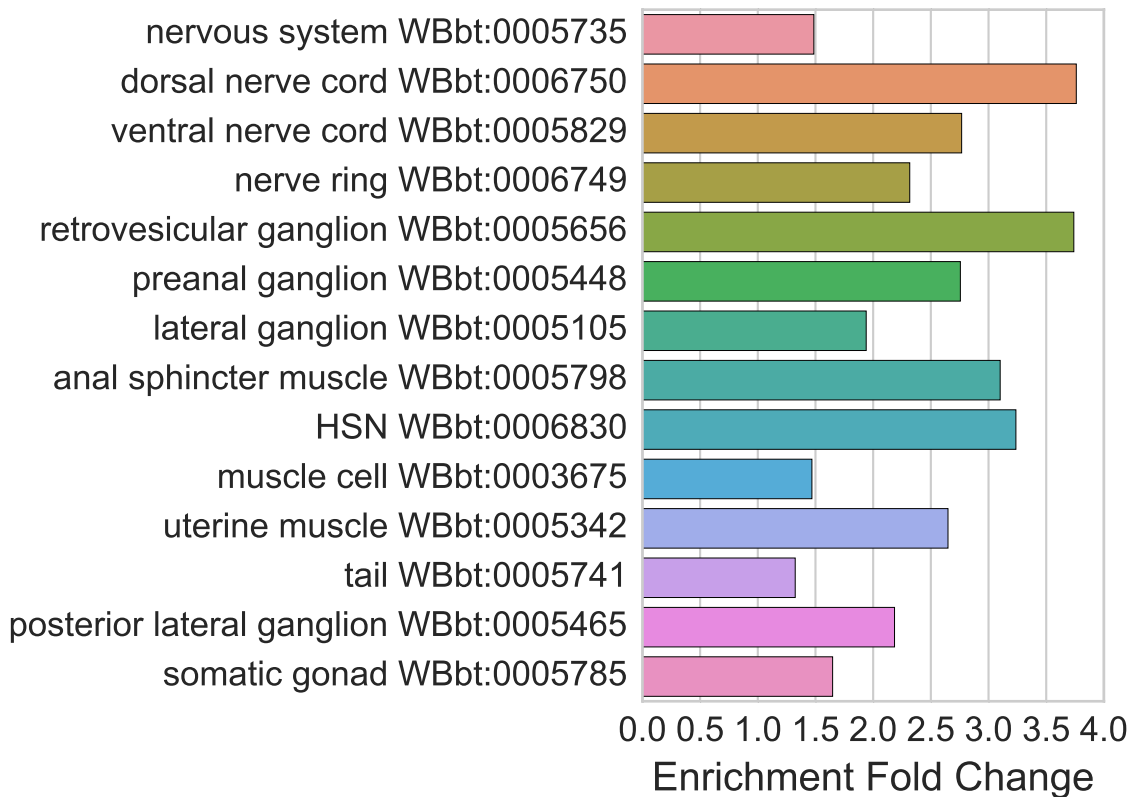

Supplement: Additional file 4 — Results. A folder containing a complete version of the results we generated for this paper. (ZIP 1597 kb) [file 12859_2016_1229_MOESM4_ESM.zip › output/HGT100_any_Results/WBPaper00037950_GABAergic-motor-neurons_embryo_enriched_WBbt_0005190_361.pdf]

Tissue

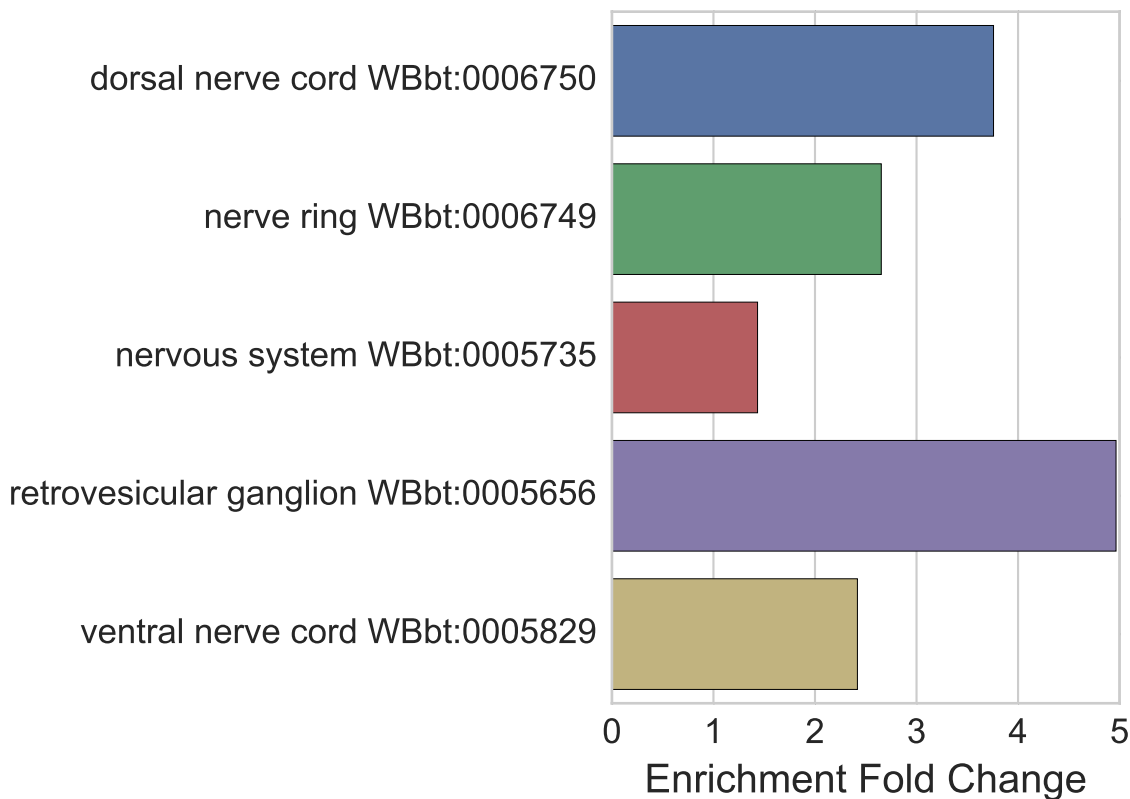

Supplement: Additional file 4 — Results. A folder containing a complete version of the results we generated for this paper. (ZIP 1597 kb) [file 12859_2016_1229_MOESM4_ESM.zip › output/HGT100_any_Results/WBPaper00037950_GABAergic-motor-neurons_larva_enriched_WBbt_0005190_132.pdf]

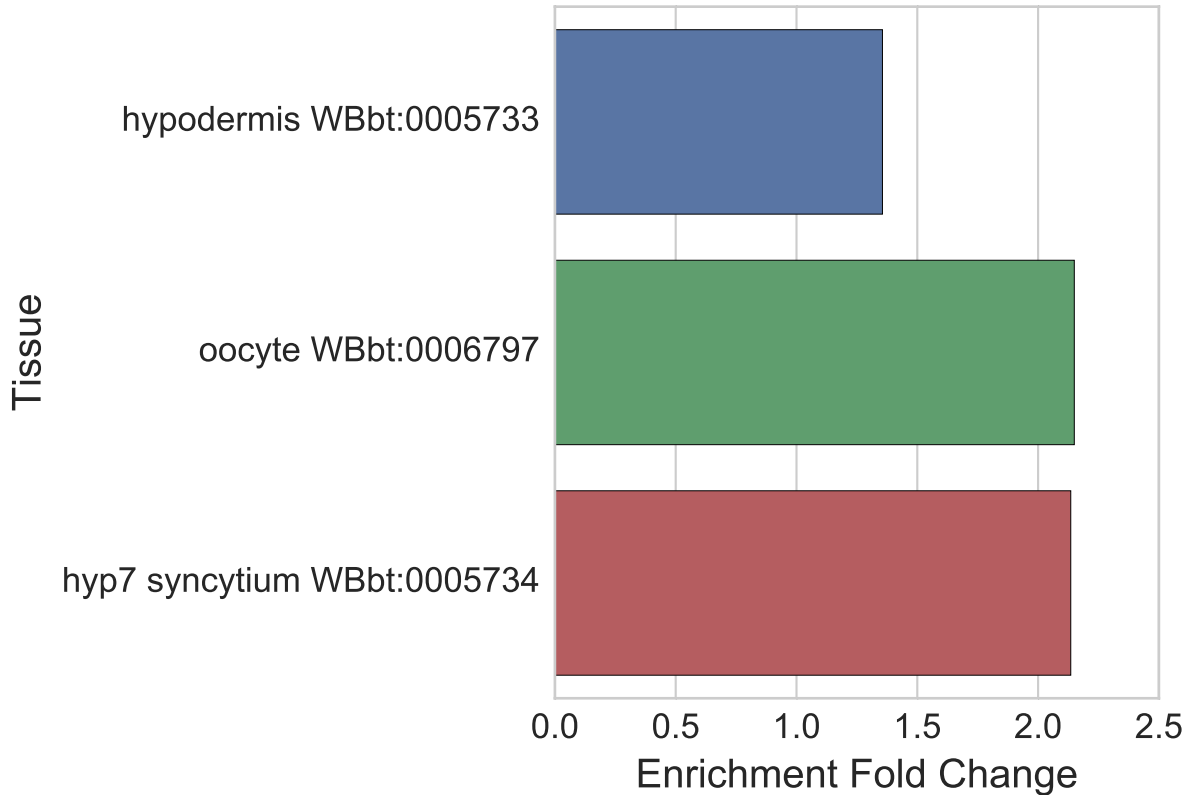

Supplement: Additional file 4 — Results. A folder containing a complete version of the results we generated for this paper. (ZIP 1597 kb) [file 12859_2016_1229_MOESM4_ESM.zip › output/HGT100_any_Results/WBPaper00037950_germline-precursors_embryo_enriched_WBbt_0006849_974.pdf]

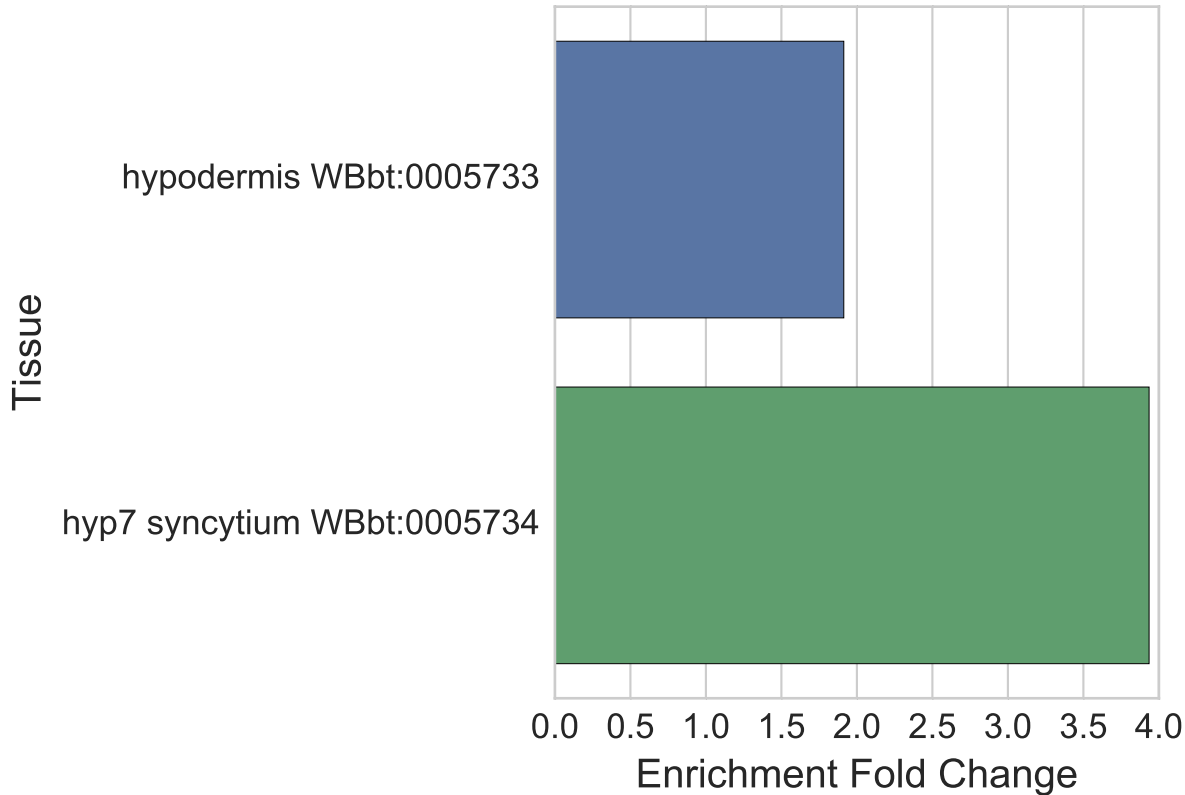

Supplement: Additional file 4 — Results. A folder containing a complete version of the results we generated for this paper. (ZIP 1597 kb) [file 12859_2016_1229_MOESM4_ESM.zip › output/HGT100_any_Results/WBPaper00037950_hypodermis_embryo_enriched_WBbt_0005733_734.pdf]

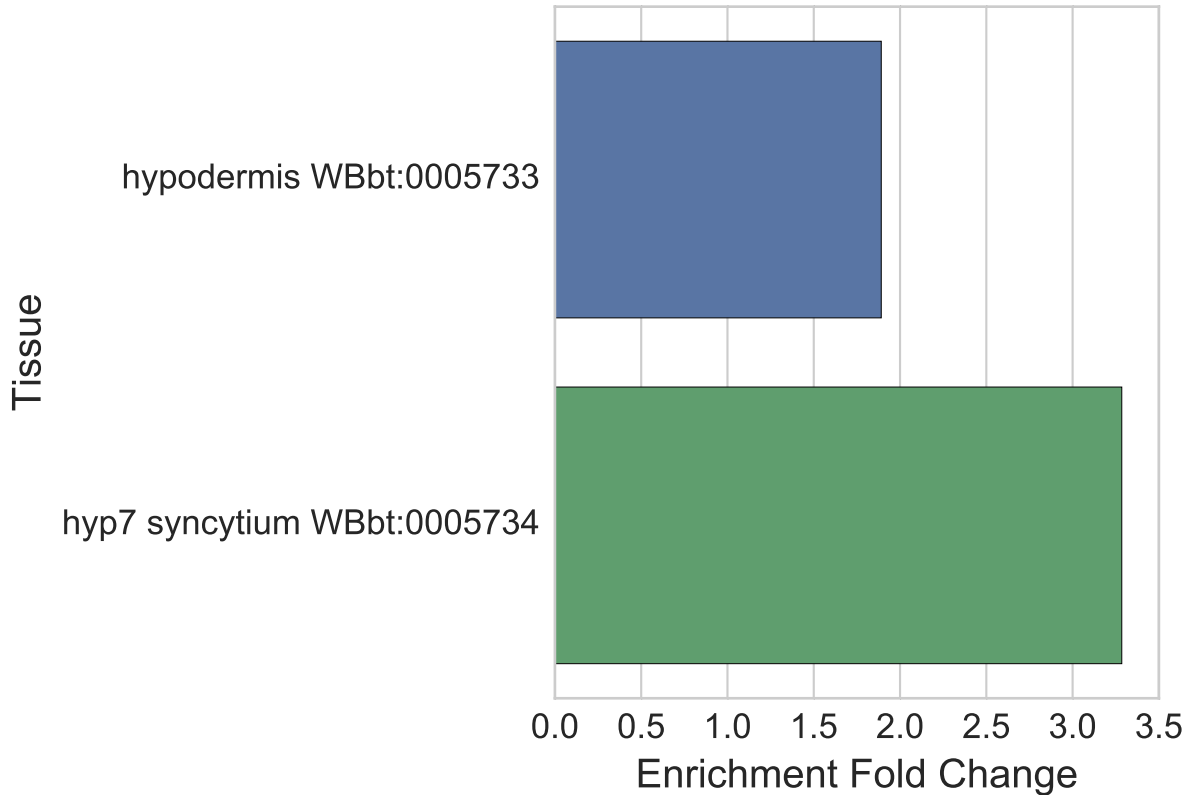

Supplement: Additional file 4 — Results. A folder containing a complete version of the results we generated for this paper. (ZIP 1597 kb) [file 12859_2016_1229_MOESM4_ESM.zip › output/HGT100_any_Results/WBPaper00037950_hypodermis_larva_enriched_WBbt_0005733_1250.pdf]

Tissue

nerve ring WBbt:0006749

0.0 0.2 0.4 0.6 0.8 1.0 1.2 1.4 1.6

Enrichment Fold Change

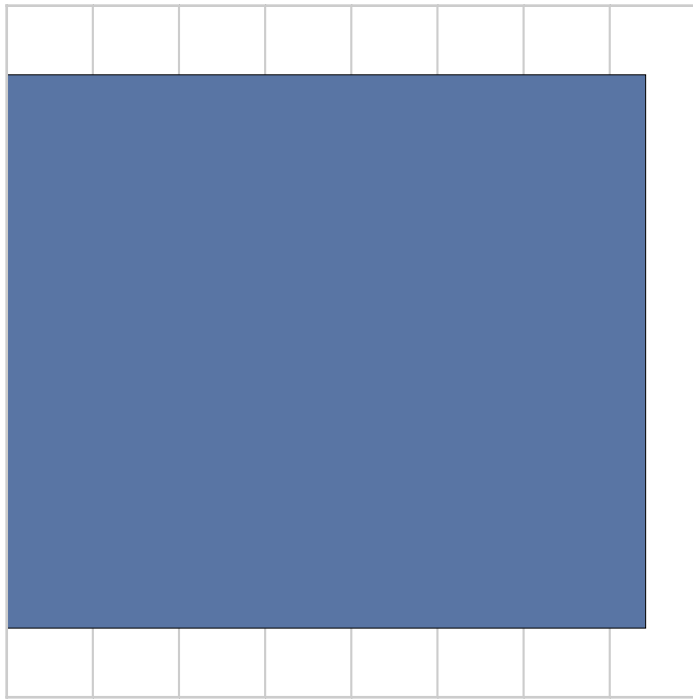

Supplement: Additional file 4 — Results. A folder containing a complete version of the results we generated for this paper. (ZIP 1597 kb) [file 12859_2016_1229_MOESM4_ESM.zip › output/HGT100_any_Results/WBPaper00037950_PVD-OLL-neurons_larva_enriched_WBbt_0006831_878.pdf]

Tissue

posterior lateral ganglion WBbt:0005465

tail WBbt:0005741

nervous system WBbt:0005735

0 1 2 3 4 5 6 7  
Enrichment Fold Change

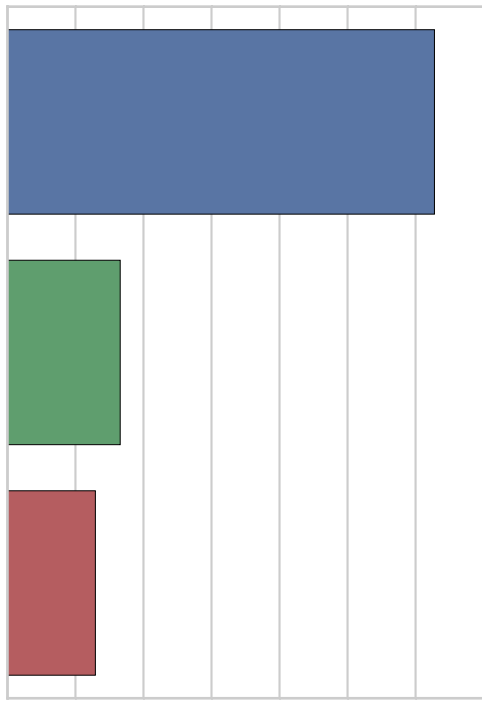

Supplement: Additional file 4 — Results. A folder containing a complete version of the results we generated for this paper. (ZIP 1597 kb) [file 12859_2016_1229_MOESM4_ESM.zip › output/HGT100_any_Results/WBPaper00040420_ALM_PLM_enriched_WBbt_0005406_198.pdf]

Tissue

posterior lateral ganglion WBbt:0005465

tail WBbt:0005741

nervous system WBbt:0005735

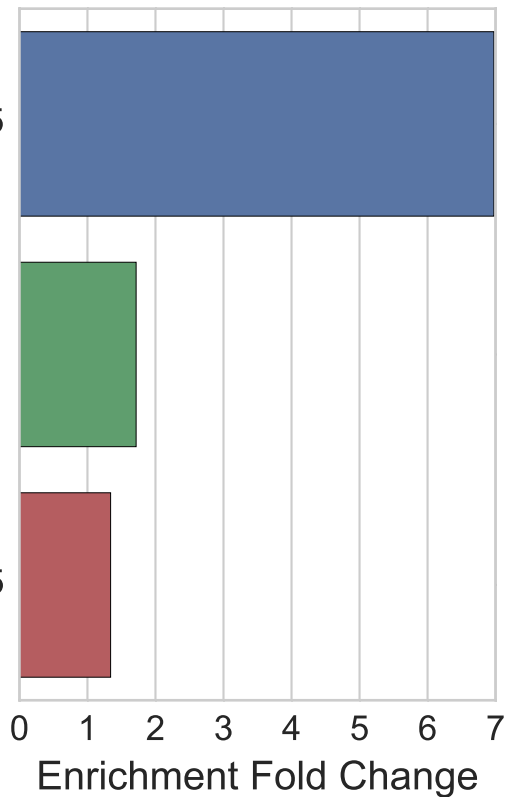

Supplement: Additional file 4 — Results. A folder containing a complete version of the results we generated for this paper. (ZIP 1597 kb) [file 12859_2016_1229_MOESM4_ESM.zip › output/HGT100_any_Results/WBPaper00040420_FLP_enriched_WBbt_0006828_288.pdf]

Tissue

oocyte WBbt:0006797

0.0

0.5

1.0

1.5

2.0

2.5

3.0

Enrichment Fold Change

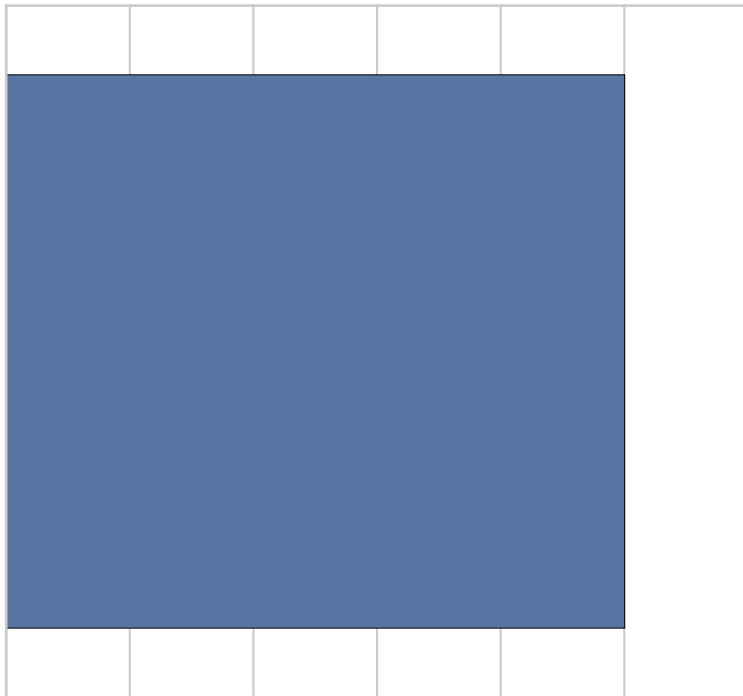

Supplement: Additional file 4 — Results. A folder containing a complete version of the results we generated for this paper. (ZIP 1597 kb) [file 12859_2016_1229_MOESM4_ESM.zip › output/HGT100_any_Results/WBPaper00044760_germline_specific_WBbt_0005784_2510.pdf]

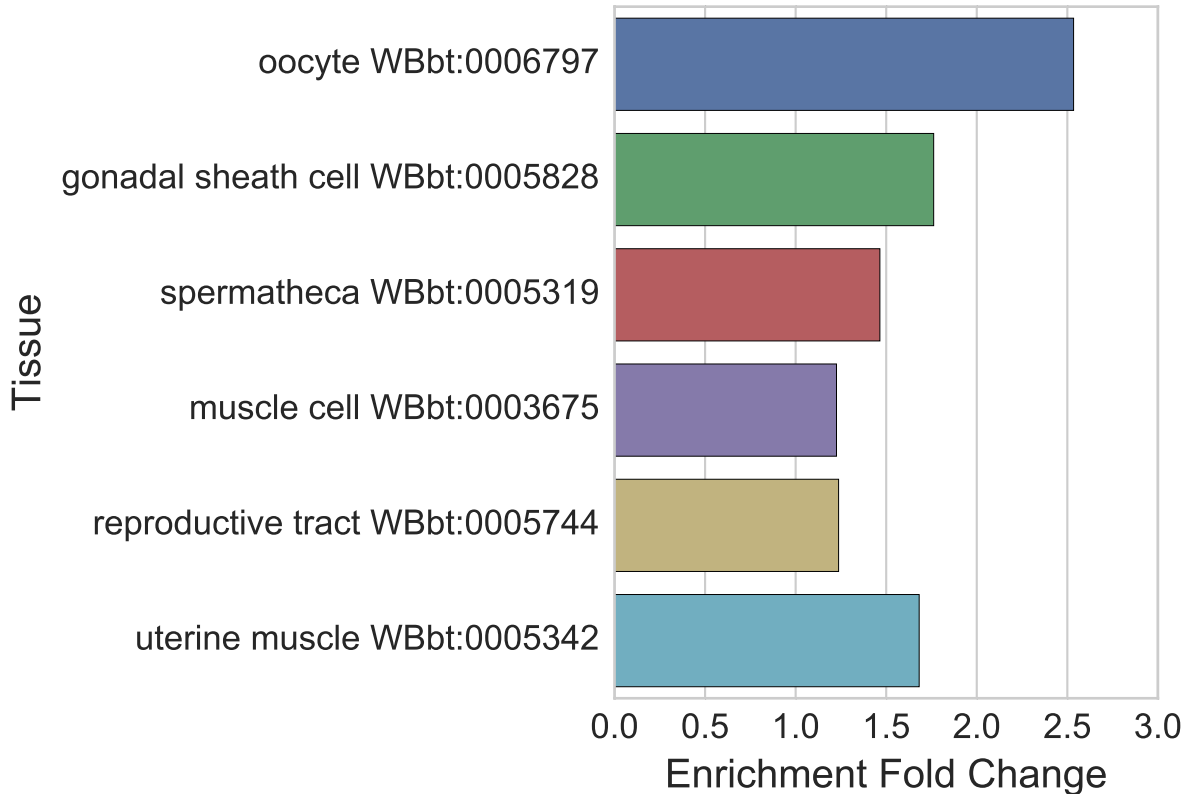

Supplement: Additional file 4 — Results. A folder containing a complete version of the results we generated for this paper. (ZIP 1597 kb) [file 12859_2016_1229_MOESM4_ESM.zip › output/HGT100_any_Results/WBPaper00045521_Spermatogenic_WBbt_0005784_2743.pdf]

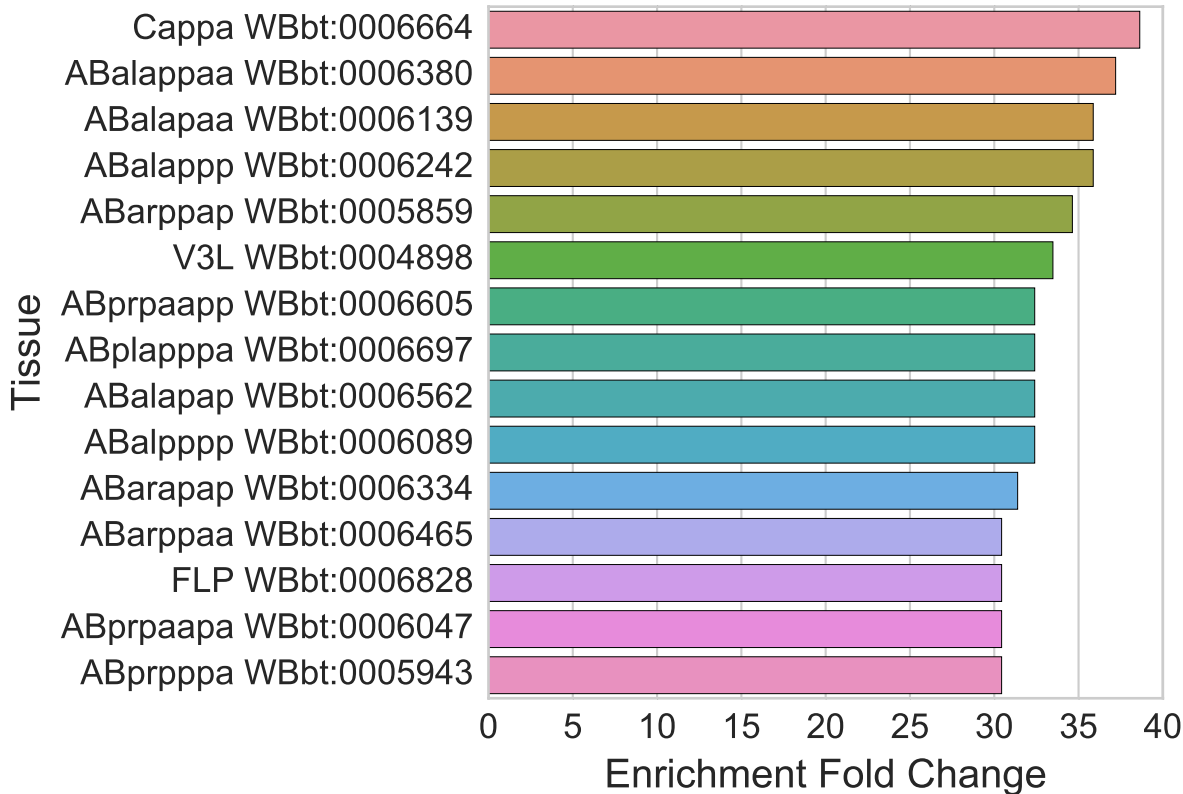

Supplement: Additional file 4 — Results. A folder containing a complete version of the results we generated for this paper. (ZIP 1597 kb) [file 12859_2016_1229_MOESM4_ESM.zip › output/HGT25_any_Results/WBPaper00013489_Ray_Enriched_WBbt_0006941_25.pdf]

Tissue

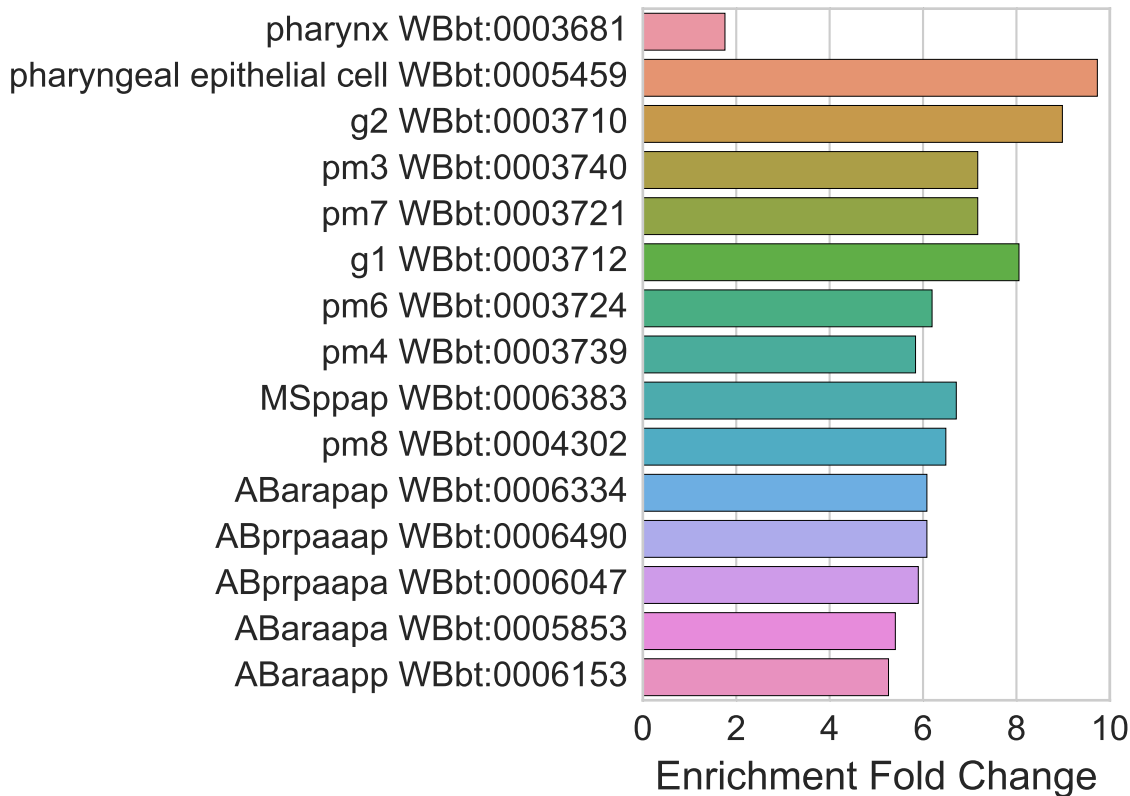

Supplement: Additional file 4 — Results. A folder containing a complete version of the results we generated for this paper. (ZIP 1597 kb) [file 12859_2016_1229_MOESM4_ESM.zip › output/HGT25_any_Results/WBPaper00024505_pharyngeal_enriched_WBbt_0003681_329.pdf]

Tissue

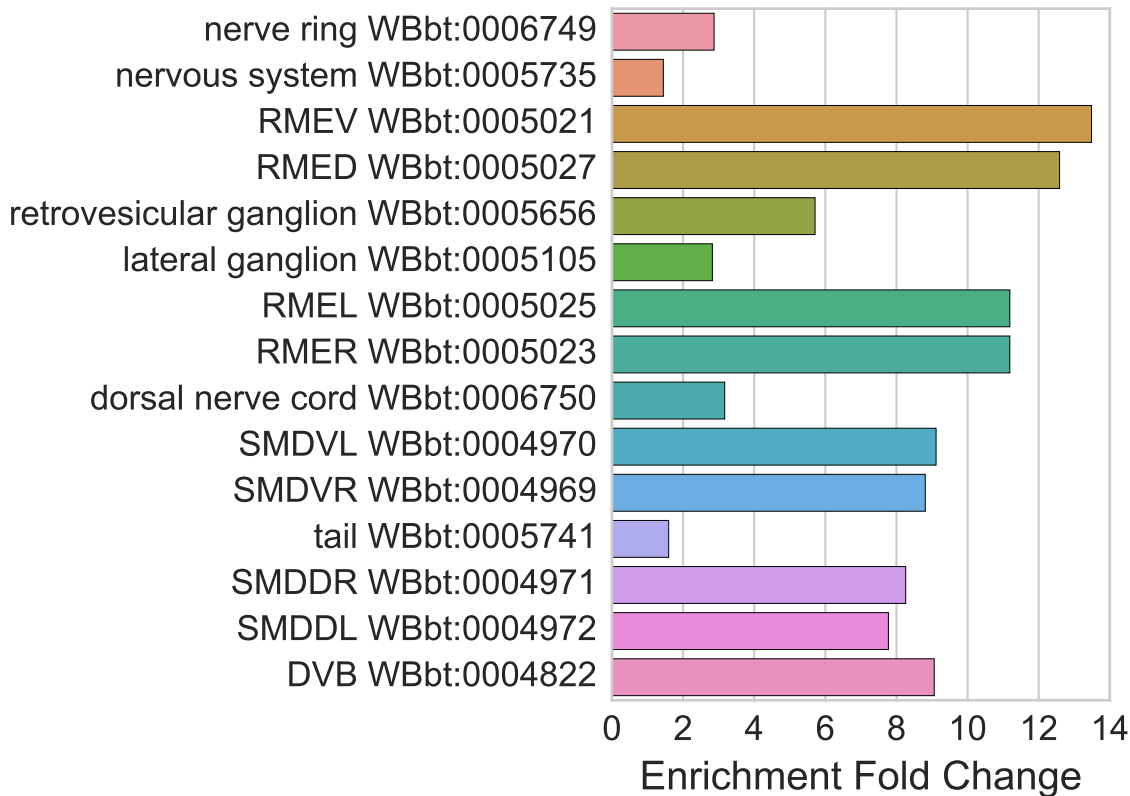

Supplement: Additional file 4 — Results. A folder containing a complete version of the results we generated for this paper. (ZIP 1597 kb) [file 12859_2016_1229_MOESM4_ESM.zip › output/HGT25_any_Results/WBPaper00024970_GABAergic_neuron_specific_WBbt_0005190_247.pdf]

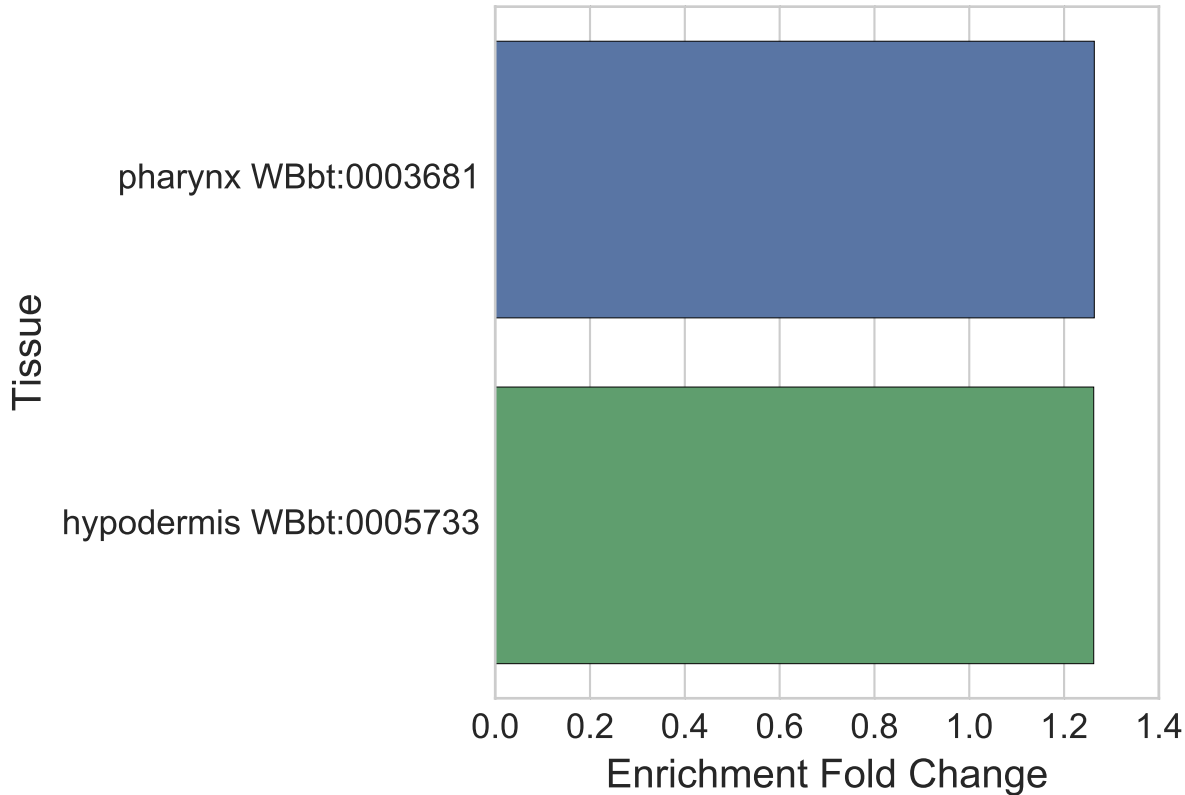

Supplement: Additional file 4 — Results. A folder containing a complete version of the results we generated for this paper. (ZIP 1597 kb) [file 12859_2016_1229_MOESM4_ESM.zip › output/HGT25_any_Results/WBPaper00026980_intestine_enriched_WBbt_0005772_1970.pdf]

Tissue

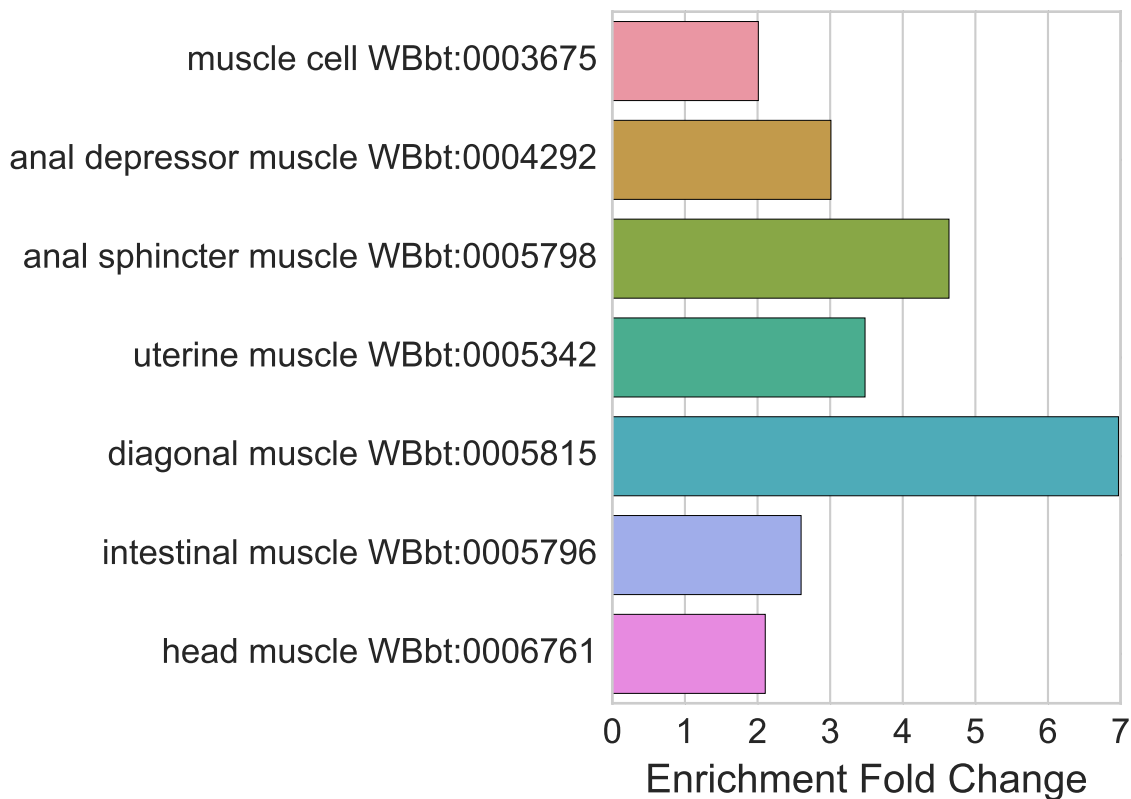

Supplement: Additional file 4 — Results. A folder containing a complete version of the results we generated for this paper. (ZIP 1597 kb) [file 12859_2016_1229_MOESM4_ESM.zip › output/HGT25_any_Results/WBPaper00031003_0hr_muscle_enriched_WBbt_0003675_761.pdf]

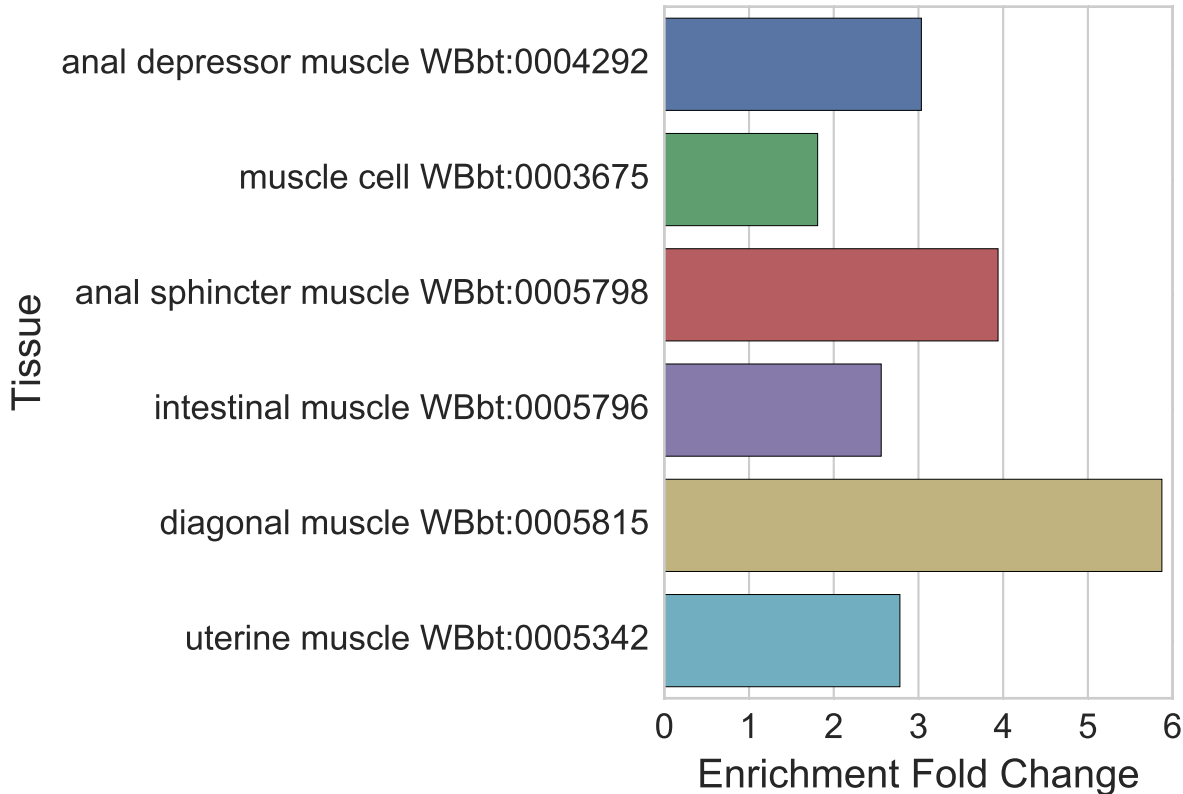

Supplement: Additional file 4 — Results. A folder containing a complete version of the results we generated for this paper. (ZIP 1597 kb) [file 12859_2016_1229_MOESM4_ESM.zip › output/HGT25_any_Results/WBPaper00031003_24hr_muscle_enriched_WBbt_0003675_918.pdf]

Tissue

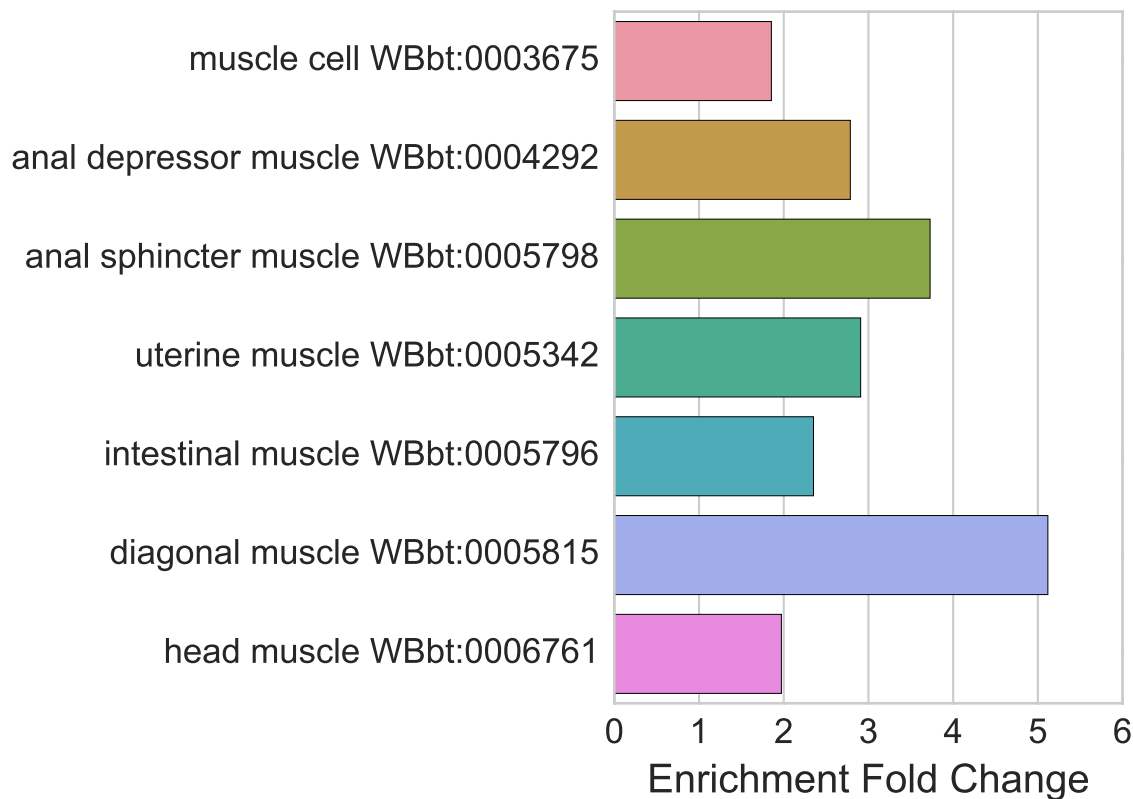

Supplement: Additional file 4 — Results. A folder containing a complete version of the results we generated for this paper. (ZIP 1597 kb) [file 12859_2016_1229_MOESM4_ESM.zip › output/HGT25_any_Results/WBPaper00031003_total_muscle_enriched_WBbt_0003675_1285.pdf]

Tissue

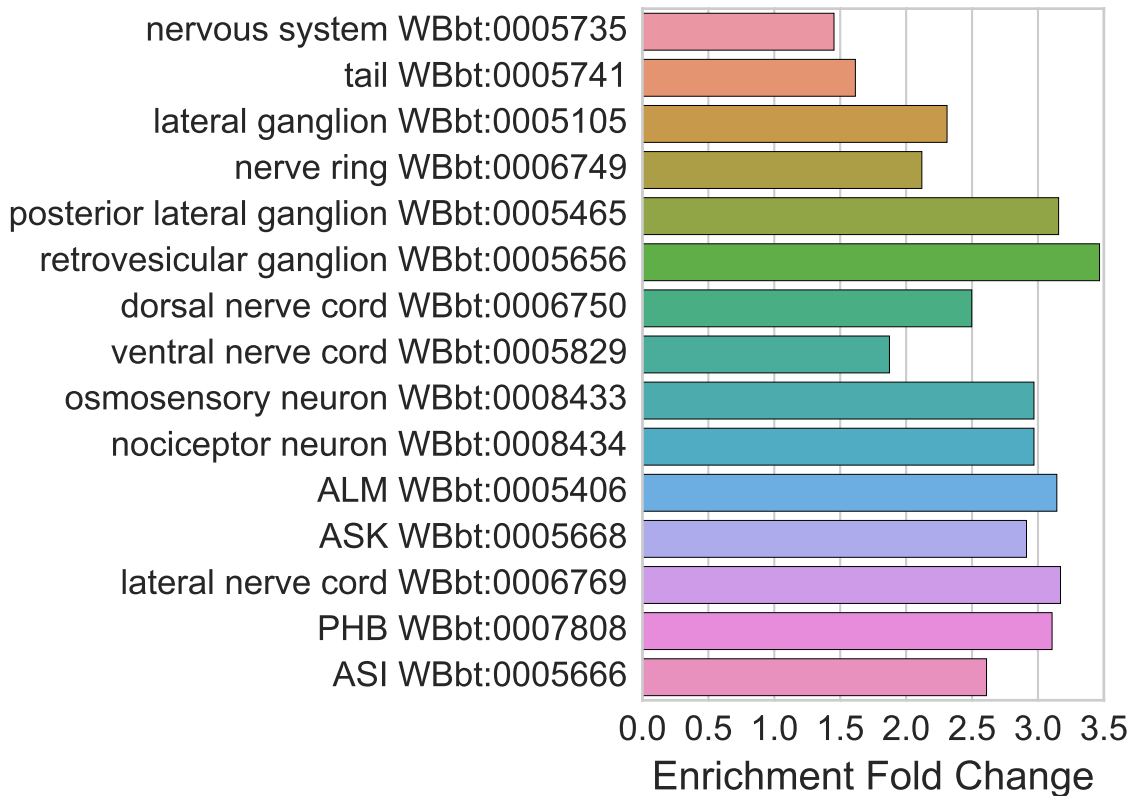

Supplement: Additional file 4 — Results. A folder containing a complete version of the results we generated for this paper. (ZIP 1597 kb) [file 12859_2016_1229_MOESM4_ESM.zip › output/HGT25_any_Results/WBPaper00031532_Larva_Pan_Neuronal_Enriched_WBbt_0003679_1603.pdf]

Tissue

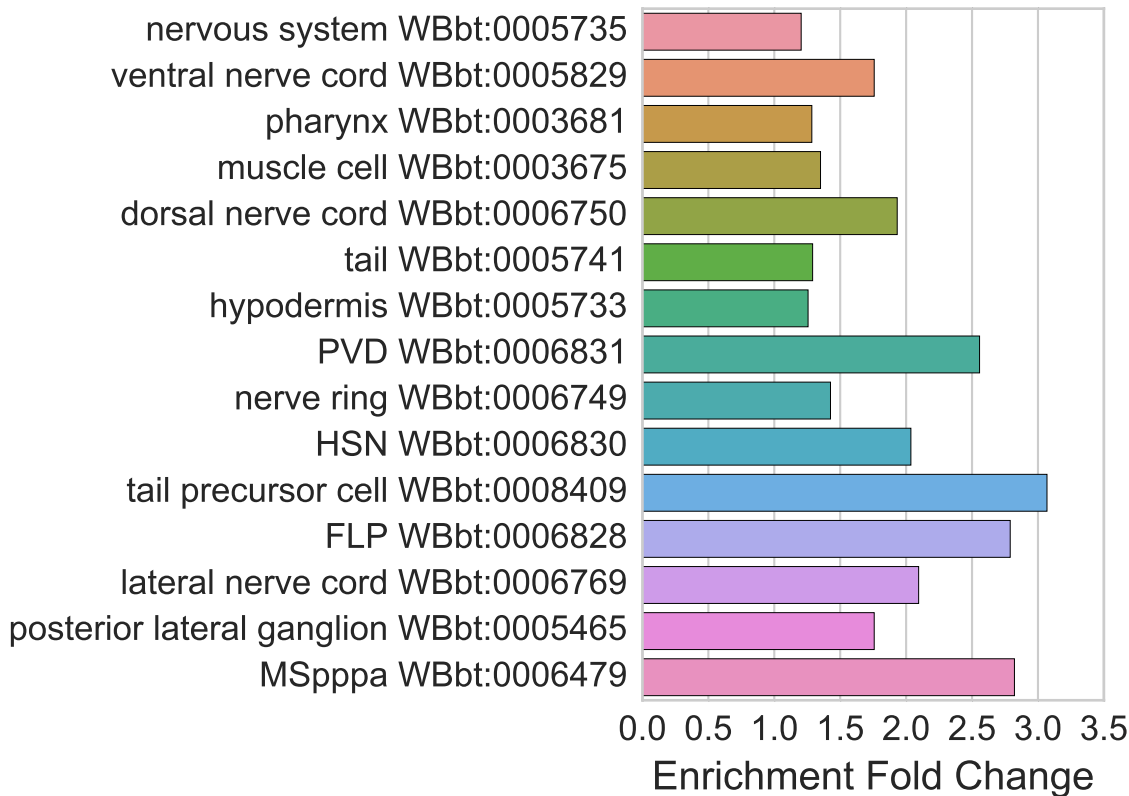

Supplement: Additional file 4 — Results. A folder containing a complete version of the results we generated for this paper. (ZIP 1597 kb) [file 12859_2016_1229_MOESM4_ESM.zip › output/HGT25_any_Results/WBPaper00036375_enriched_in_PVD_OLL_WBbt_0006831_2180.pdf]

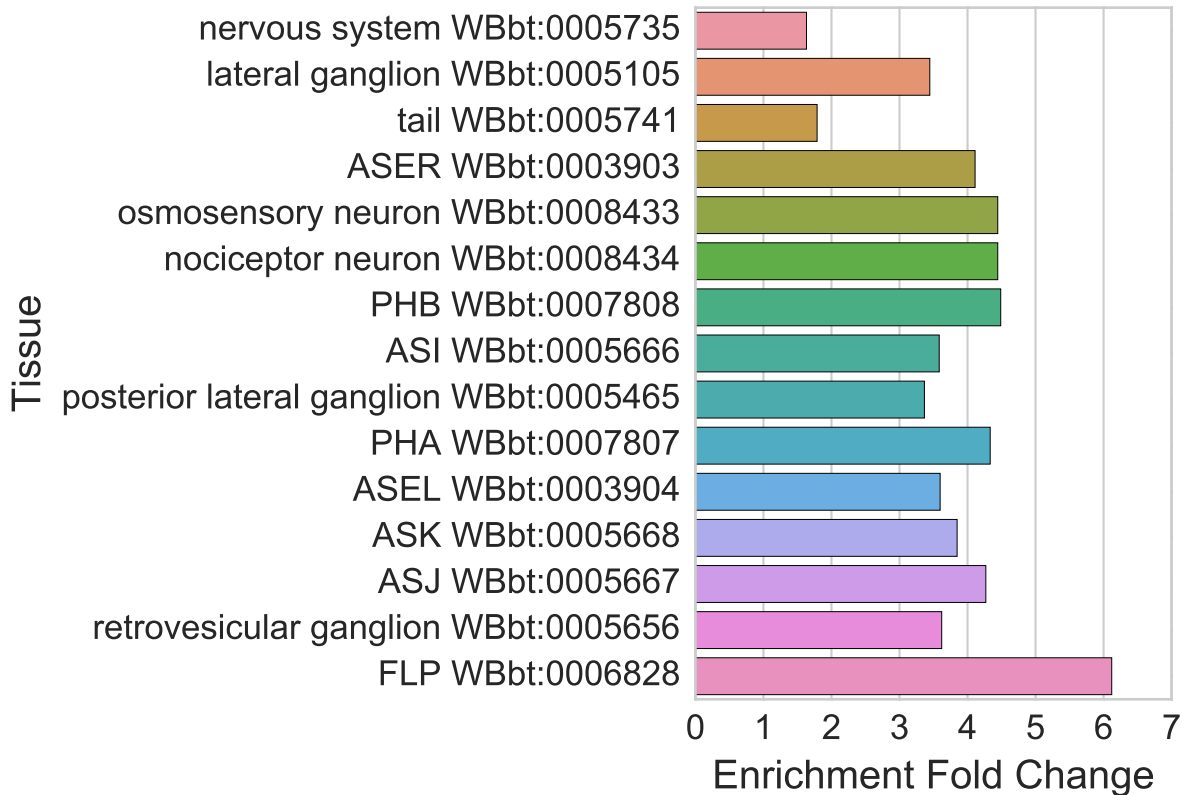

Supplement: Additional file 4 — Results. A folder containing a complete version of the results we generated for this paper. (ZIP 1597 kb) [file 12859_2016_1229_MOESM4_ESM.zip › output/HGT25_any_Results/WBPaper00037950_all-neurons_larva_enriched_WBbt_0003679_1013.pdf]

Tissue

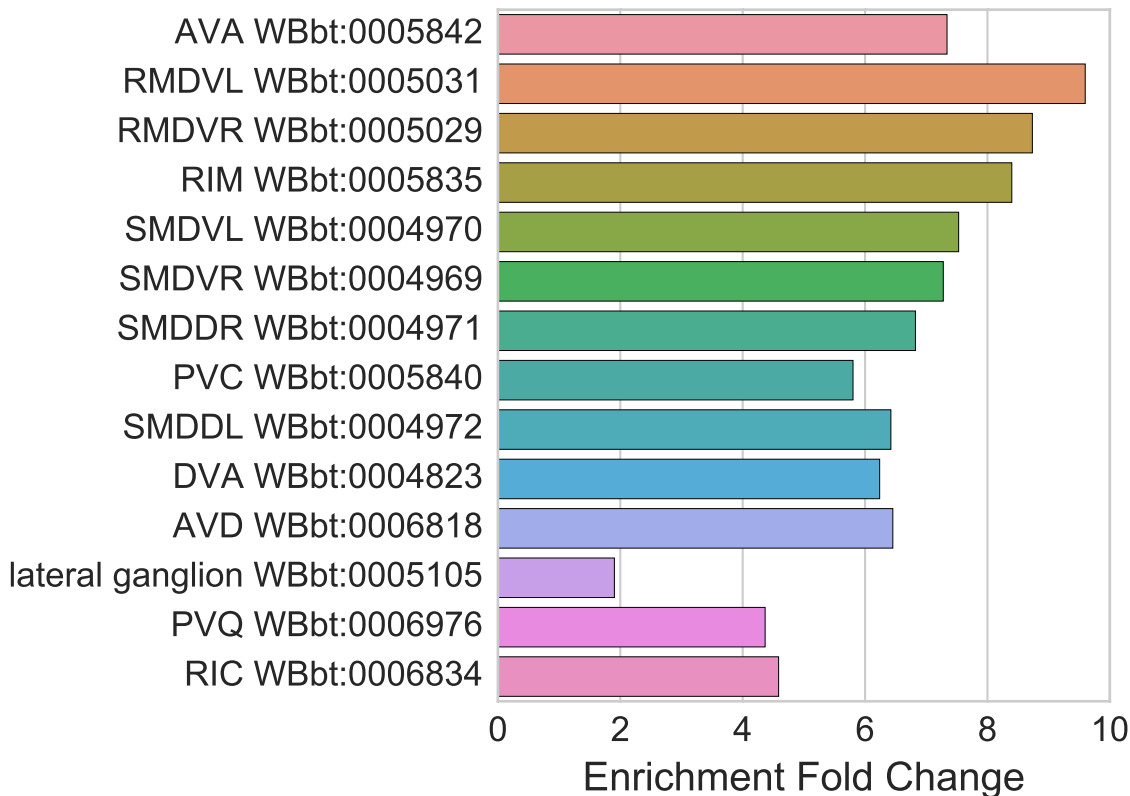

Supplement: Additional file 4 — Results. A folder containing a complete version of the results we generated for this paper. (ZIP 1597 kb) [file 12859_2016_1229_MOESM4_ESM.zip › output/HGT25_any_Results/WBPaper00037950_AVA-neuron_embryo_enriched_WBbt_0005842_534.pdf]

Tissue

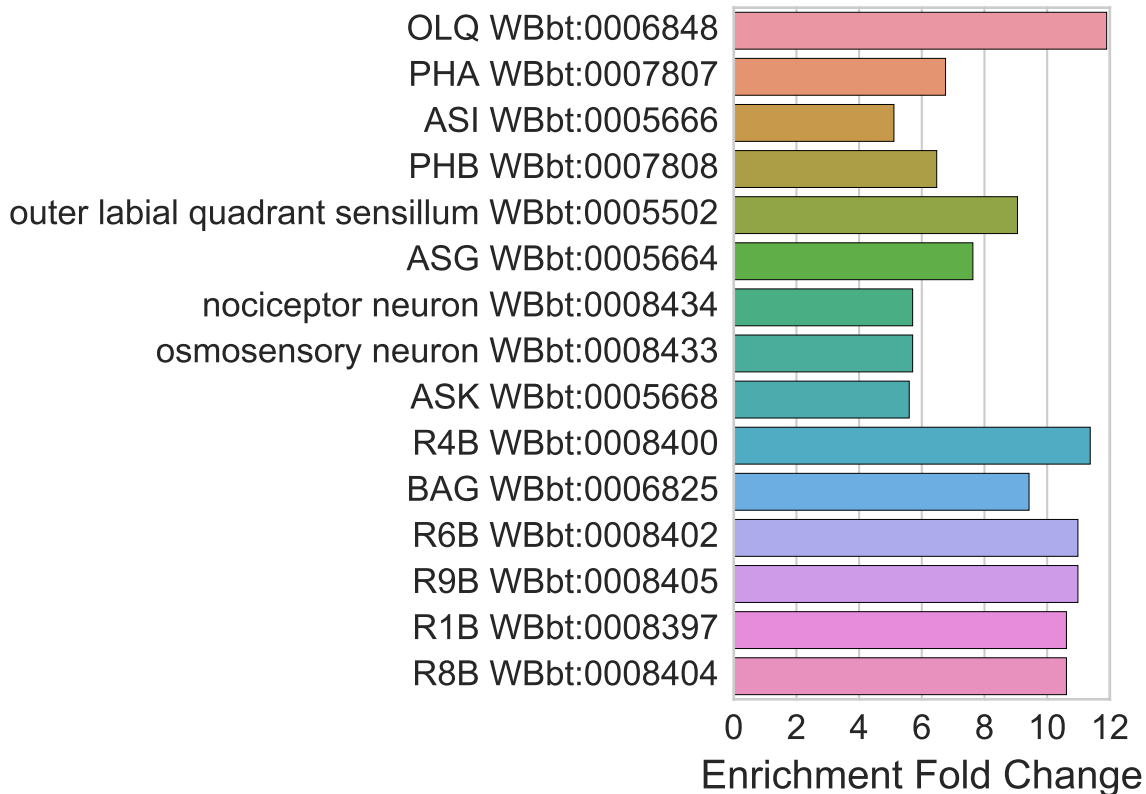

Supplement: Additional file 4 — Results. A folder containing a complete version of the results we generated for this paper. (ZIP 1597 kb) [file 12859_2016_1229_MOESM4_ESM.zip › output/HGT25_any_Results/WBPaper00037950_BAG-neuron_embryo_enriched_WBbt_0006825_454.pdf]

Tissue

coelomic system WBbt:0005749

hmc WBbt:0004697

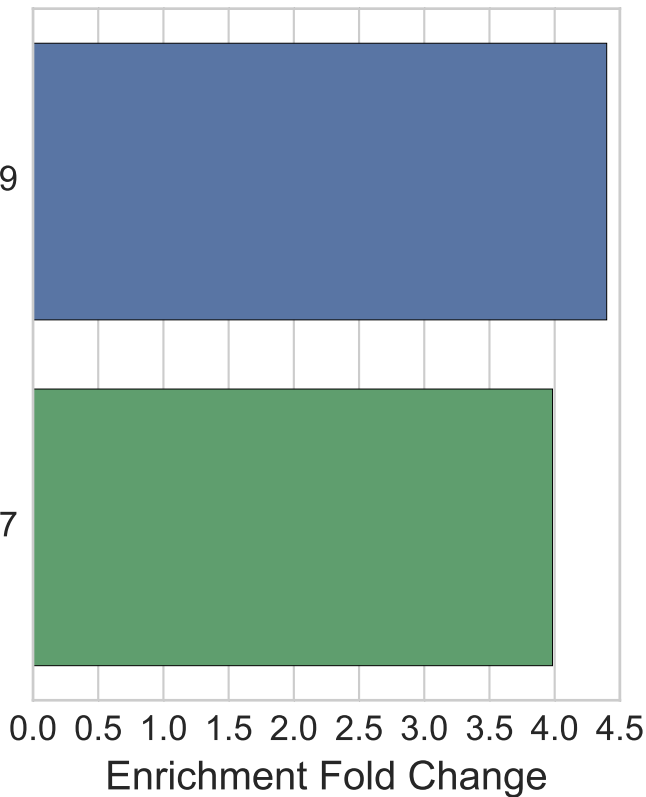

Supplement: Additional file 4 — Results. A folder containing a complete version of the results we generated for this paper. (ZIP 1597 kb) [file 12859_2016_1229_MOESM4_ESM.zip › output/HGT25_any_Results/WBPaper00037950_coelomocytes_embryo_enriched_WBbt_0005751_570.pdf]

Tissue

coelomic system WBbt:0005749

0

1

2

3

4

5

Enrichment Fold Change

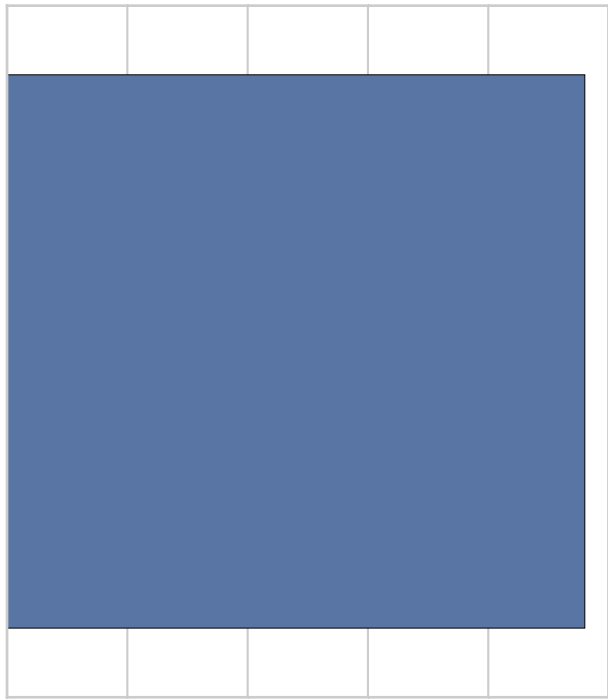

Supplement: Additional file 4 — Results. A folder containing a complete version of the results we generated for this paper. (ZIP 1597 kb) [file 12859_2016_1229_MOESM4_ESM.zip › output/HGT25_any_Results/WBPaper00037950_coelomocytes_larva_enriched_WBbt_0005751_229.pdf]

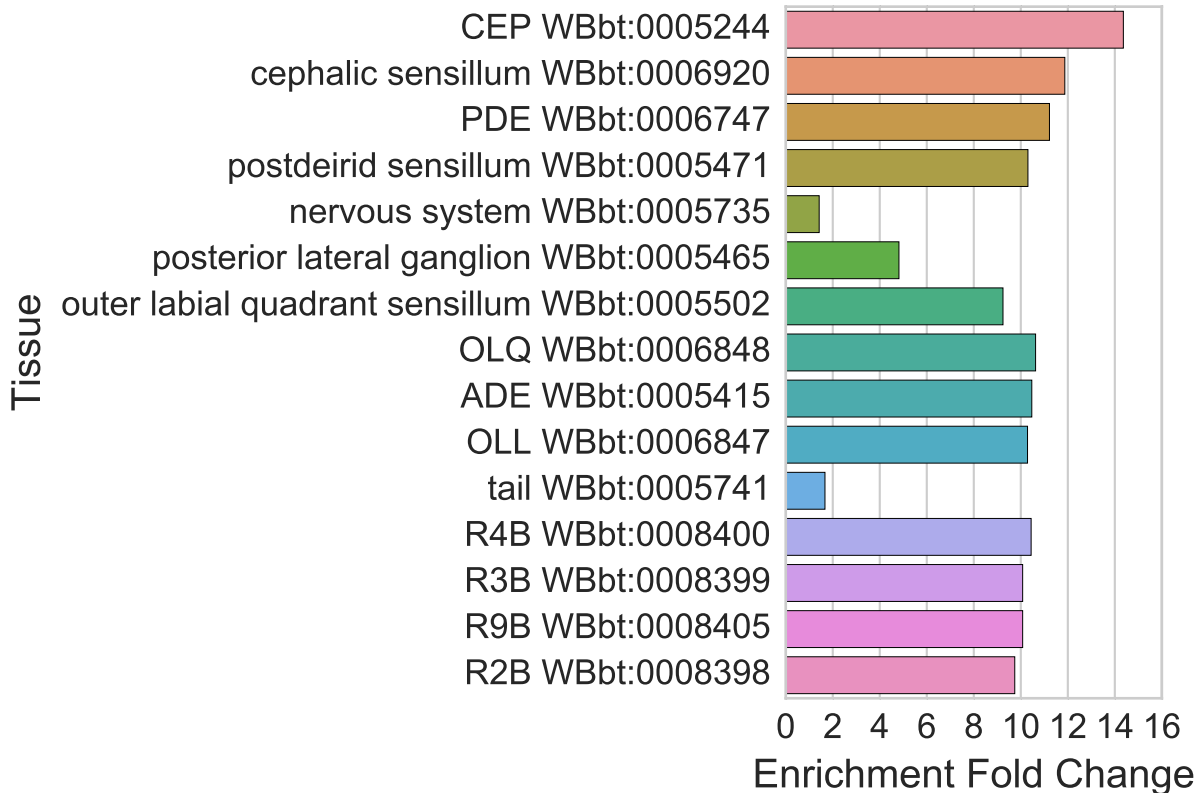

Supplement: Additional file 4 — Results. A folder containing a complete version of the results we generated for this paper. (ZIP 1597 kb) [file 12859_2016_1229_MOESM4_ESM.zip › output/HGT25_any_Results/WBPaper00037950_dopaminergic-neurons_embryo_enriched_WBbt_0006746_466.pdf]

Tissue

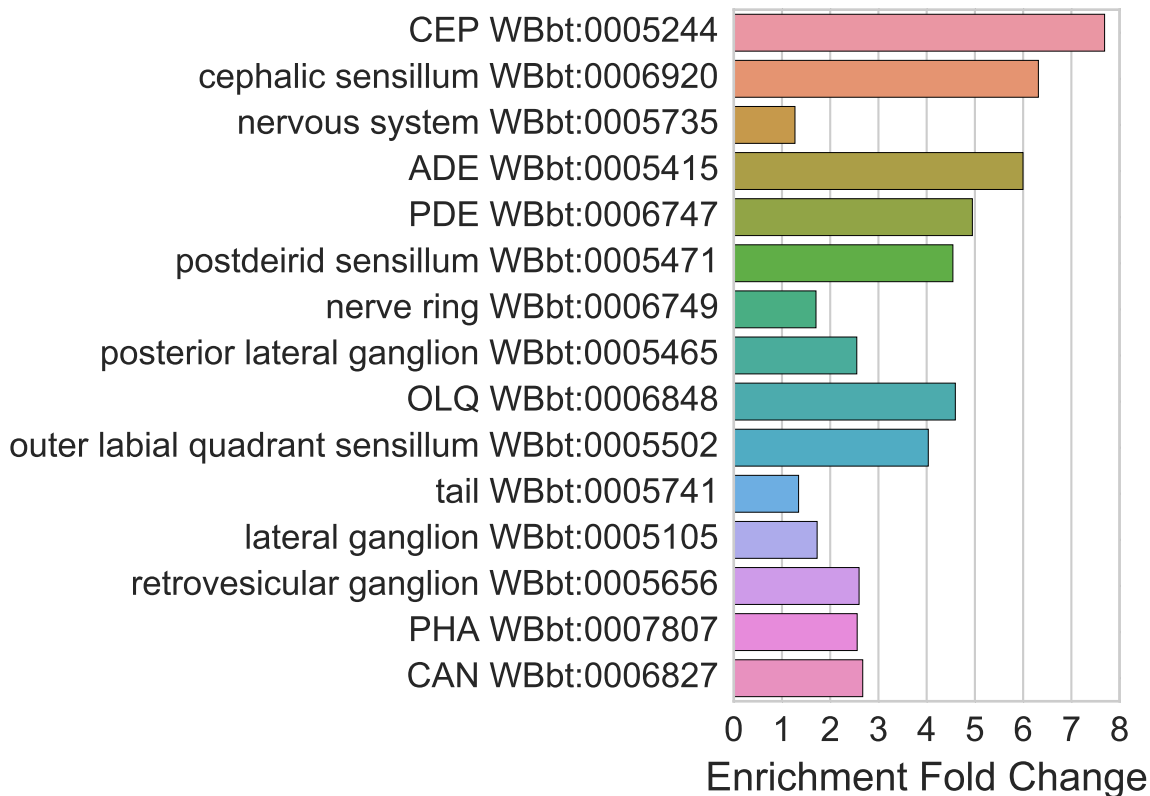

Supplement: Additional file 4 — Results. A folder containing a complete version of the results we generated for this paper. (ZIP 1597 kb) [file 12859_2016_1229_MOESM4_ESM.zip › output/HGT25_any_Results/WBPaper00037950_dopaminergic-neurons_larva_enriched_WBbt_0006746_1230.pdf]

Tissue

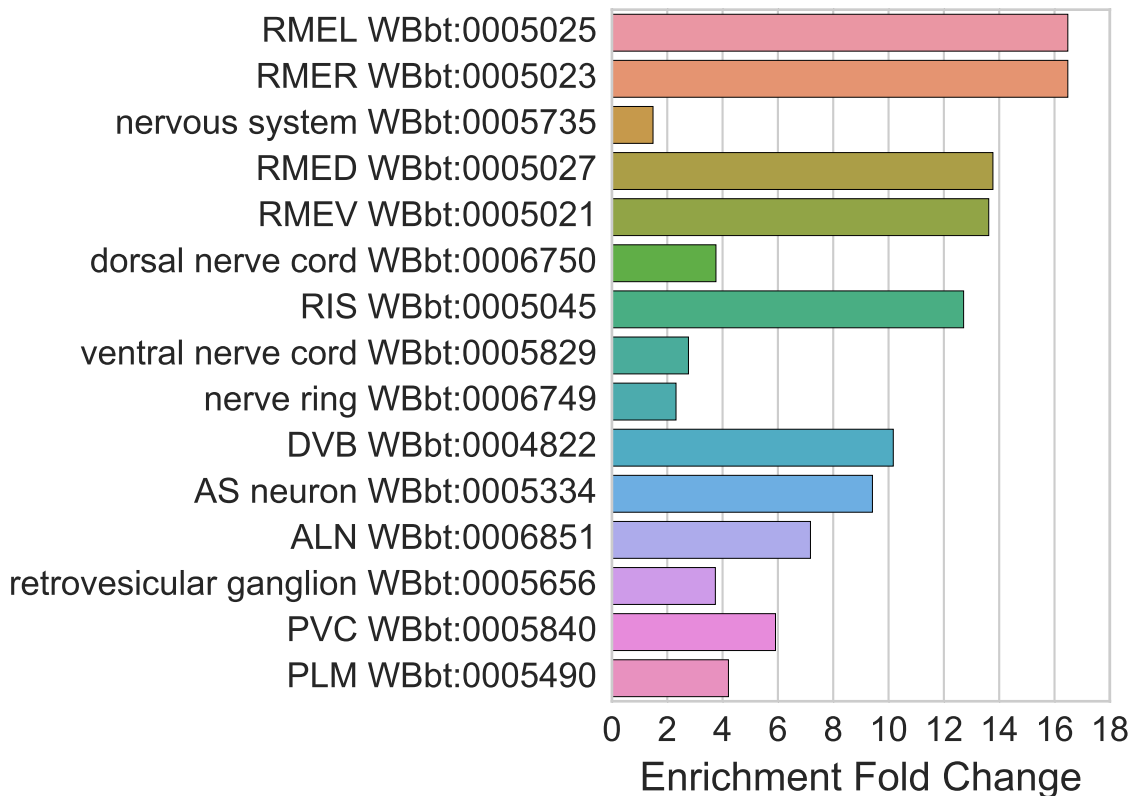

Supplement: Additional file 4 — Results. A folder containing a complete version of the results we generated for this paper. (ZIP 1597 kb) [file 12859_2016_1229_MOESM4_ESM.zip › output/HGT25_any_Results/WBPaper00037950_GABAergic-motor-neurons_embryo_enriched_WBbt_0005190_361.pdf]

Tissue

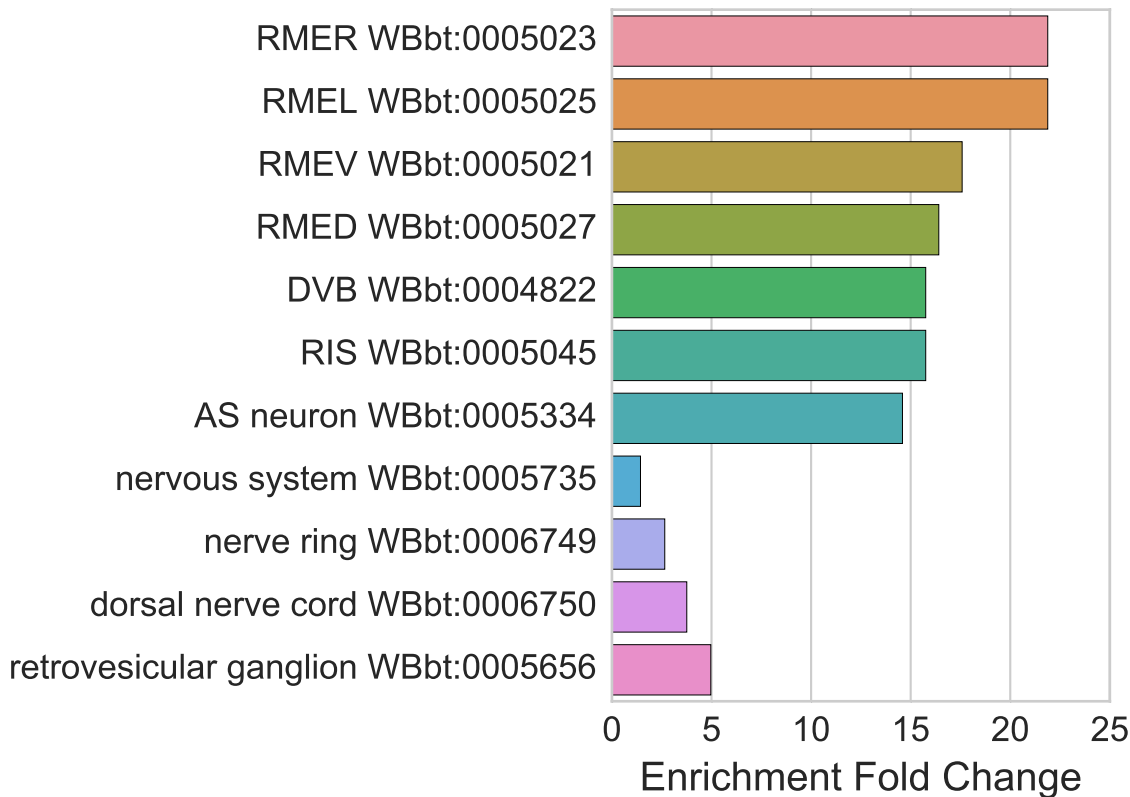

Supplement: Additional file 4 — Results. A folder containing a complete version of the results we generated for this paper. (ZIP 1597 kb) [file 12859_2016_1229_MOESM4_ESM.zip › output/HGT25_any_Results/WBPaper00037950_GABAergic-motor-neurons_larva_enriched_WBbt_0005190_132.pdf]

Tissue

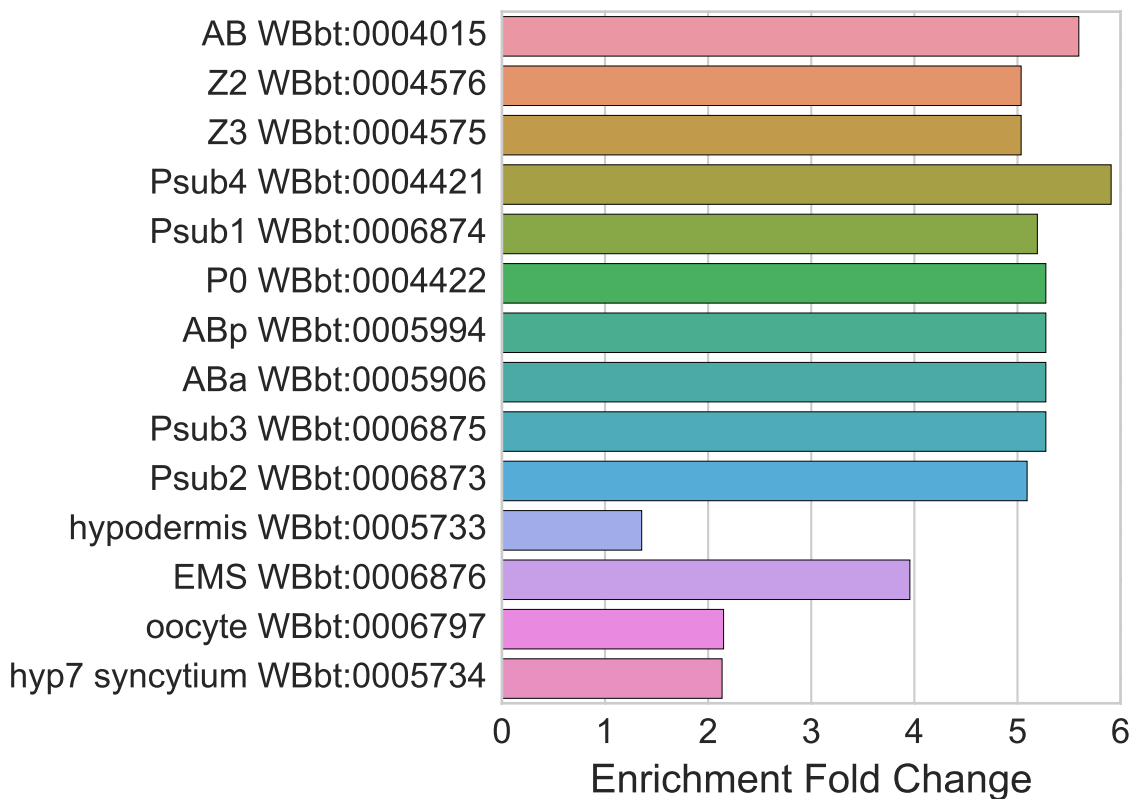

Supplement: Additional file 4 — Results. A folder containing a complete version of the results we generated for this paper. (ZIP 1597 kb) [file 12859_2016_1229_MOESM4_ESM.zip › output/HGT25_any_Results/WBPaper00037950_germline-precursors_embryo_enriched_WBbt_0006849_974.pdf]

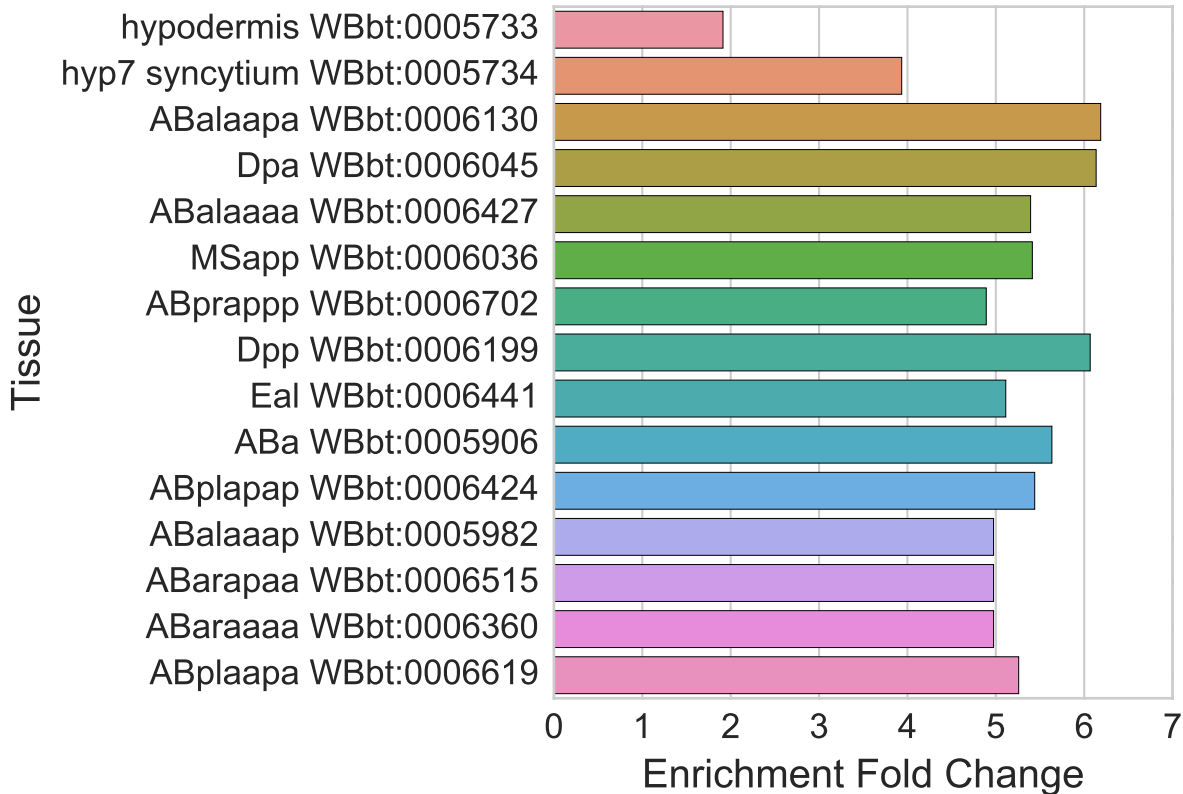

Supplement: Additional file 4 — Results. A folder containing a complete version of the results we generated for this paper. (ZIP 1597 kb) [file 12859_2016_1229_MOESM4_ESM.zip › output/HGT25_any_Results/WBPaper00037950_hypodermis_embryo_enriched_WBbt_0005733_734.pdf]

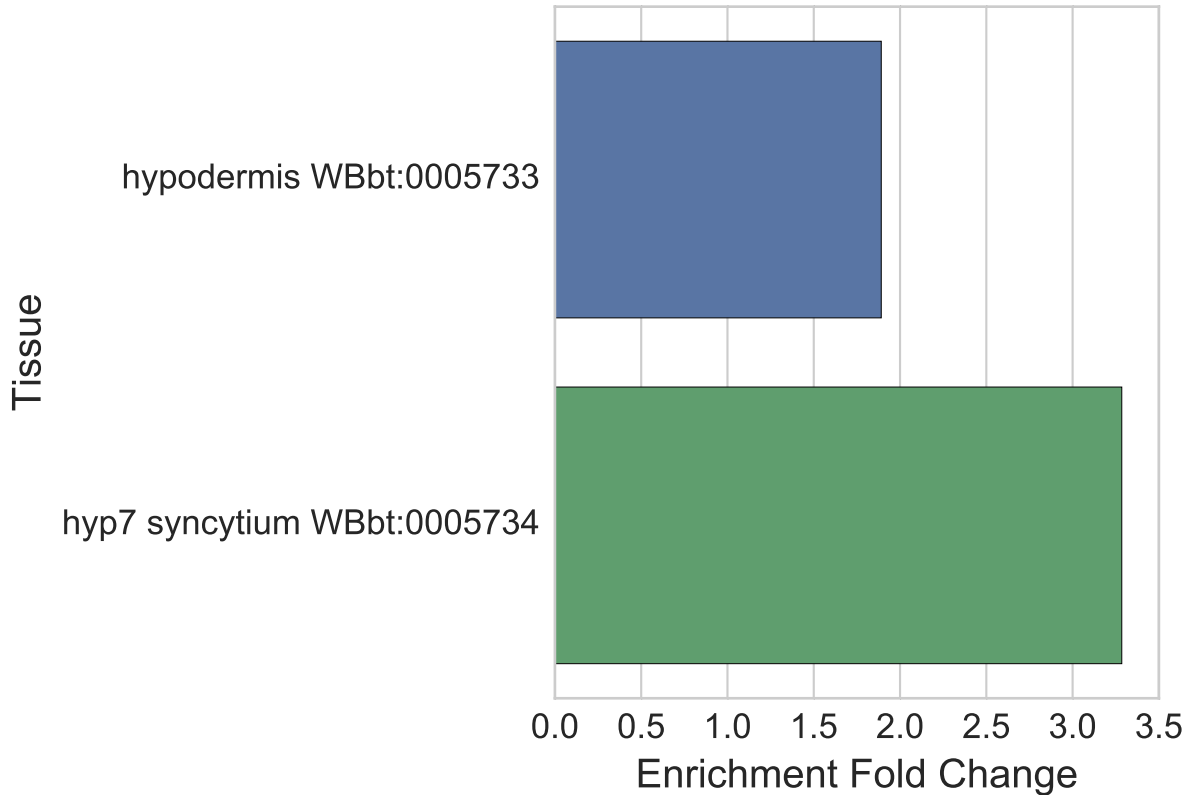

Supplement: Additional file 4 — Results. A folder containing a complete version of the results we generated for this paper. (ZIP 1597 kb) [file 12859_2016_1229_MOESM4_ESM.zip › output/HGT25_any_Results/WBPaper00037950_hypodermis_larva_enriched_WBbt_0005733_1250.pdf]

Tissue

PVD WBbt:0006831

0.0 0.5 1.0 1.5 2.0 2.5 3.0 3.5

Enrichment Fold Change

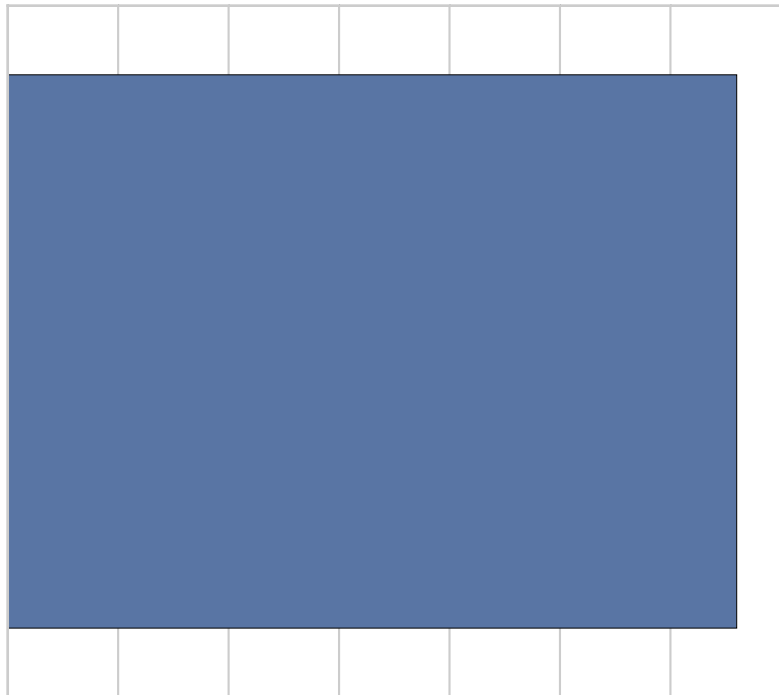

Supplement: Additional file 4 — Results. A folder containing a complete version of the results we generated for this paper. (ZIP 1597 kb) [file 12859_2016_1229_MOESM4_ESM.zip › output/HGT25_any_Results/WBPaper00037950_PVD-OLL-neurons_larva_enriched_WBbt_0006831_878.pdf]

Tissue

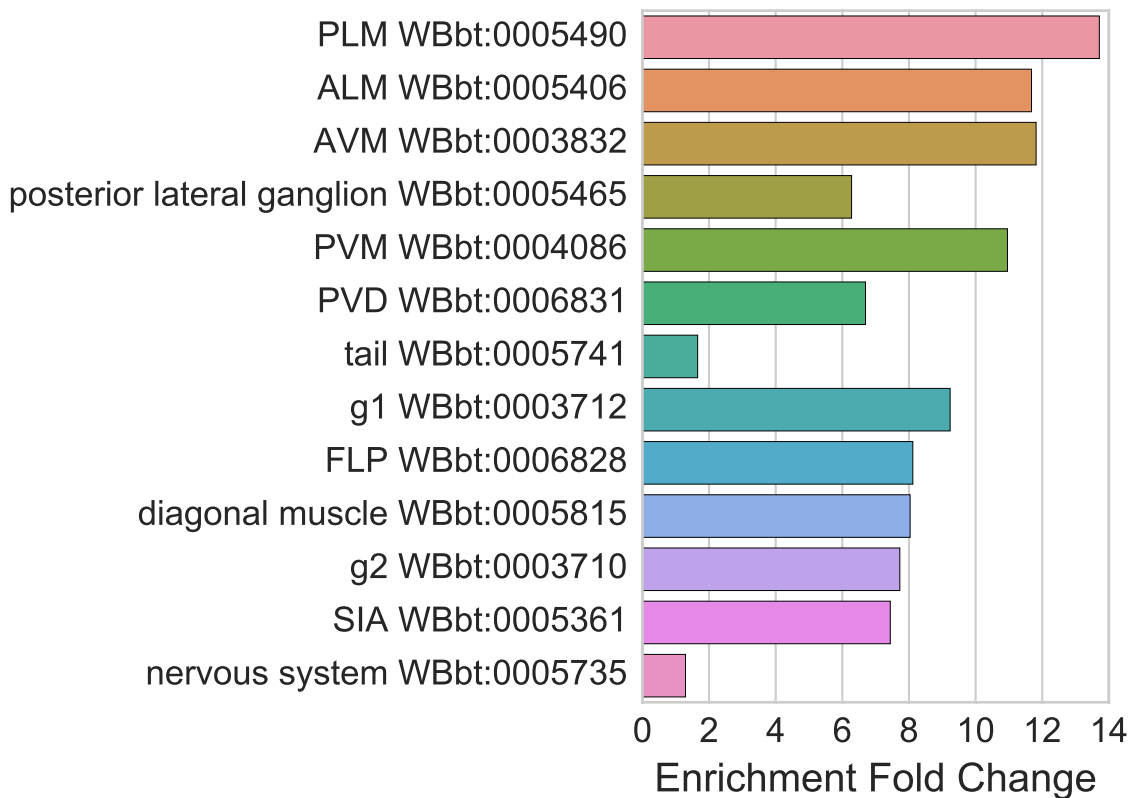

Supplement: Additional file 4 — Results. A folder containing a complete version of the results we generated for this paper. (ZIP 1597 kb) [file 12859_2016_1229_MOESM4_ESM.zip › output/HGT25_any_Results/WBPaper00040420_ALM_PLM_enriched_WBbt_0005406_198.pdf]

Tissue

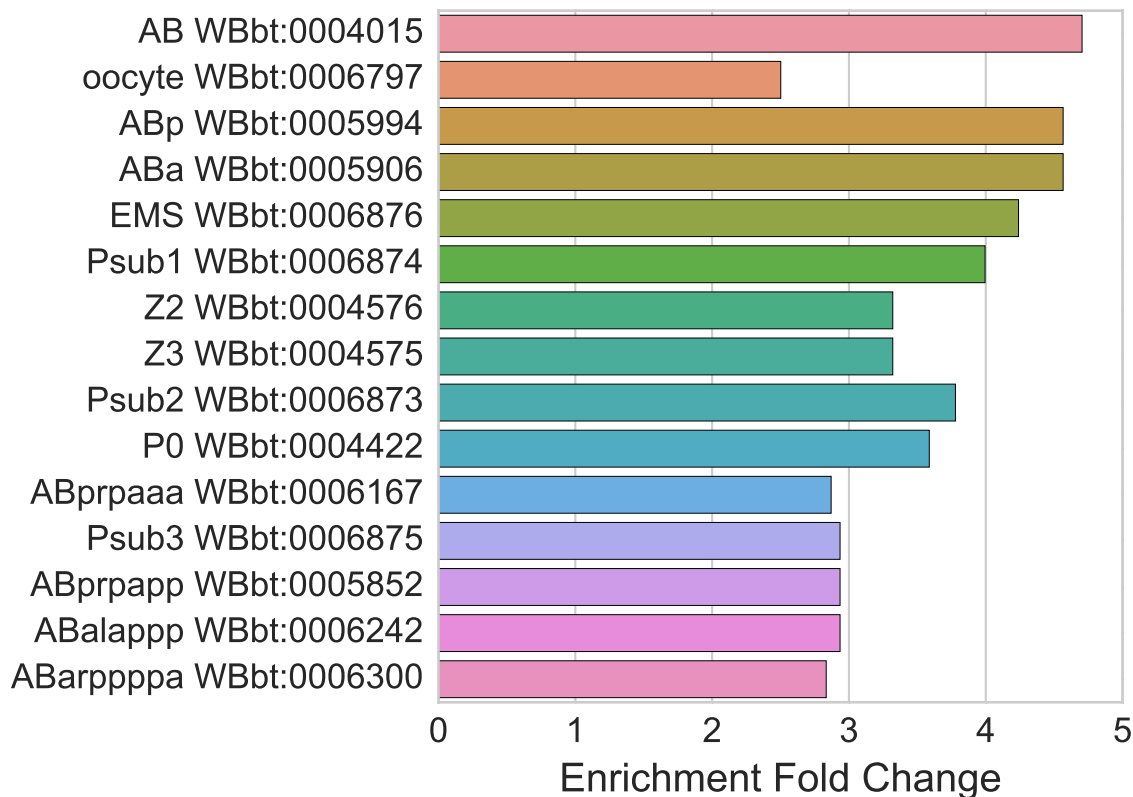

Supplement: Additional file 4 — Results. A folder containing a complete version of the results we generated for this paper. (ZIP 1597 kb) [file 12859_2016_1229_MOESM4_ESM.zip › output/HGT25_any_Results/WBPaper00044760_germline_specific_WBbt_0005784_2510.pdf]

Tissue

oocyte WBbt:0006797

gonadal sheath cell WBbt:0005828

spermatheca WBbt:0005319

0.0 0.5 1.0 1.5 2.0 2.5 3.0  
Enrichment Fold Change

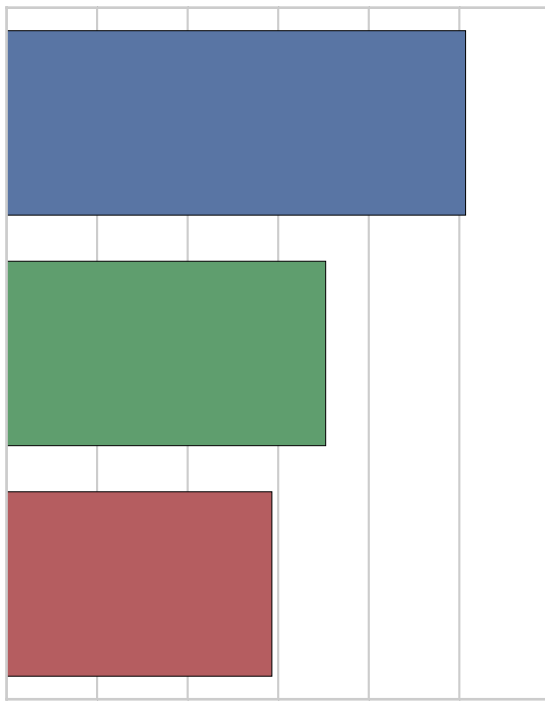

Supplement: Additional file 4 — Results. A folder containing a complete version of the results we generated for this paper. (ZIP 1597 kb) [file 12859_2016_1229_MOESM4_ESM.zip › output/HGT25_any_Results/WBPaper00045521_Spermatogenic_WBbt_0005784_2743.pdf]

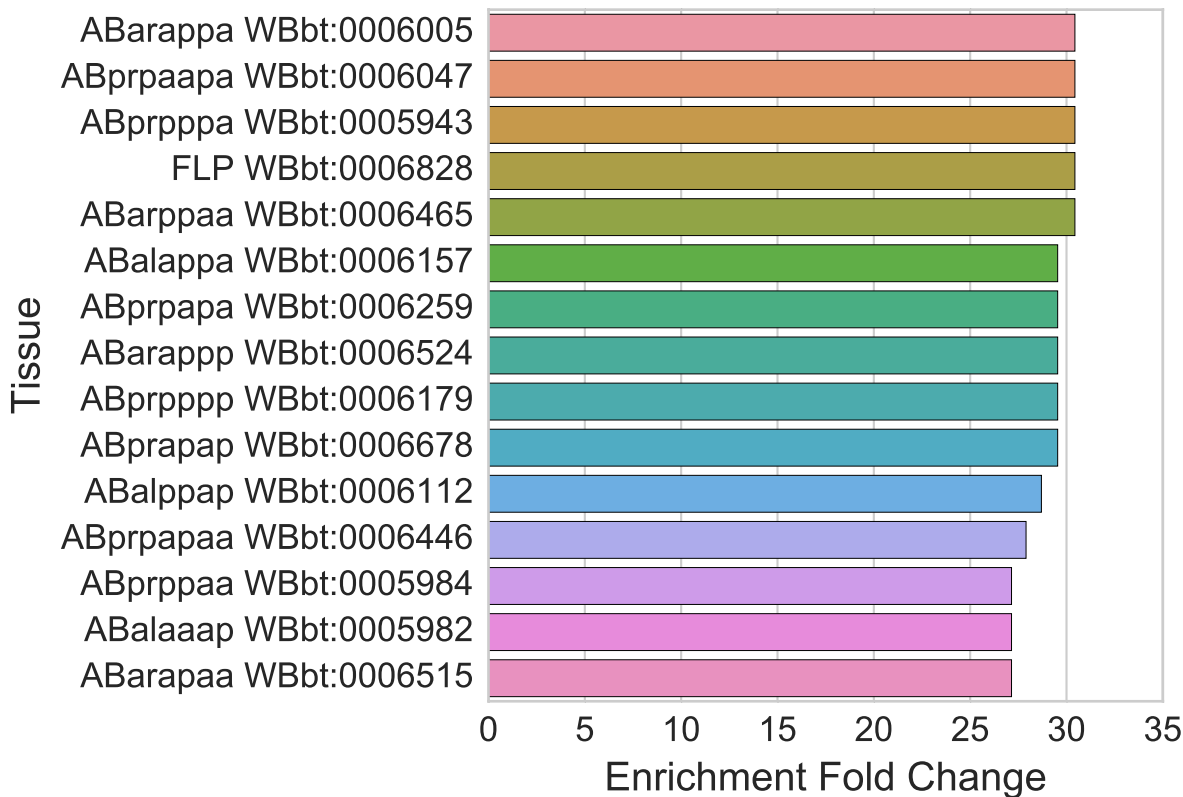

Supplement: Additional file 4 — Results. A folder containing a complete version of the results we generated for this paper. (ZIP 1597 kb) [file 12859_2016_1229_MOESM4_ESM.zip › output/HGT33_any_Results/WBPaper00013489_Ray_Enriched_WBbt_0006941_25.pdf]

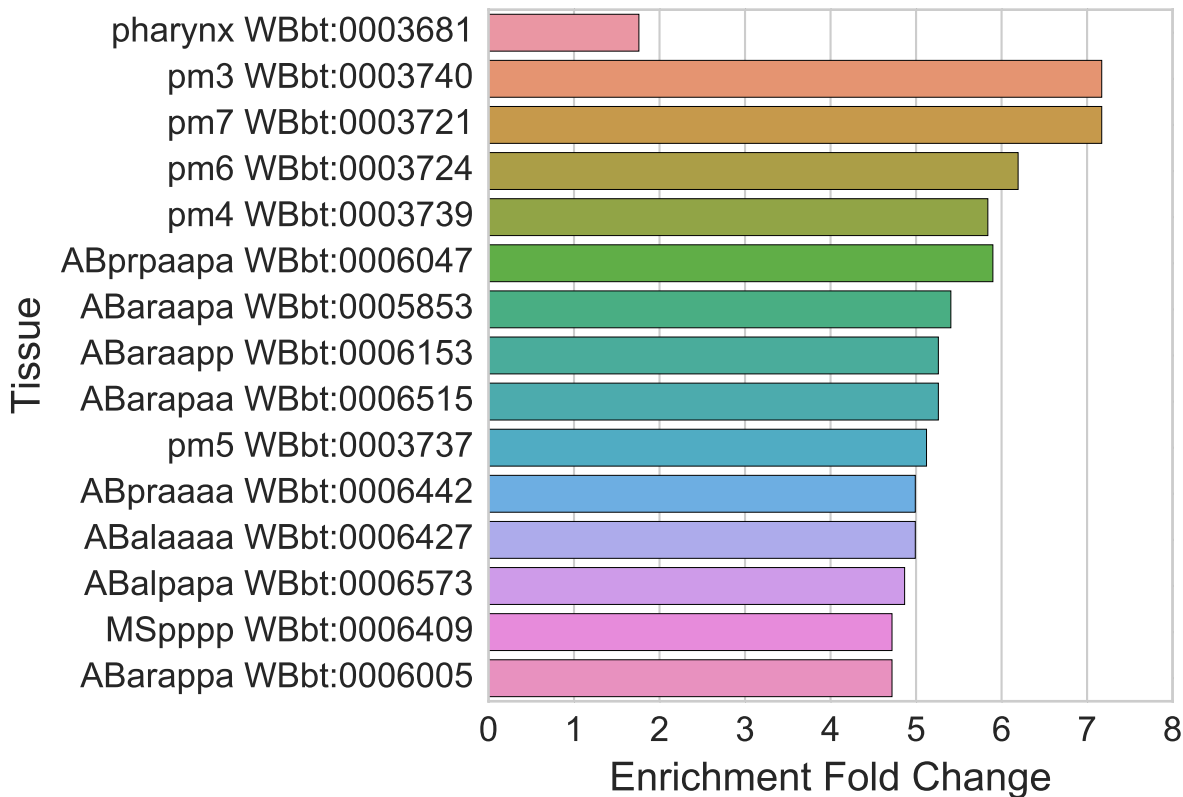

Supplement: Additional file 4 — Results. A folder containing a complete version of the results we generated for this paper. (ZIP 1597 kb) [file 12859_2016_1229_MOESM4_ESM.zip › output/HGT33_any_Results/WBPaper00024505_pharyngeal_enriched_WBbt_0003681_329.pdf]

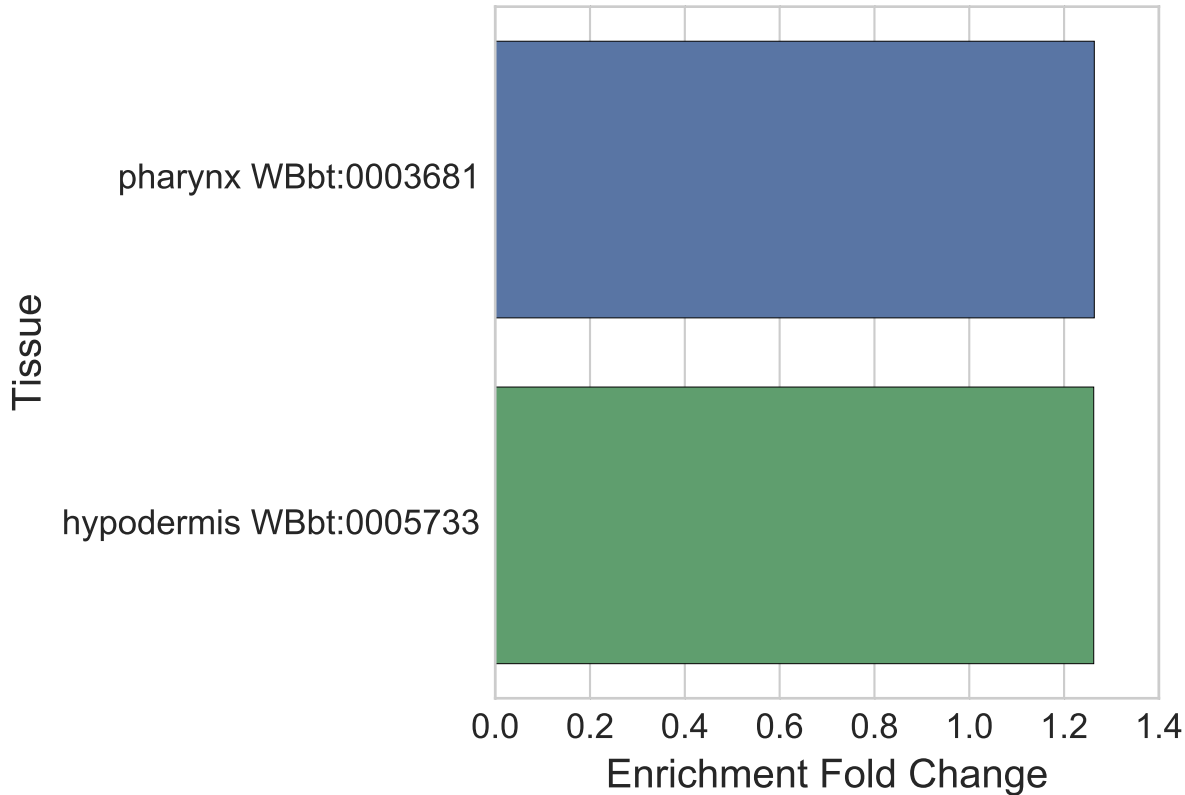

Supplement: Additional file 4 — Results. A folder containing a complete version of the results we generated for this paper. (ZIP 1597 kb) [file 12859_2016_1229_MOESM4_ESM.zip › output/HGT33_any_Results/WBPaper00026980_intestine_enriched_WBbt_0005772_1970.pdf]

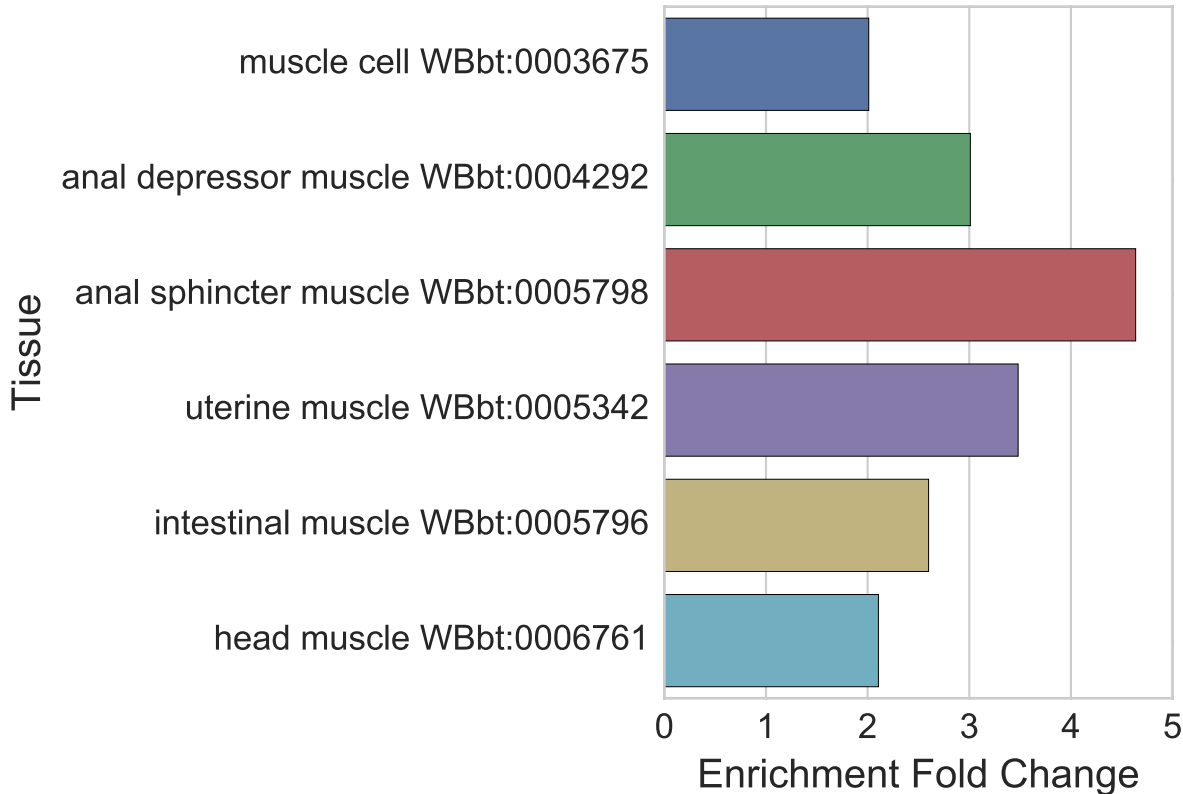

Supplement: Additional file 4 — Results. A folder containing a complete version of the results we generated for this paper. (ZIP 1597 kb) [file 12859_2016_1229_MOESM4_ESM.zip › output/HGT33_any_Results/WBPaper00031003_0hr_muscle_enriched_WBbt_0003675_761.pdf]

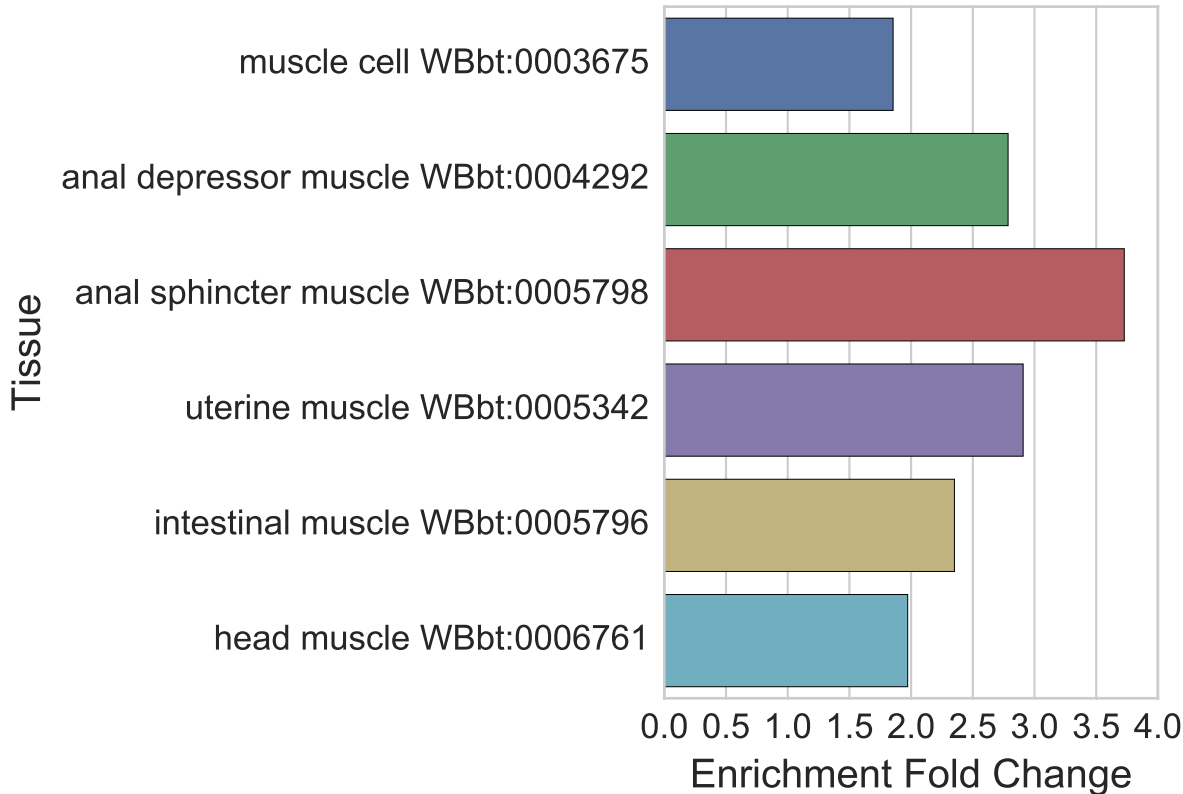

Supplement: Additional file 4 — Results. A folder containing a complete version of the results we generated for this paper. (ZIP 1597 kb) [file 12859_2016_1229_MOESM4_ESM.zip › output/HGT33_any_Results/WBPaper00031003_total_muscle_enriched_WBbt_0003675_1285.pdf]

Tissue

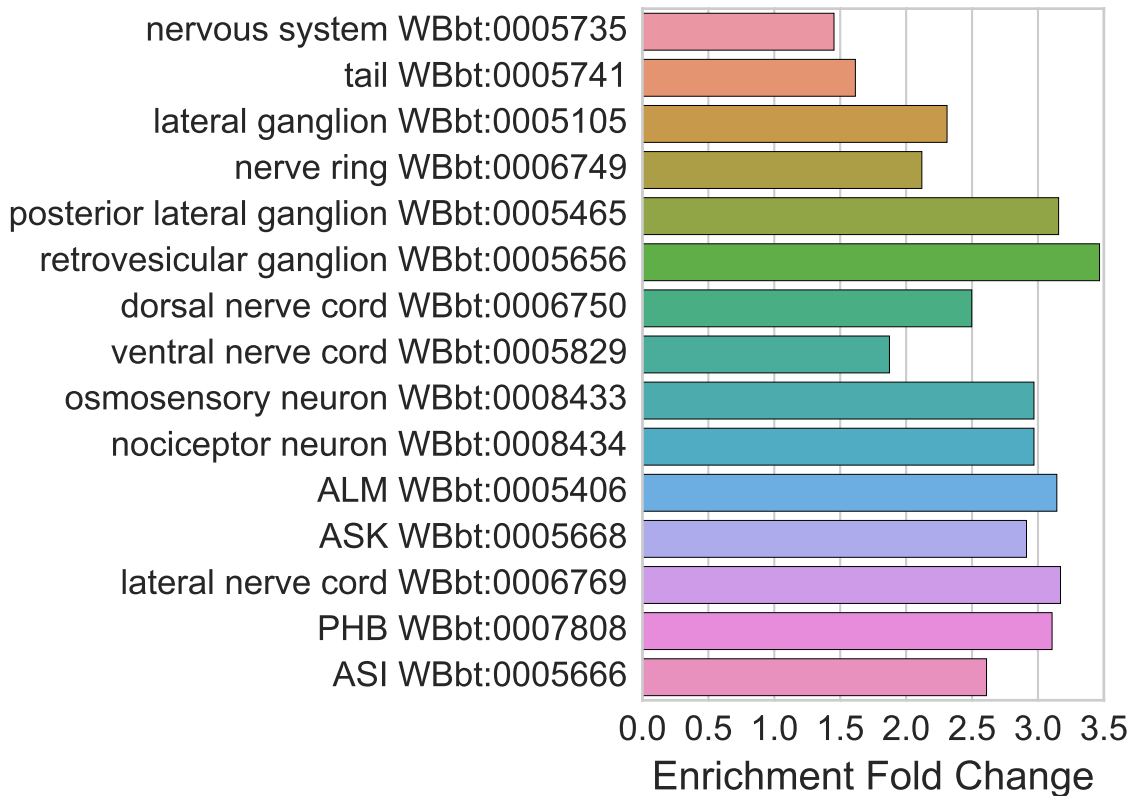

Supplement: Additional file 4 — Results. A folder containing a complete version of the results we generated for this paper. (ZIP 1597 kb) [file 12859_2016_1229_MOESM4_ESM.zip › output/HGT33_any_Results/WBPaper00031532_Larva_Pan_Neuronal_Enriched_WBbt_0003679_1603.pdf]

Tissue

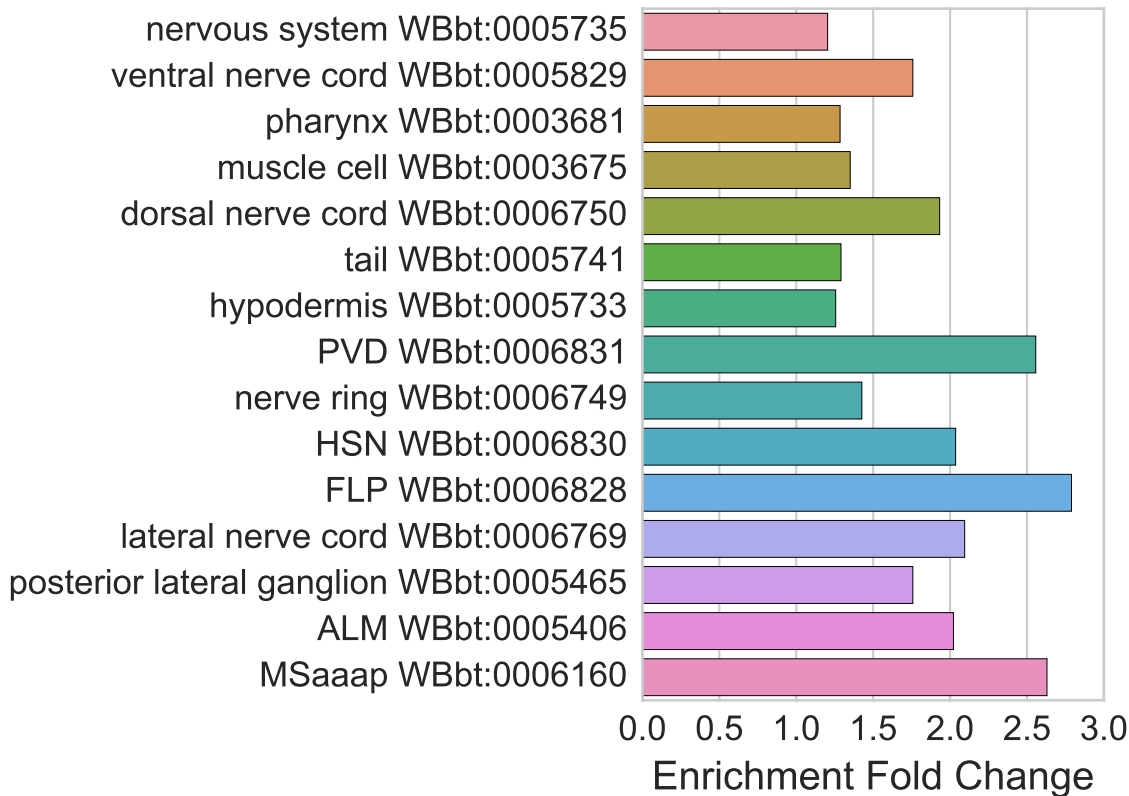

Supplement: Additional file 4 — Results. A folder containing a complete version of the results we generated for this paper. (ZIP 1597 kb) [file 12859_2016_1229_MOESM4_ESM.zip › output/HGT33_any_Results/WBPaper00036375_enriched_in_PVD_OLL_WBbt_0006831_2180.pdf]

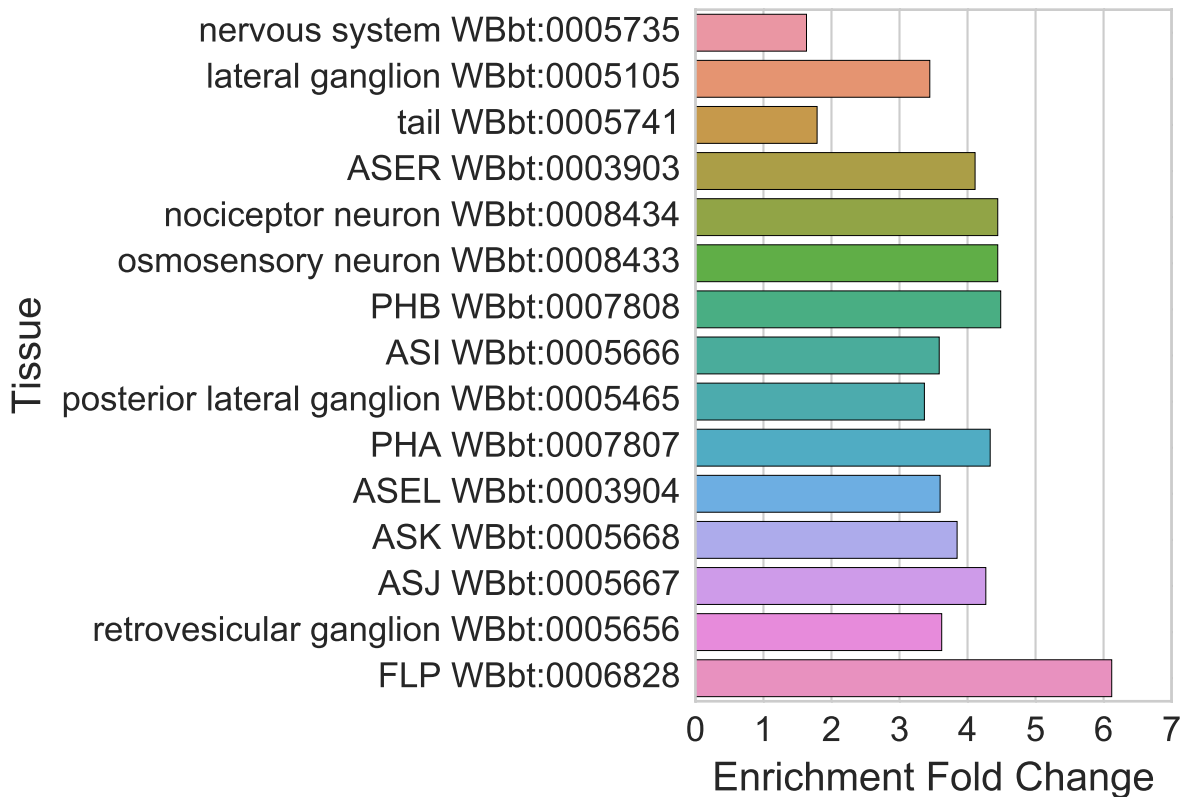

Supplement: Additional file 4 — Results. A folder containing a complete version of the results we generated for this paper. (ZIP 1597 kb) [file 12859_2016_1229_MOESM4_ESM.zip › output/HGT33_any_Results/WBPaper00037950_all-neurons_larva_enriched_WBbt_0003679_1013.pdf]

Tissue

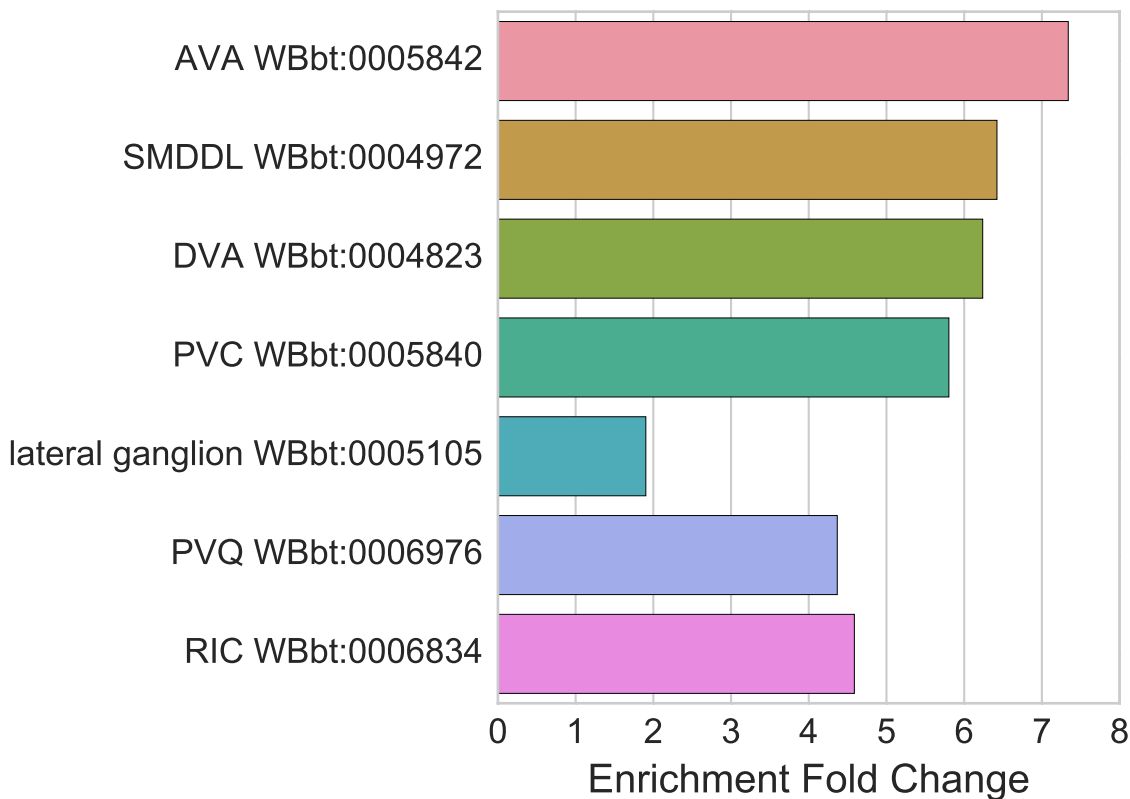

Supplement: Additional file 4 — Results. A folder containing a complete version of the results we generated for this paper. (ZIP 1597 kb) [file 12859_2016_1229_MOESM4_ESM.zip › output/HGT33_any_Results/WBPaper00037950_AVA-neuron_embryo_enriched_WBbt_0005842_534.pdf]

Tissue

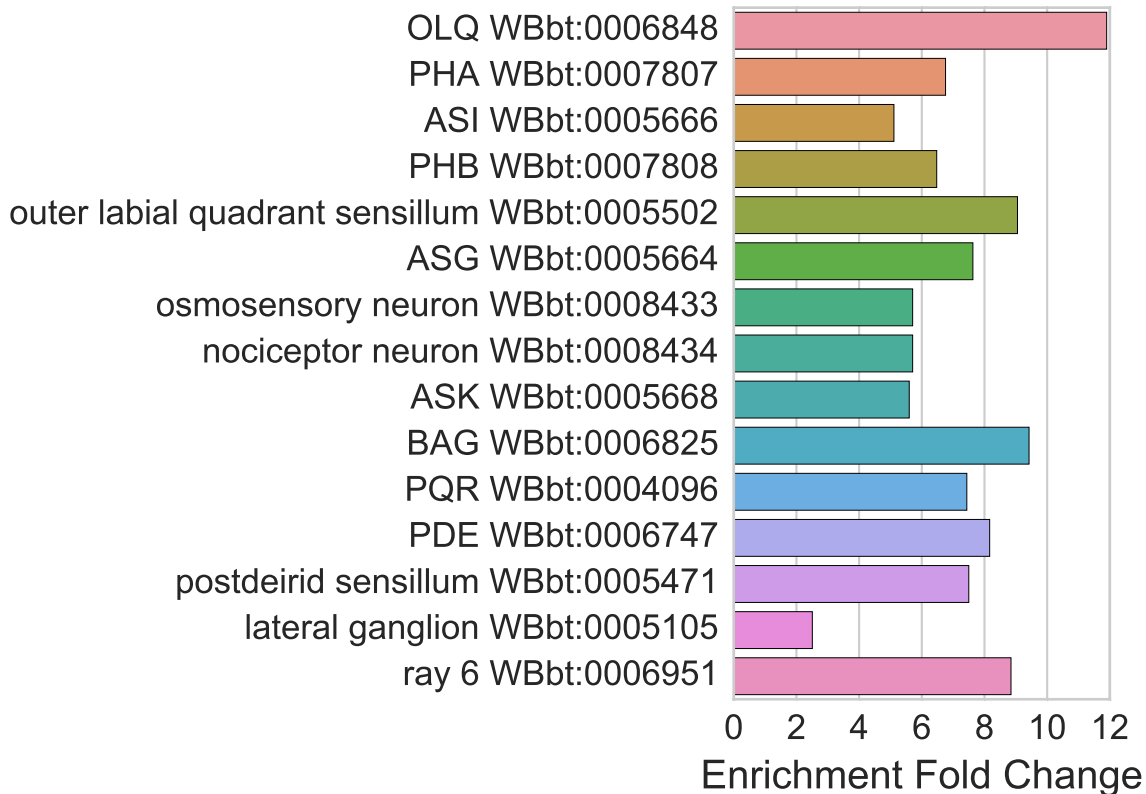

Supplement: Additional file 4 — Results. A folder containing a complete version of the results we generated for this paper. (ZIP 1597 kb) [file 12859_2016_1229_MOESM4_ESM.zip › output/HGT33_any_Results/WBPaper00037950_BAG-neuron_embryo_enriched_WBbt_0006825_454.pdf]

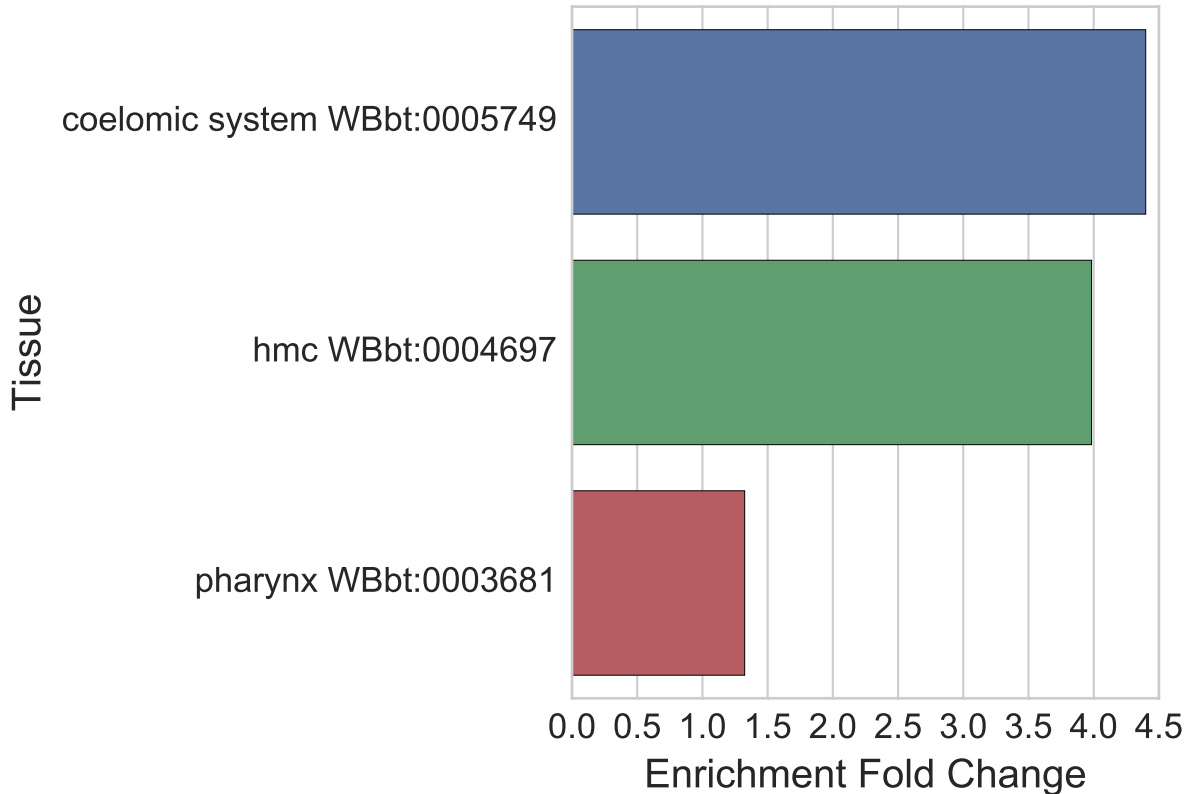

Supplement: Additional file 4 — Results. A folder containing a complete version of the results we generated for this paper. (ZIP 1597 kb) [file 12859_2016_1229_MOESM4_ESM.zip › output/HGT33_any_Results/WBPaper00037950_coelomocytes_embryo_enriched_WBbt_0005751_570.pdf]

Tissue

coelomic system WBbt:0005749

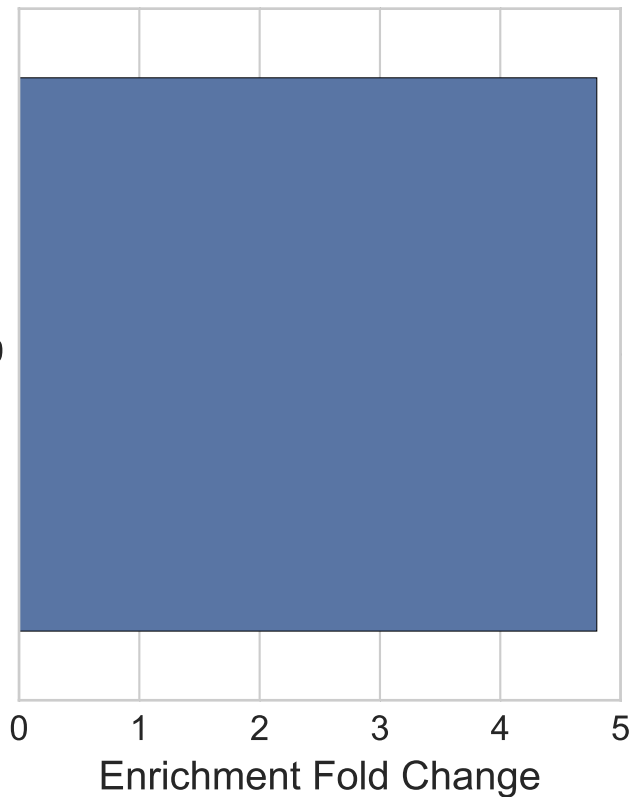

Supplement: Additional file 4 — Results. A folder containing a complete version of the results we generated for this paper. (ZIP 1597 kb) [file 12859_2016_1229_MOESM4_ESM.zip › output/HGT33_any_Results/WBPaper00037950_coelomocytes_larva_enriched_WBbt_0005751_229.pdf]

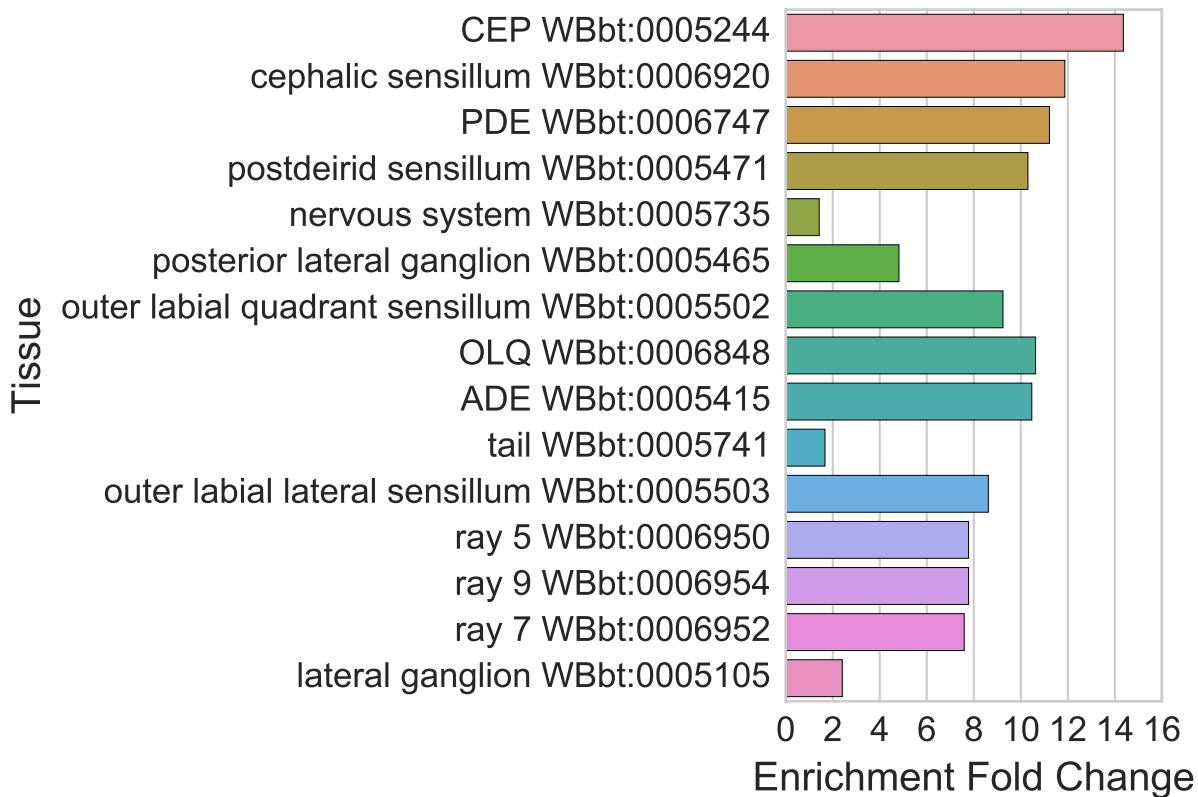

Supplement: Additional file 4 — Results. A folder containing a complete version of the results we generated for this paper. (ZIP 1597 kb) [file 12859_2016_1229_MOESM4_ESM.zip › output/HGT33_any_Results/WBPaper00037950_dopaminergic-neurons_embryo_enriched_WBbt_0006746_466.pdf]

Tissue

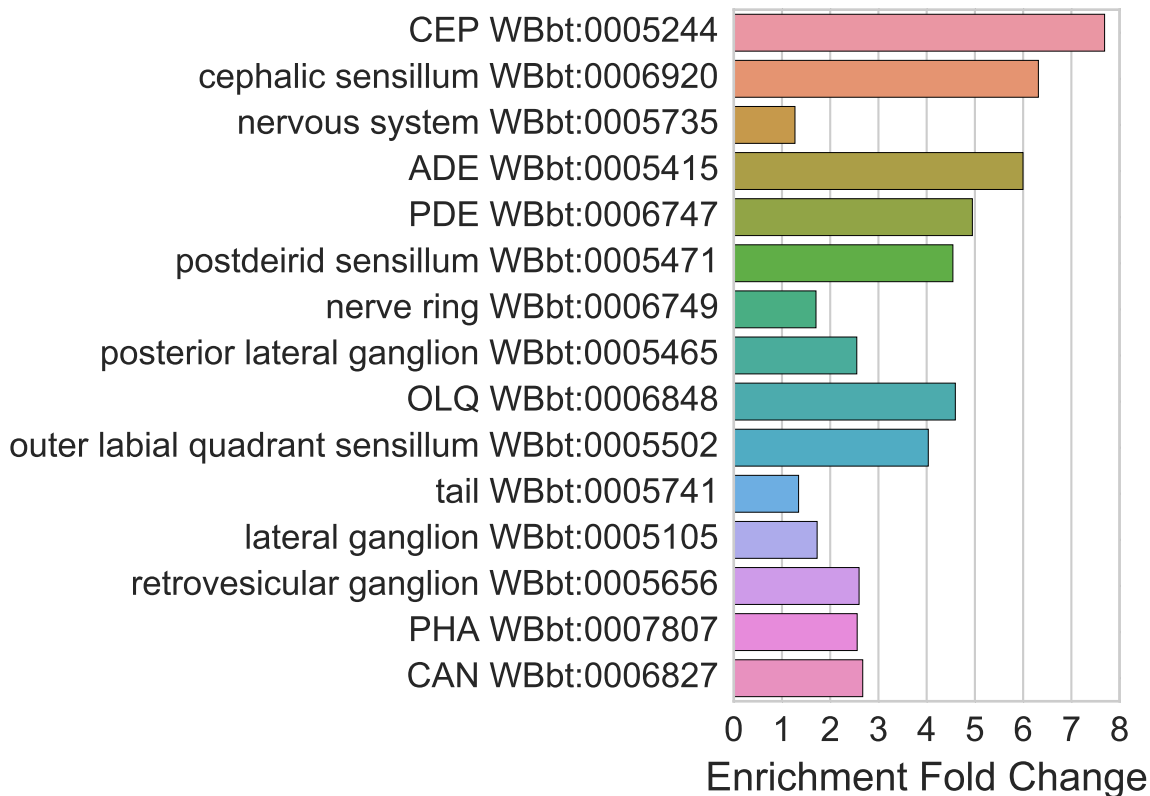

Supplement: Additional file 4 — Results. A folder containing a complete version of the results we generated for this paper. (ZIP 1597 kb) [file 12859_2016_1229_MOESM4_ESM.zip › output/HGT33_any_Results/WBPaper00037950_dopaminergic-neurons_larva_enriched_WBbt_0006746_1230.pdf]

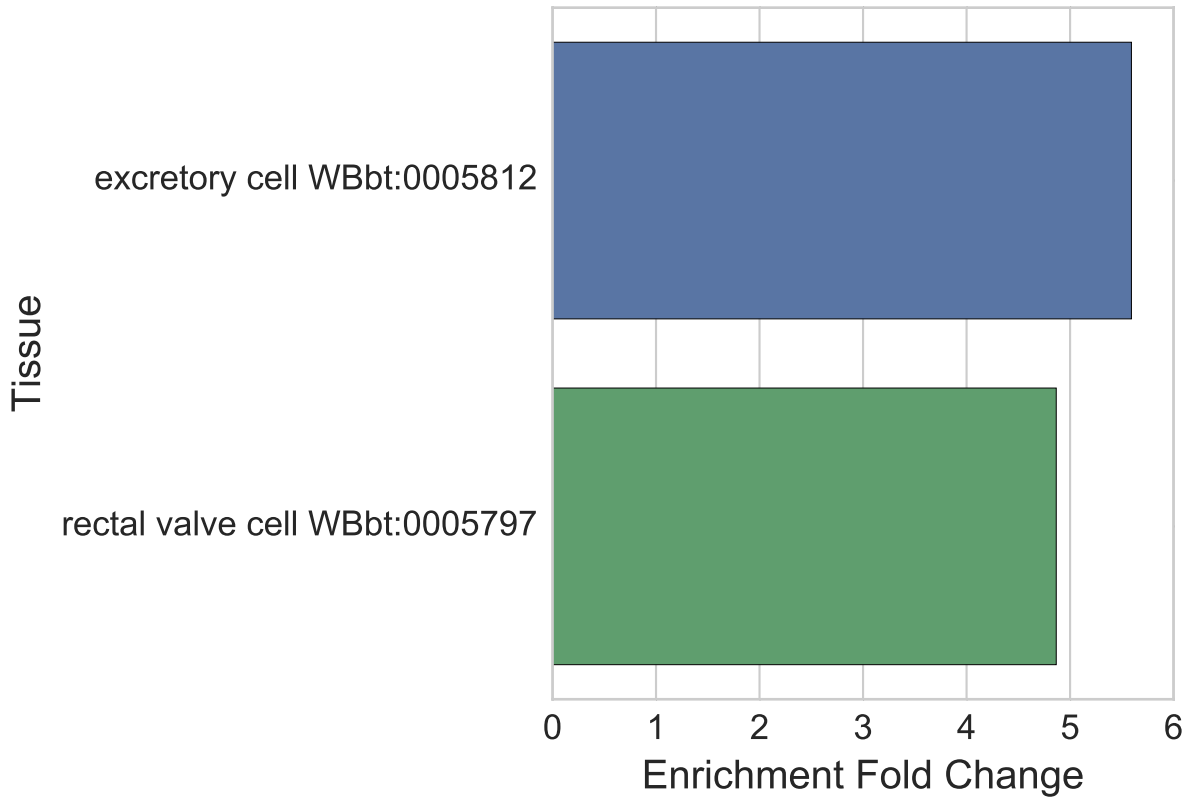

Supplement: Additional file 4 — Results. A folder containing a complete version of the results we generated for this paper. (ZIP 1597 kb) [file 12859_2016_1229_MOESM4_ESM.zip › output/HGT33_any_Results/WBPaper00037950_excretory-cell_larva_enriched_WBbt_0005812_528.pdf]

Tissue

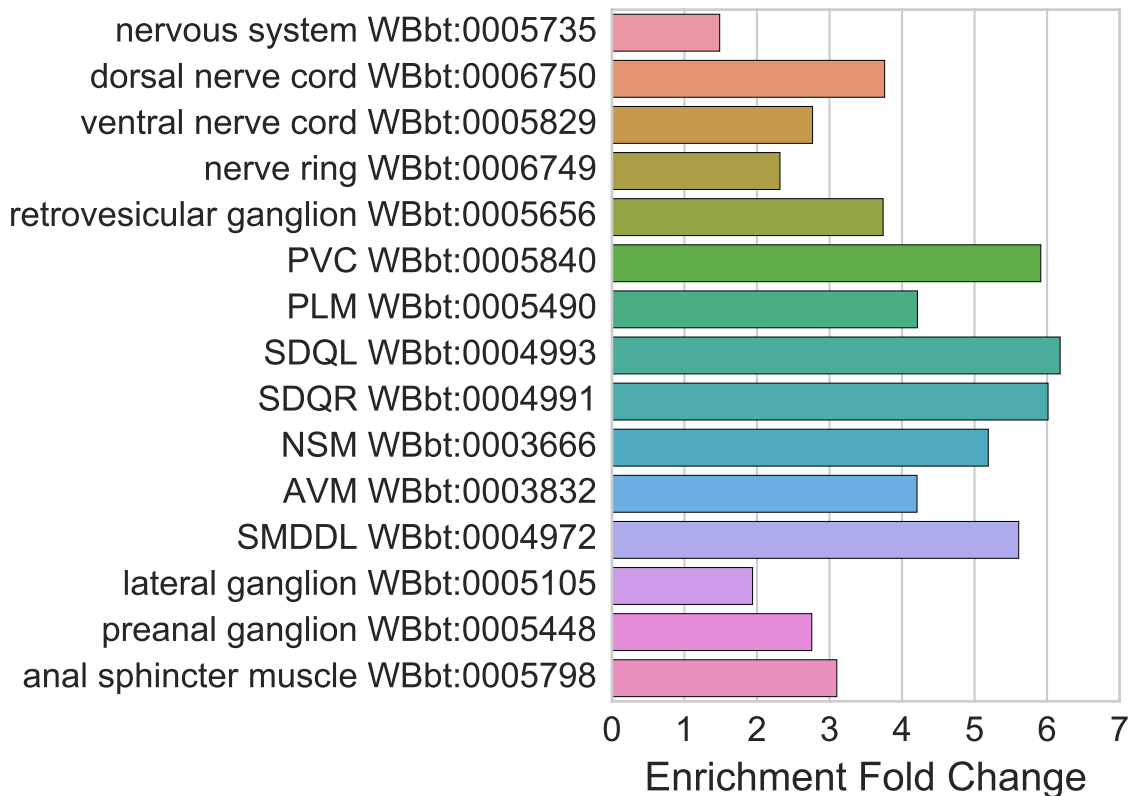

Supplement: Additional file 4 — Results. A folder containing a complete version of the results we generated for this paper. (ZIP 1597 kb) [file 12859_2016_1229_MOESM4_ESM.zip › output/HGT33_any_Results/WBPaper00037950_GABAergic-motor-neurons_embryo_enriched_WBbt_0005190_361.pdf]

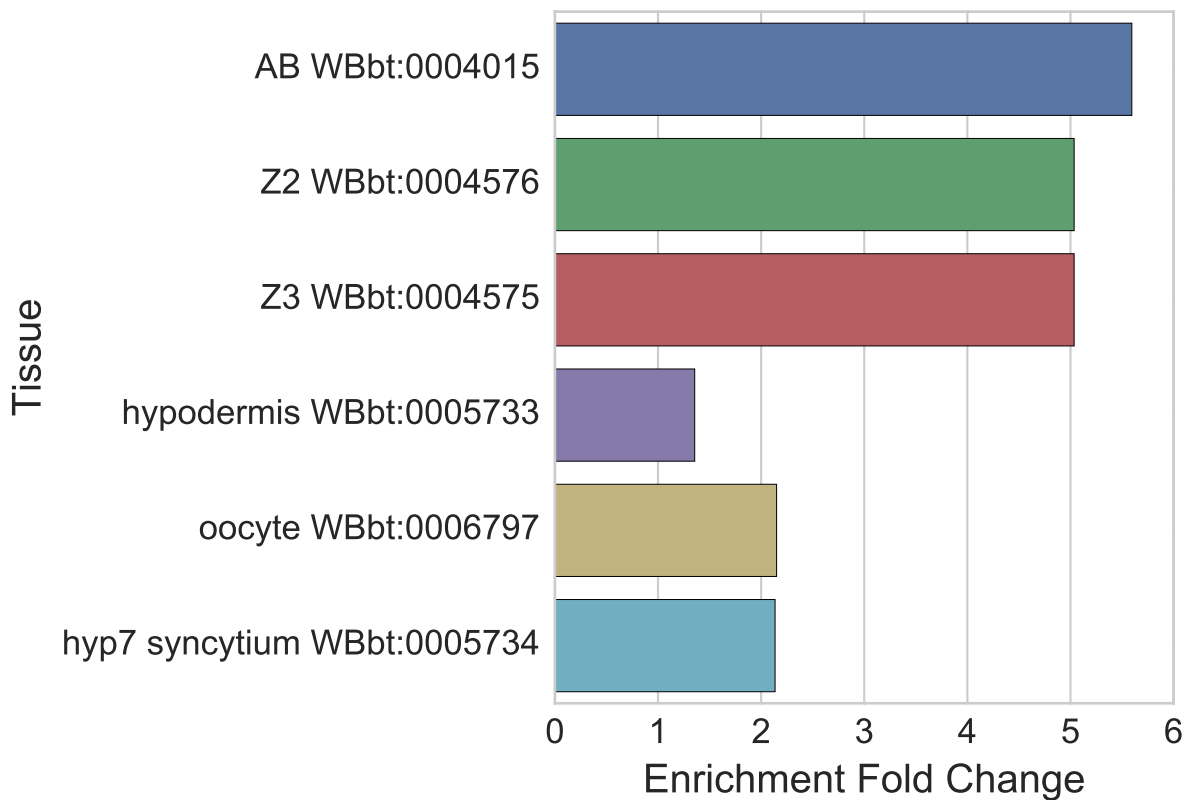

Supplement: Additional file 4 — Results. A folder containing a complete version of the results we generated for this paper. (ZIP 1597 kb) [file 12859_2016_1229_MOESM4_ESM.zip › output/HGT33_any_Results/WBPaper00037950_germline-precursors_embryo_enriched_WBbt_0006849_974.pdf]

Tissue

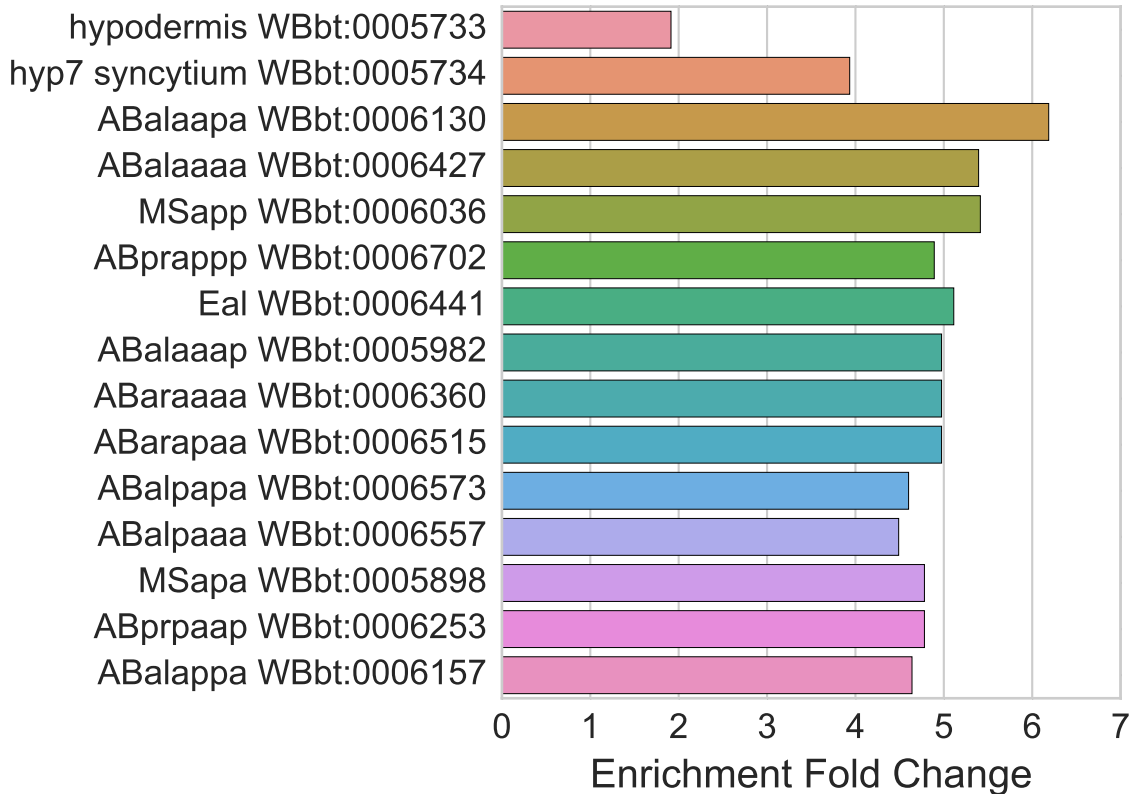

Supplement: Additional file 4 — Results. A folder containing a complete version of the results we generated for this paper. (ZIP 1597 kb) [file 12859_2016_1229_MOESM4_ESM.zip › output/HGT33_any_Results/WBPaper00037950_hypodermis_embryo_enriched_WBbt_0005733_734.pdf]

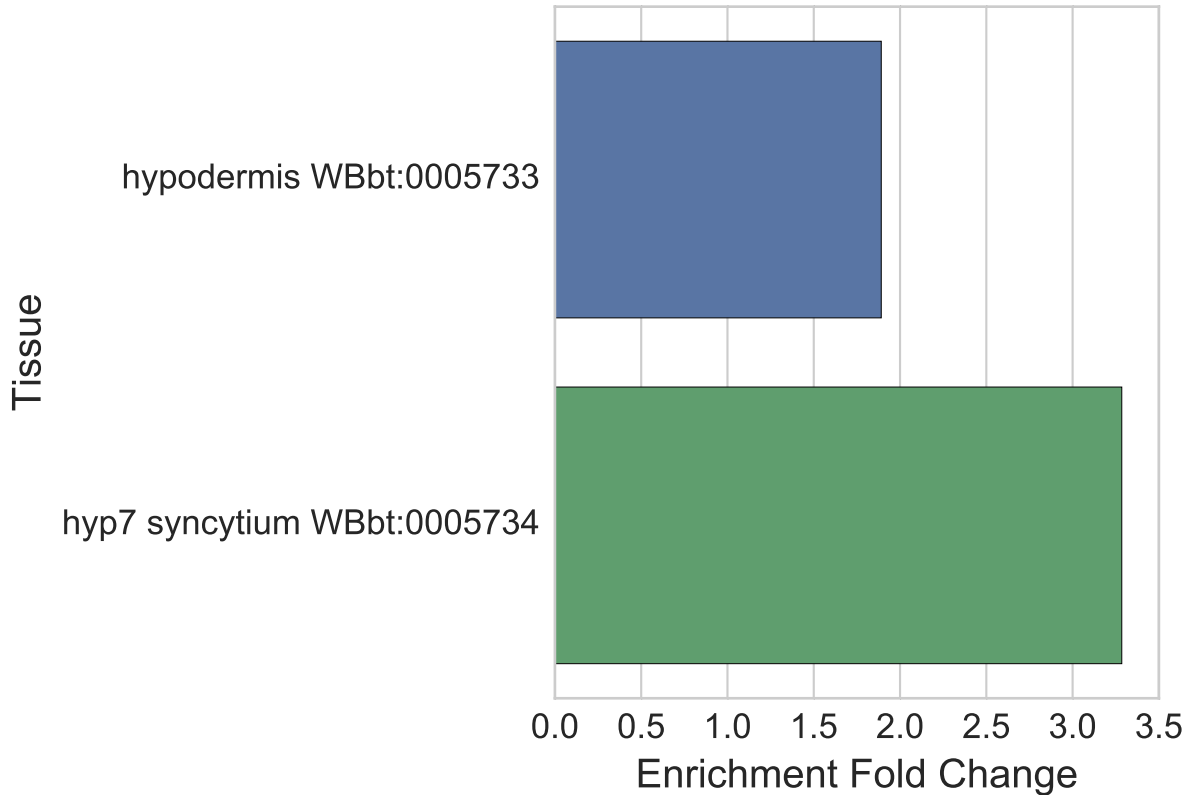

Supplement: Additional file 4 — Results. A folder containing a complete version of the results we generated for this paper. (ZIP 1597 kb) [file 12859_2016_1229_MOESM4_ESM.zip › output/HGT33_any_Results/WBPaper00037950_hypodermis_larva_enriched_WBbt_0005733_1250.pdf]

Tissue

PVD WBbt:0006831

0.0 0.5 1.0 1.5 2.0 2.5 3.0 3.5

Enrichment Fold Change

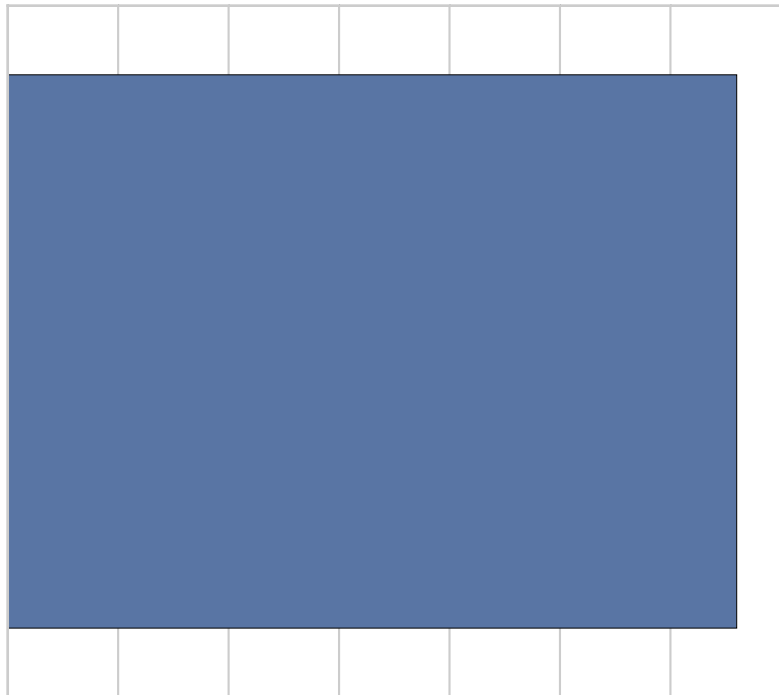

Supplement: Additional file 4 — Results. A folder containing a complete version of the results we generated for this paper. (ZIP 1597 kb) [file 12859_2016_1229_MOESM4_ESM.zip › output/HGT33_any_Results/WBPaper00037950_PVD-OLL-neurons_larva_enriched_WBbt_0006831_878.pdf]

Tissue

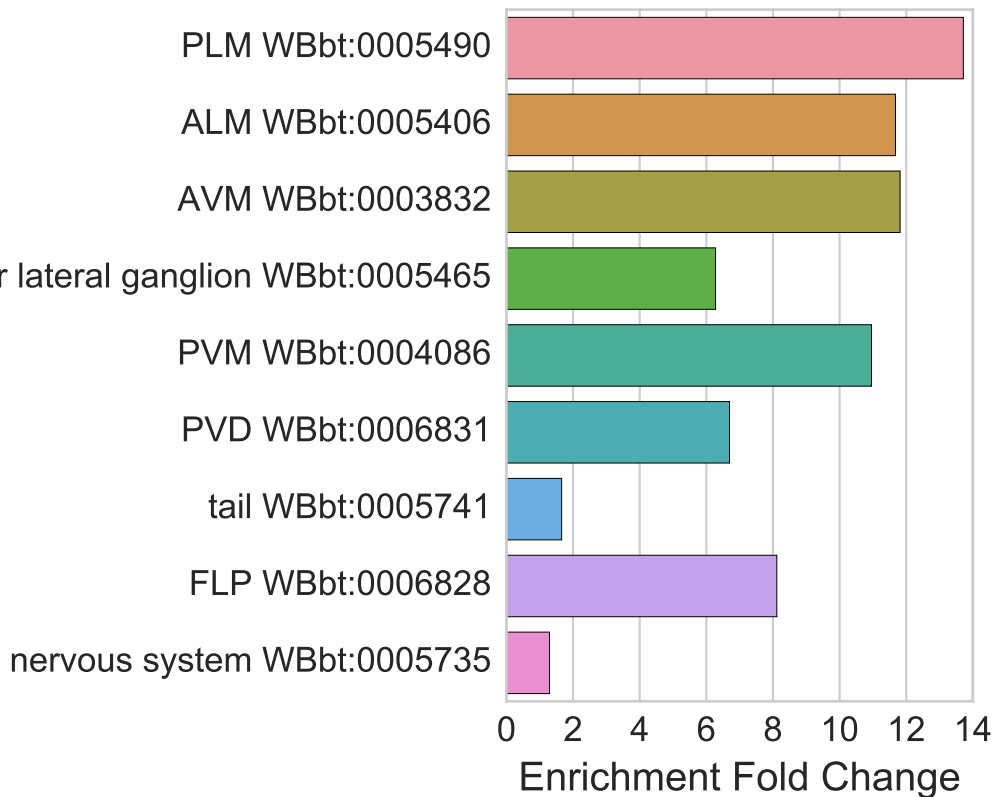

Supplement: Additional file 4 — Results. A folder containing a complete version of the results we generated for this paper. (ZIP 1597 kb) [file 12859_2016_1229_MOESM4_ESM.zip › output/HGT33_any_Results/WBPaper00040420_ALM_PLM_enriched_WBbt_0005406_198.pdf]

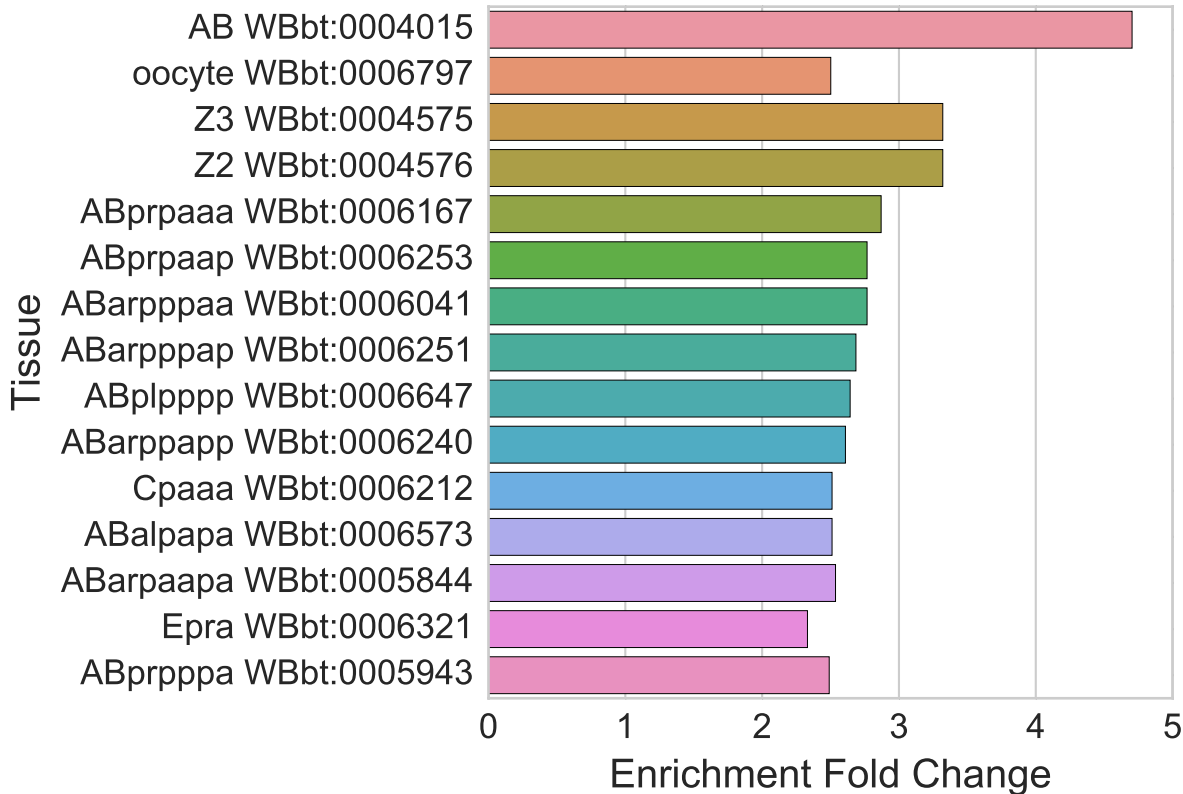

Supplement: Additional file 4 — Results. A folder containing a complete version of the results we generated for this paper. (ZIP 1597 kb) [file 12859_2016_1229_MOESM4_ESM.zip › output/HGT33_any_Results/WBPaper00044760_germline_specific_WBbt_0005784_2510.pdf]

Tissue

oocyte WBbt:0006797

gonadal sheath cell WBbt:0005828

spermatheca WBbt:0005319

0.0 0.5 1.0 1.5 2.0 2.5 3.0  
Enrichment Fold Change

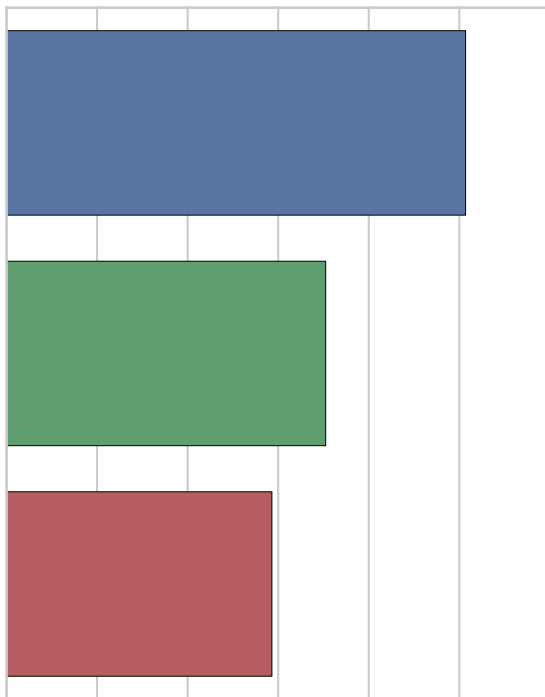

Supplement: Additional file 4 — Results. A folder containing a complete version of the results we generated for this paper. (ZIP 1597 kb) [file 12859_2016_1229_MOESM4_ESM.zip › output/HGT33_any_Results/WBPaper00045521_Spermatogenic_WBbt_0005784_2743.pdf]

Tissue

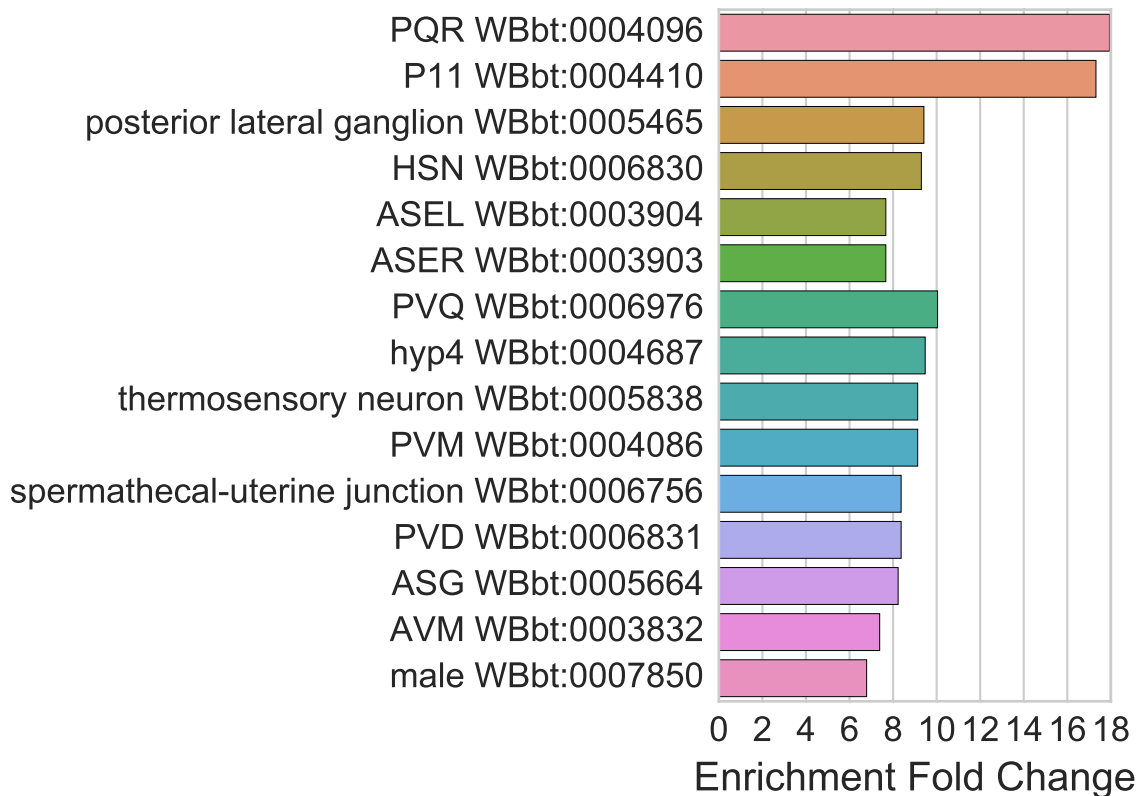

Supplement: Additional file 4 — Results. A folder containing a complete version of the results we generated for this paper. (ZIP 1597 kb) [file 12859_2016_1229_MOESM4_ESM.zip › output/HGT50_any_Results/WBPaper00013489_Ray_Enriched_WBbt_0006941_25.pdf]

Tissue

pharynx WBbt:0003681

0.0 0.2 0.4 0.6 0.8 1.0 1.2 1.4 1.6 1.8

Enrichment Fold Change

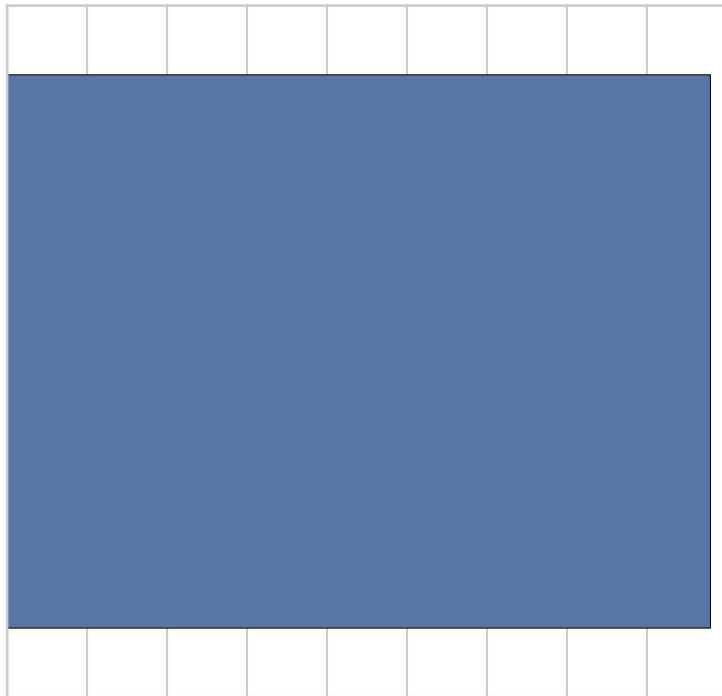

Supplement: Additional file 4 — Results. A folder containing a complete version of the results we generated for this paper. (ZIP 1597 kb) [file 12859_2016_1229_MOESM4_ESM.zip › output/HGT50_any_Results/WBPaper00024505_pharyngeal_enriched_WBbt_0003681_329.pdf]

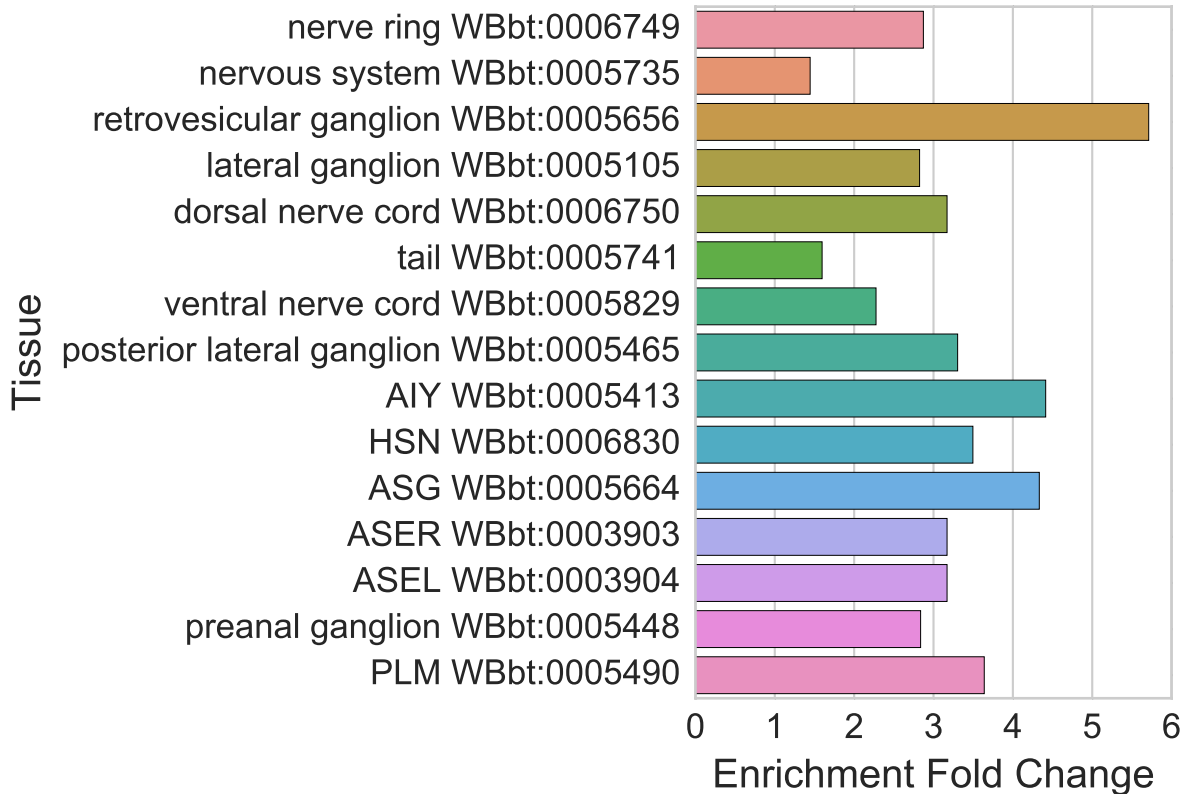

Supplement: Additional file 4 — Results. A folder containing a complete version of the results we generated for this paper. (ZIP 1597 kb) [file 12859_2016_1229_MOESM4_ESM.zip › output/HGT50_any_Results/WBPaper00024970_GABAergic_neuron_specific_WBbt_0005190_247.pdf]

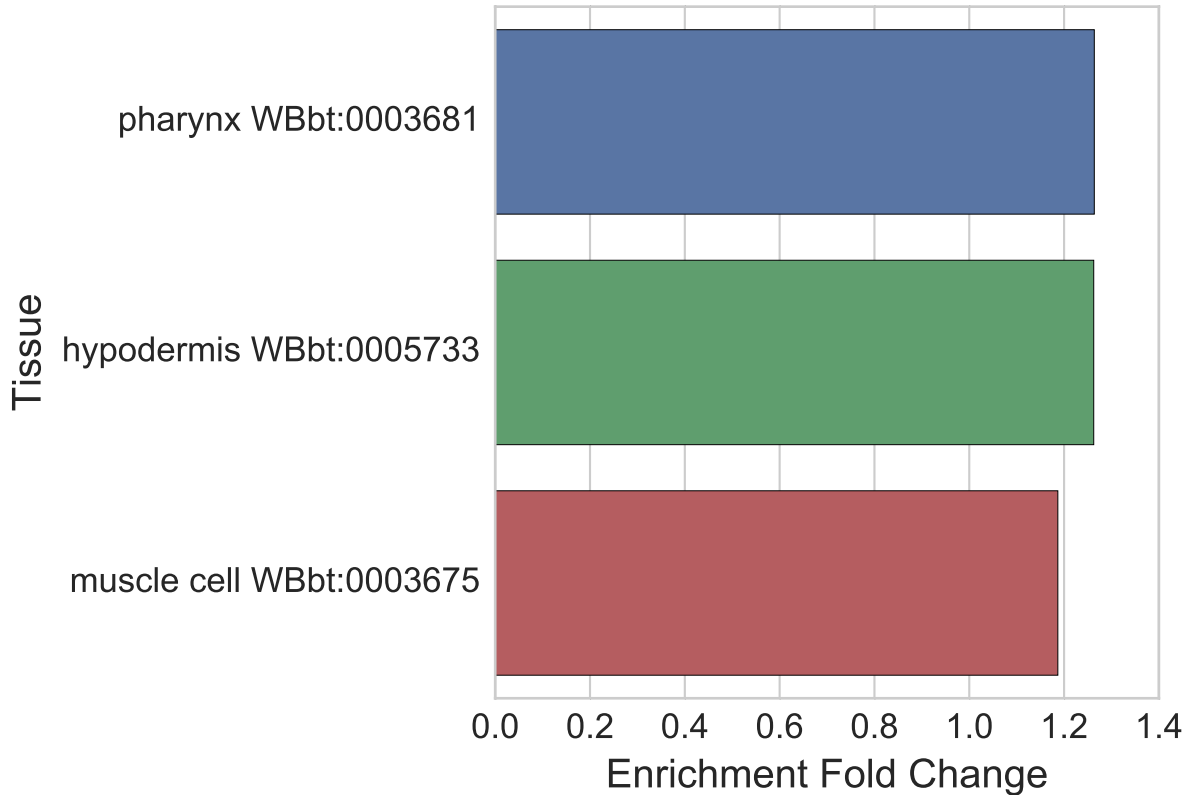

Supplement: Additional file 4 — Results. A folder containing a complete version of the results we generated for this paper. (ZIP 1597 kb) [file 12859_2016_1229_MOESM4_ESM.zip › output/HGT50_any_Results/WBPaper00026980_intestine_enriched_WBbt_0005772_1970.pdf]

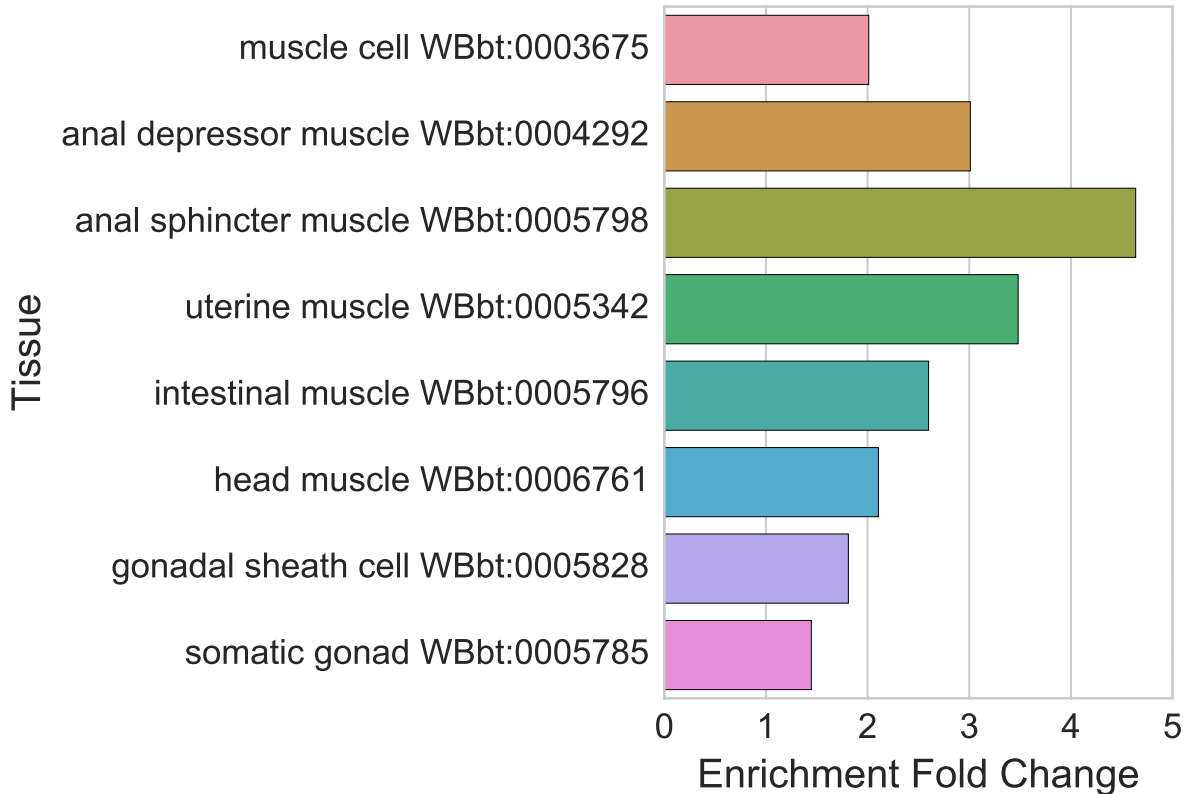

Supplement: Additional file 4 — Results. A folder containing a complete version of the results we generated for this paper. (ZIP 1597 kb) [file 12859_2016_1229_MOESM4_ESM.zip › output/HGT50_any_Results/WBPaper00031003_0hr_muscle_enriched_WBbt_0003675_761.pdf]

Tissue

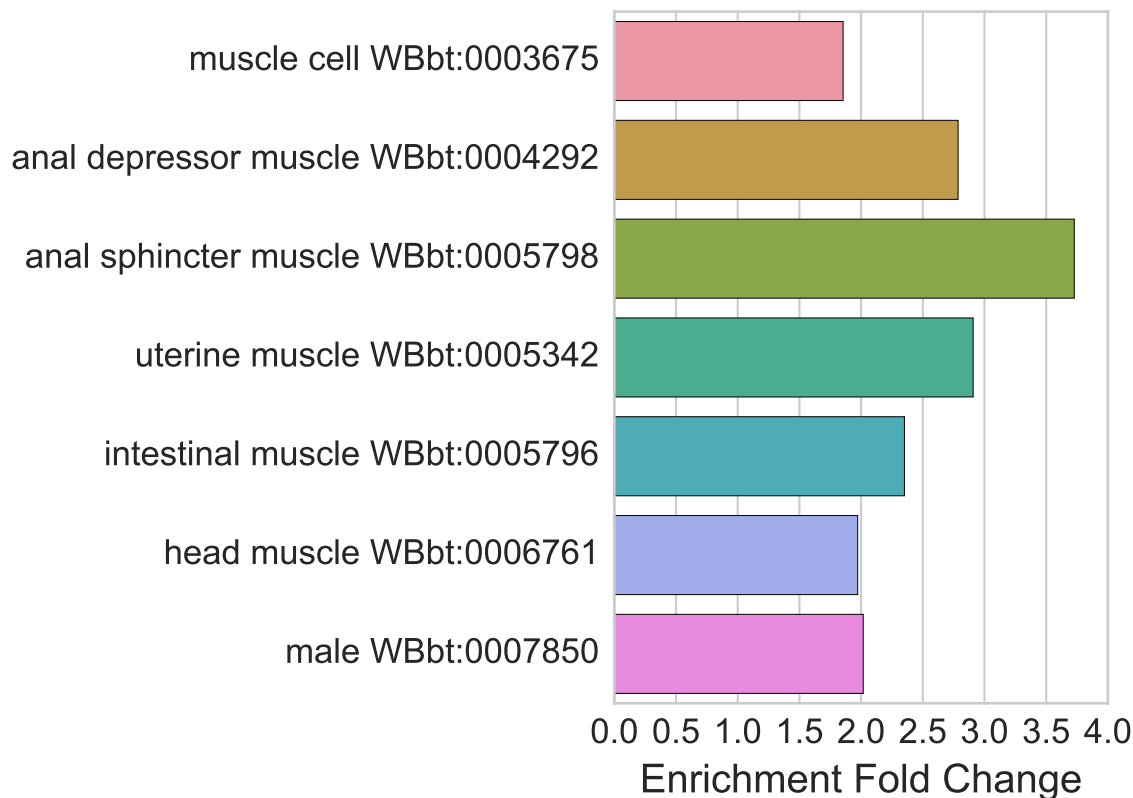

Supplement: Additional file 4 — Results. A folder containing a complete version of the results we generated for this paper. (ZIP 1597 kb) [file 12859_2016_1229_MOESM4_ESM.zip › output/HGT50_any_Results/WBPaper00031003_total_muscle_enriched_WBbt_0003675_1285.pdf]

Tissue

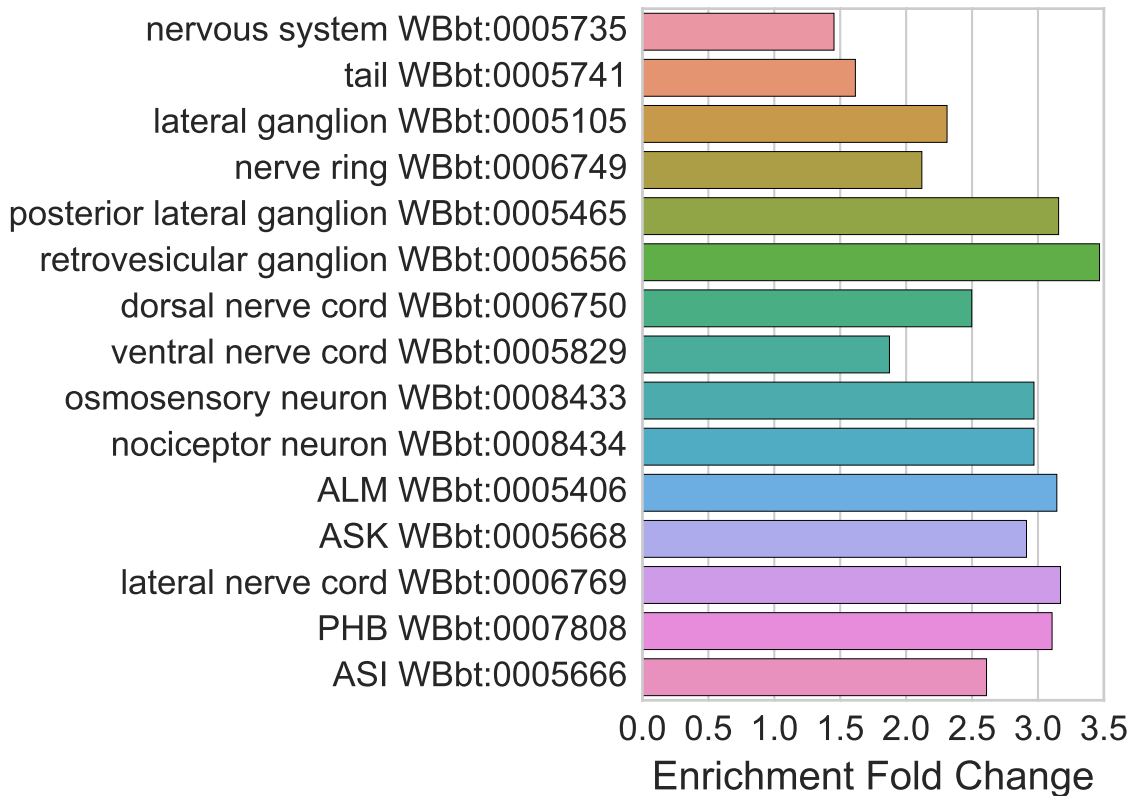

Supplement: Additional file 4 — Results. A folder containing a complete version of the results we generated for this paper. (ZIP 1597 kb) [file 12859_2016_1229_MOESM4_ESM.zip › output/HGT50_any_Results/WBPaper00031532_Larva_Pan_Neuronal_Enriched_WBbt_0003679_1603.pdf]

Tissue

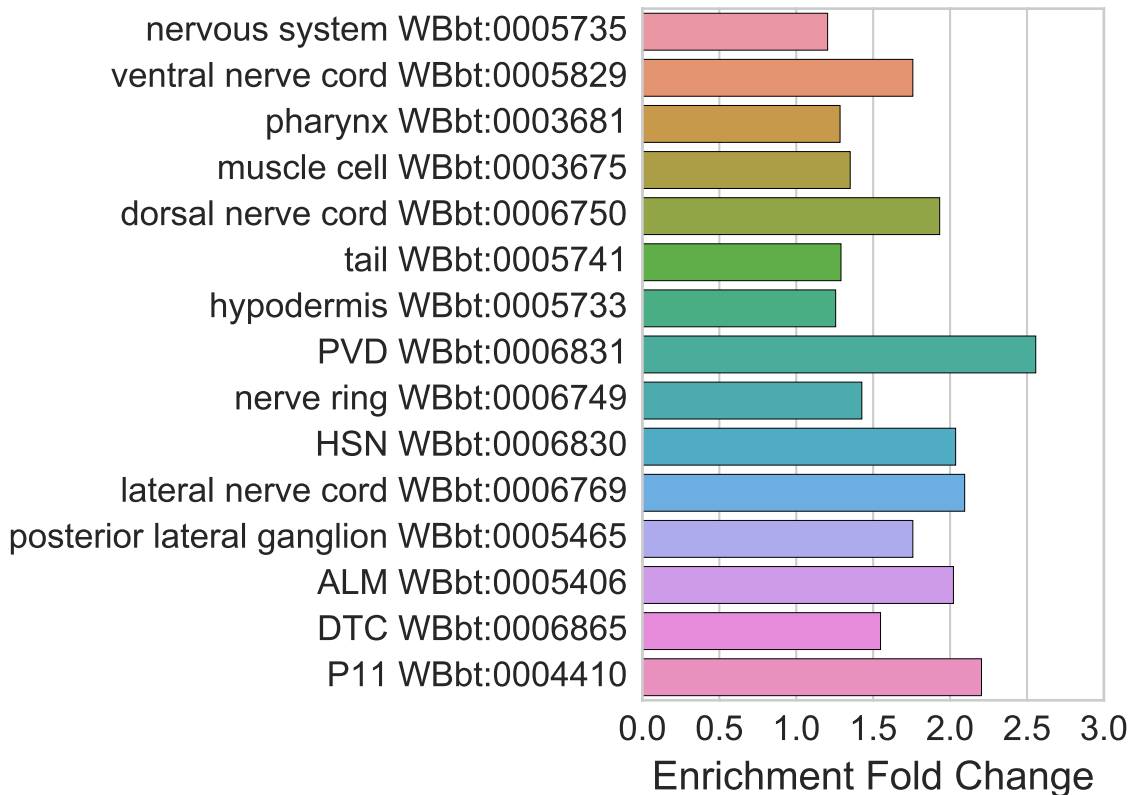

Supplement: Additional file 4 — Results. A folder containing a complete version of the results we generated for this paper. (ZIP 1597 kb) [file 12859_2016_1229_MOESM4_ESM.zip › output/HGT50_any_Results/WBPaper00036375_enriched_in_PVD_OLL_WBbt_0006831_2180.pdf]

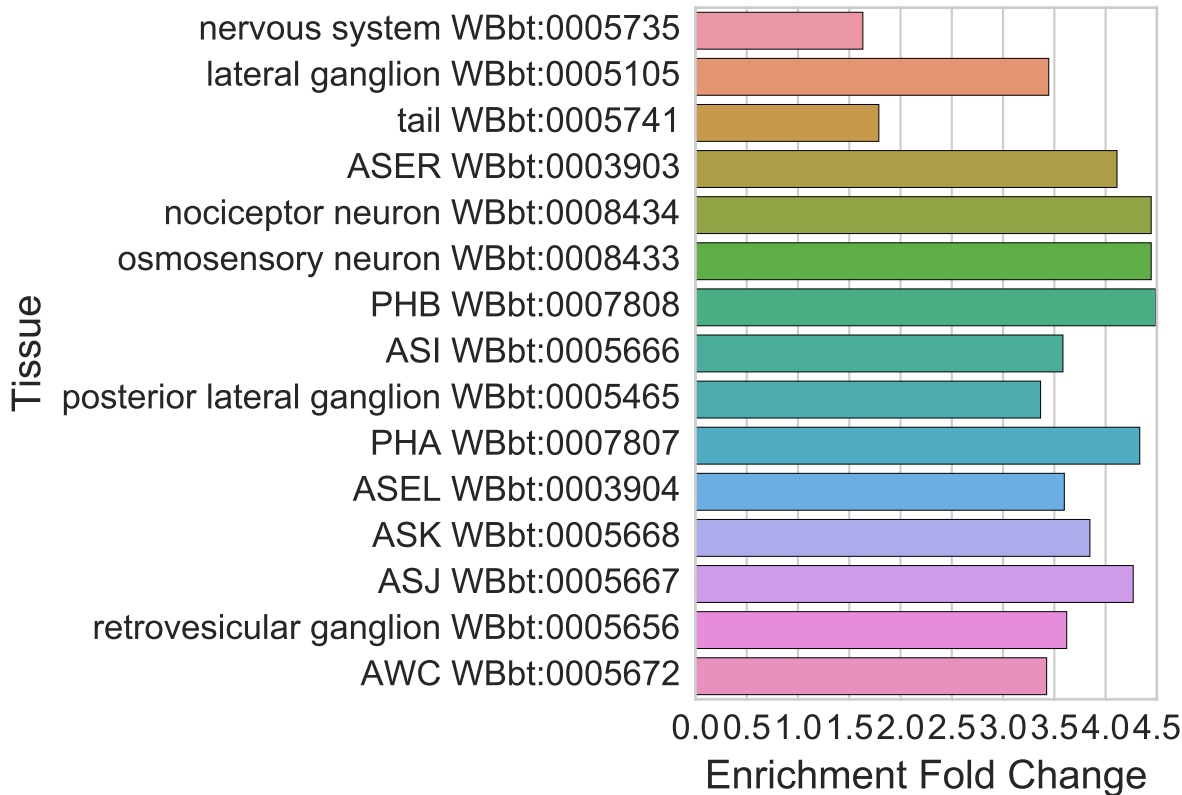

Supplement: Additional file 4 — Results. A folder containing a complete version of the results we generated for this paper. (ZIP 1597 kb) [file 12859_2016_1229_MOESM4_ESM.zip › output/HGT50_any_Results/WBPaper00037950_all-neurons_larva_enriched_WBbt_0003679_1013.pdf]

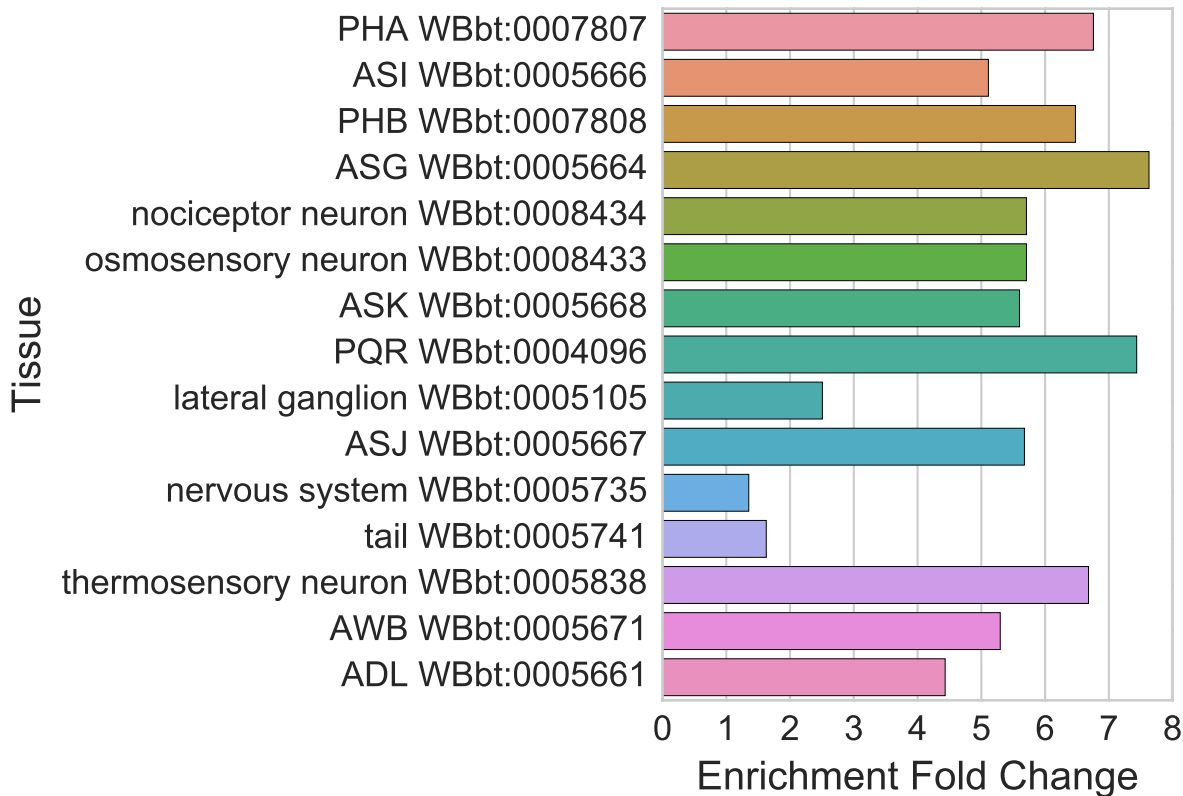

Supplement: Additional file 4 — Results. A folder containing a complete version of the results we generated for this paper. (ZIP 1597 kb) [file 12859_2016_1229_MOESM4_ESM.zip › output/HGT50_any_Results/WBPaper00037950_BAG-neuron_embryo_enriched_WBbt_0006825_454.pdf]

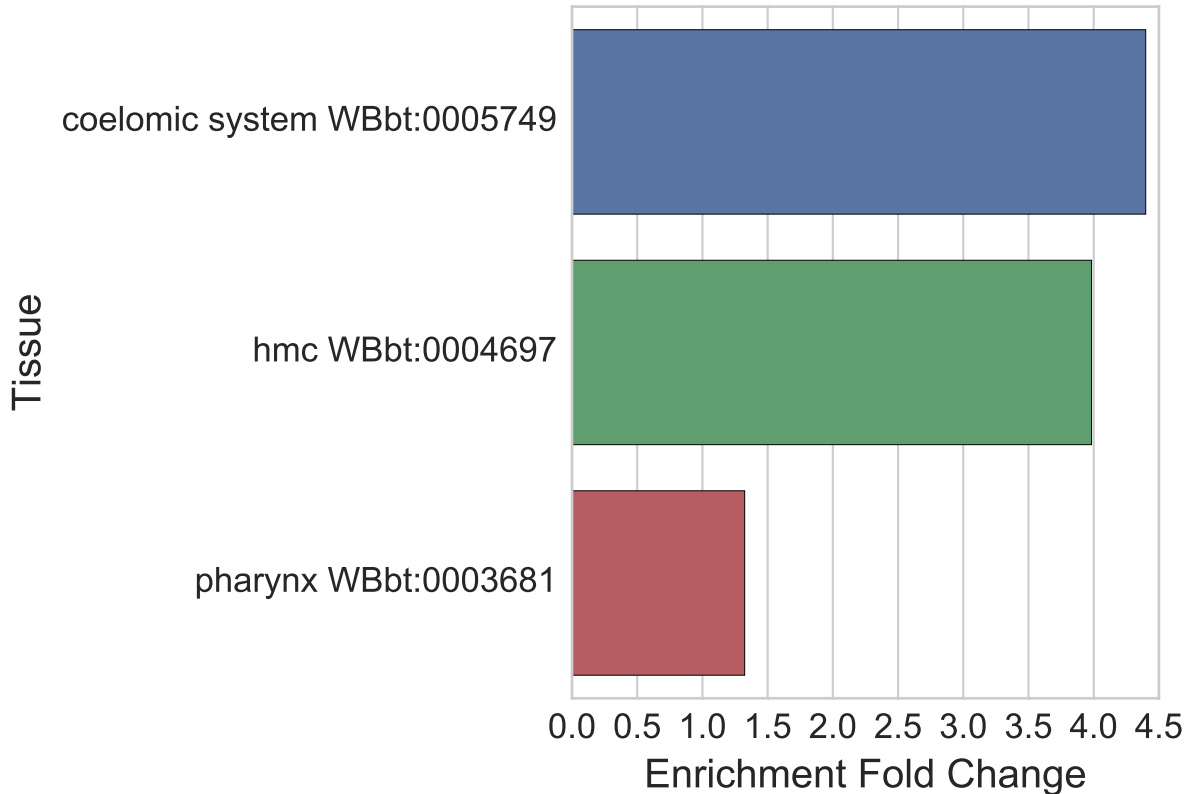

Supplement: Additional file 4 — Results. A folder containing a complete version of the results we generated for this paper. (ZIP 1597 kb) [file 12859_2016_1229_MOESM4_ESM.zip › output/HGT50_any_Results/WBPaper00037950_coelomocytes_embryo_enriched_WBbt_0005751_570.pdf]
