# Supplementary material for: Tissue enrichment analysis for C. elegans genomics
Source: BMC Bioinformatics. 2016 Sep 13;17(1):366. doi: 10.1186/s12859-016-1229-9 (PMC5020436; doi:10.1186/s12859-016-1229-9)
Supplement: Additional file 4 — Results. A folder containing a complete version of the results we generated for this paper. (ZIP 1597 kb) [file 12859_2016_1229_MOESM4_ESM.zip › output/HGT50_any_Results/WBPaper00037950_dopaminergic-neurons_embryo_enriched_WBbt_0006746_466.pdf]

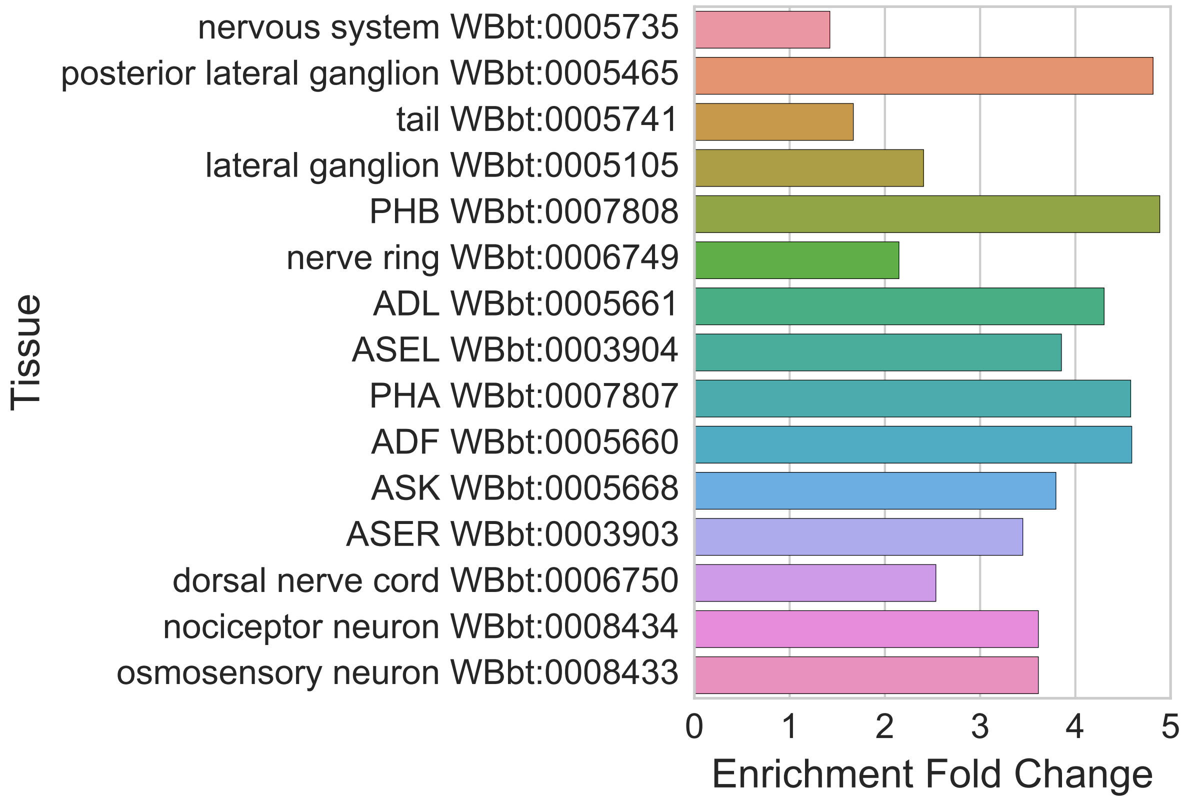

Tissue
nervous system WBbt:0005735
posterior lateral ganglion WBbt:0005465
tail WBbt:0005741
lateral ganglion WBbt:0005105
PHB WBbt:0007808
nerve ring WBbt:0006749
ADL WBbt:0005661
ASEL WBbt:0003904
PHA WBbt:0007807
ADF WBbt:0005660
ASK WBbt:0005668
ASER WBbt:0003903
dorsal nerve cord WBbt:0006750
nociceptor neuron WBbt:0008434
osmosensory neuron WBbt:0008433
0
1
2
3
4
5
Enrichment Fold Change
