# Supplementary material for: Tissue enrichment analysis for C. elegans genomics
Source: BMC Bioinformatics. 2016 Sep 13;17(1):366. doi: 10.1186/s12859-016-1229-9 (PMC5020436; doi:10.1186/s12859-016-1229-9)
Supplement: Additional file 4 — Results. A folder containing a complete version of the results we generated for this paper. (ZIP 1597 kb) [file 12859_2016_1229_MOESM4_ESM.zip › output/HGT50_any_Results/WBPaper00037950_dopaminergic-neurons_larva_enriched_WBbt_0006746_1230.pdf]

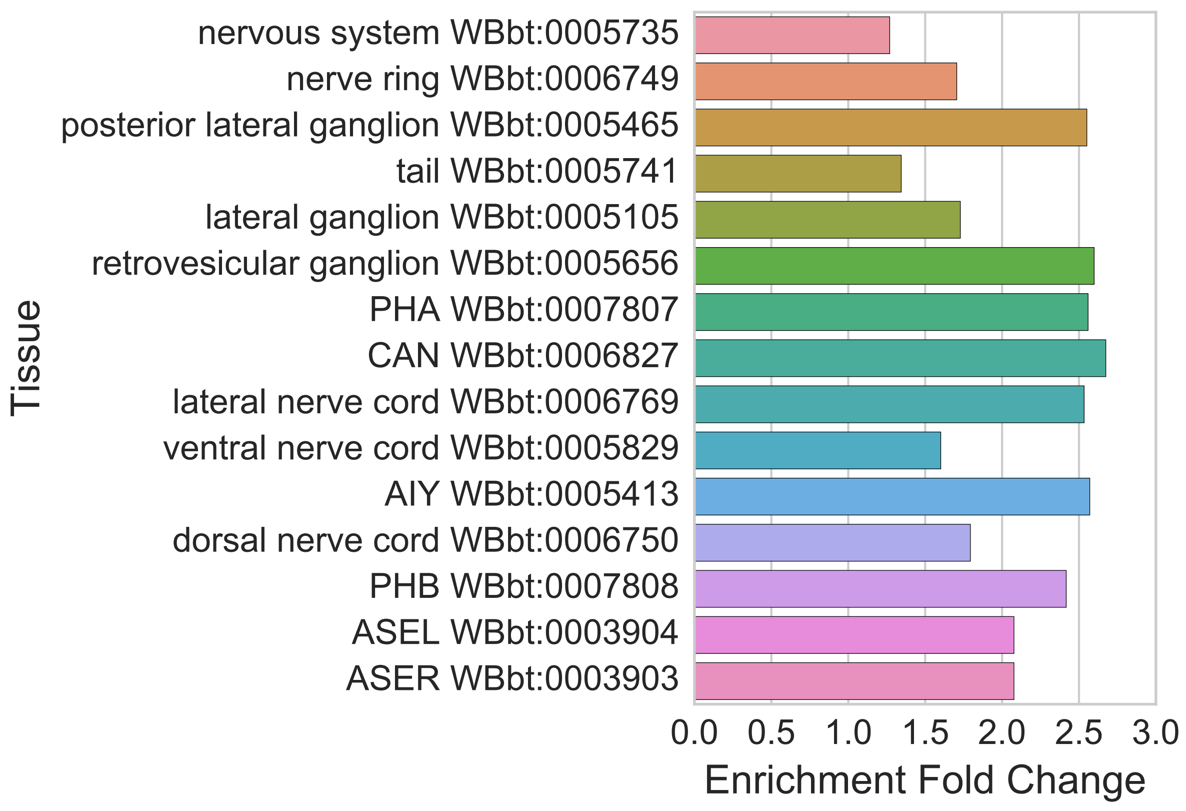
nervous system WBbt:0005735
nerve ring WBbt:0006749
posterior lateral ganglion WBbt:0005465
tail WBbt:0005741
lateral ganglion WBbt:0005105
retrovesicular ganglion WBbt:0005656
PHA WBbt:0007807
CAN WBbt:0006827
lateral nerve cord WBbt:0006769
ventral nerve cord WBbt:0005829
AIY WBbt:0005413
dorsal nerve cord WBbt:0006750
PHB WBbt:0007808
ASEL WBbt:0003904
ASER WBbt:0003903
Tissue
0.0
0.5
1.0
1.5
2.0
2.5
3.0
Enrichment Fold Change
