# Supplementary material for: Tissue enrichment analysis for C. elegans genomics
Source: BMC Bioinformatics. 2016 Sep 13;17(1):366. doi: 10.1186/s12859-016-1229-9 (PMC5020436; doi:10.1186/s12859-016-1229-9)
Supplement: Additional file 4 — Results. A folder containing a complete version of the results we generated for this paper. (ZIP 1597 kb) [file 12859_2016_1229_MOESM4_ESM.zip › output/HGT50_any_Results/WBPaper00037950_GABAergic-motor-neurons_embryo_enriched_WBbt_0005190_361.pdf]

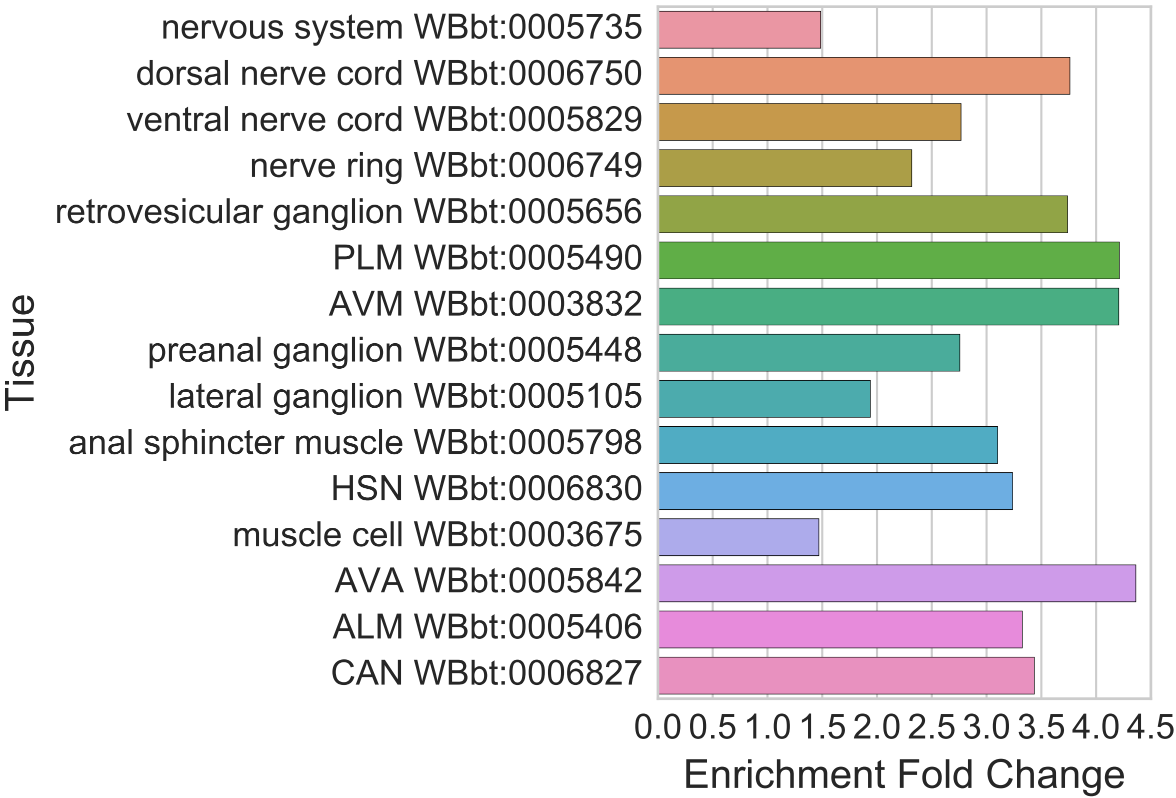
Tissue
nervous system WBbt:0005735
dorsal nerve cord WBbt:0006750
ventral nerve cord WBbt:0005829
nerve ring WBbt:0006749
retrovesicular ganglion WBbt:0005656
PLM WBbt:0005490
AVM WBbt:0003832
preanal ganglion WBbt:0005448
lateral ganglion WBbt:0005105
anal sphincter muscle WBbt:0005798
HSN WBbt:0006830
muscle cell WBbt:0003675
AVA WBbt:0005842
ALM WBbt:0005406
CAN WBbt:0006827
0.0 0.5 1.0 1.5 2.0 2.5 3.0 3.5 4.0 4.5
Enrichment Fold Change
