# Supplementary material for: Tissue enrichment analysis for C. elegans genomics
Source: BMC Bioinformatics. 2016 Sep 13;17(1):366. doi: 10.1186/s12859-016-1229-9 (PMC5020436; doi:10.1186/s12859-016-1229-9)
Supplement: Additional file 4 — Results. A folder containing a complete version of the results we generated for this paper. (ZIP 1597 kb) [file 12859_2016_1229_MOESM4_ESM.zip › output/HGT50_any_Results/WBPaper00037950_hypodermis_larva_enriched_WBbt_0005733_1250.pdf]

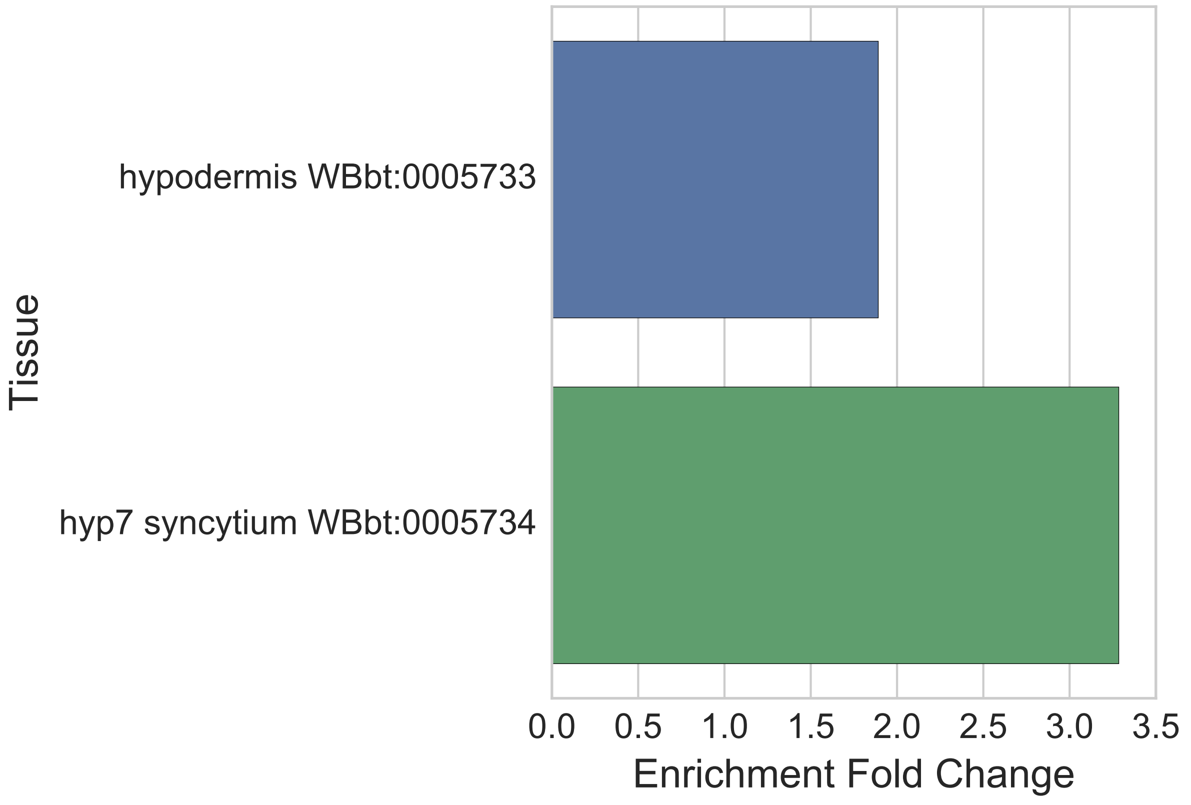

hypodermis WBbt:0005733
hyp7 syncytium WBbt:0005734
Tissue
0.0
0.5
1.0
1.5
2.0
2.5
3.0
3.5
Enrichment Fold Change
