# Supplementary material for: Tissue enrichment analysis for C. elegans genomics
Source: BMC Bioinformatics. 2016 Sep 13;17(1):366. doi: 10.1186/s12859-016-1229-9 (PMC5020436; doi:10.1186/s12859-016-1229-9)
Supplement: Additional file 4 — Results. A folder containing a complete version of the results we generated for this paper. (ZIP 1597 kb) [file 12859_2016_1229_MOESM4_ESM.zip › output/HGT50_any_Results/WBPaper00040420_FLP_enriched_WBbt_0006828_288.pdf]

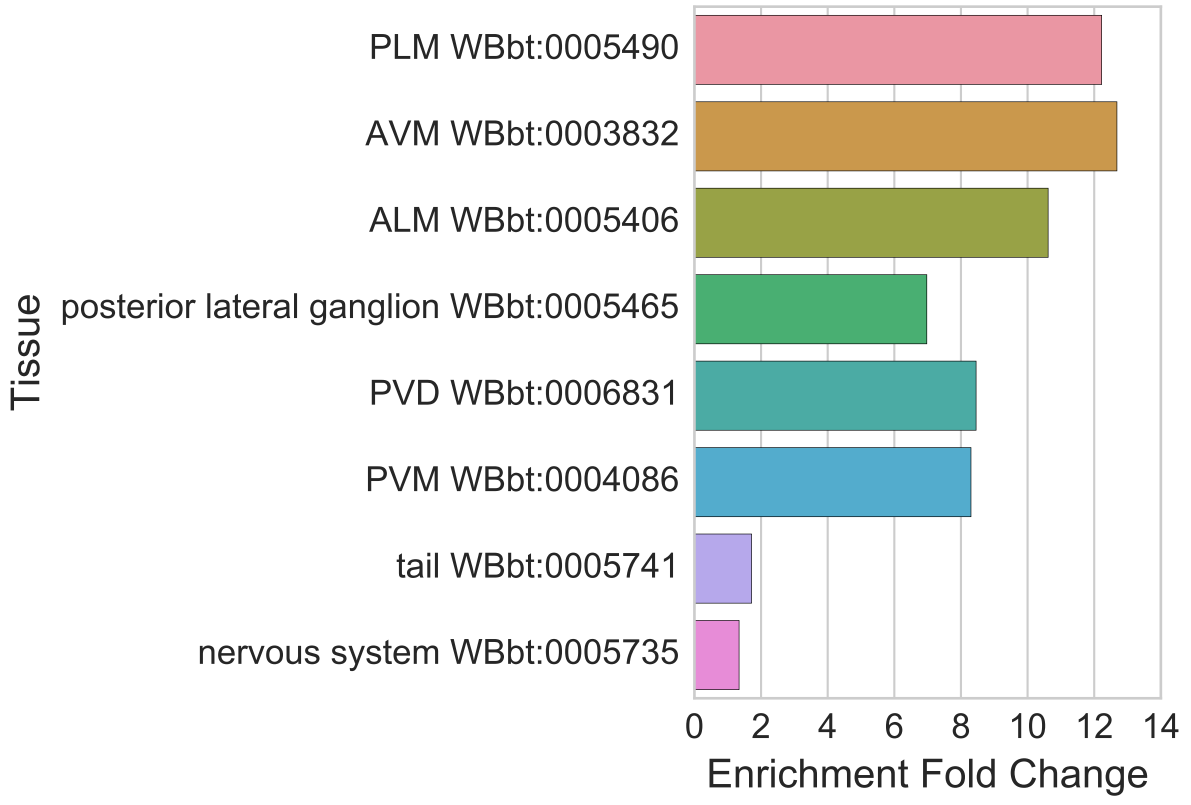

Tissue
PLM WBbt:0005490
AVM WBbt:0003832
ALM WBbt:0005406
posterior lateral ganglion WBbt:0005465
PVD WBbt:0006831
PVM WBbt:0004086
tail WBbt:0005741
nervous system WBbt:0005735
0
2
4
6
8
10
12
14
Enrichment Fold Change
