# Supplementary material for: Tissue enrichment analysis for C. elegans genomics
Source: BMC Bioinformatics. 2016 Sep 13;17(1):366. doi: 10.1186/s12859-016-1229-9 (PMC5020436; doi:10.1186/s12859-016-1229-9)
Supplement: Additional file 4 — Results. A folder containing a complete version of the results we generated for this paper. (ZIP 1597 kb) [file 12859_2016_1229_MOESM4_ESM.zip › output/SummaryInformation/avgQKDE_method=avg.pdf]

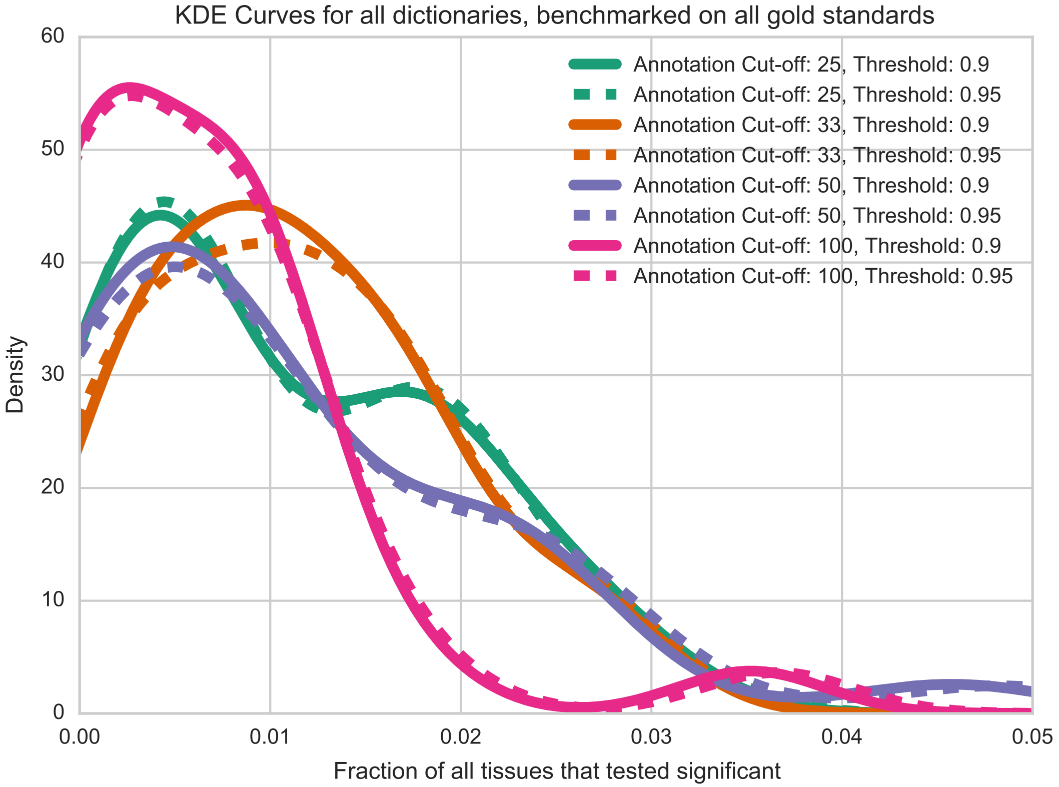

KDE Curves for all dictionaries, benchmarked on all gold standards
Annotation Cut-off: 25, Threshold: 0.9
Annotation Cut-off: 25, Threshold: 0.95
Annotation Cut-off: 33, Threshold: 0.9
Annotation Cut-off: 33, Threshold: 0.95
Annotation Cut-off: 50, Threshold: 0.9
Annotation Cut-off: 50, Threshold: 0.95
Annotation Cut-off: 100, Threshold: 0.9
Annotation Cut-off: 100, Threshold: 0.95
Density
0
10
20
30
40
50
60
0.00
0.01
0.02
0.03
0.04
0.05
Fraction of all tissues that tested significant
