# Supplementary material for: Tissue enrichment analysis for C. elegans genomics
Source: BMC Bioinformatics. 2016 Sep 13;17(1):366. doi: 10.1186/s12859-016-1229-9 (PMC5020436; doi:10.1186/s12859-016-1229-9)
Supplement: Additional file 4 — Results. A folder containing a complete version of the results we generated for this paper. (ZIP 1597 kb) [file 12859_2016_1229_MOESM4_ESM.zip › output/SummaryInformation/fractissuesKDE_method=any.pdf]

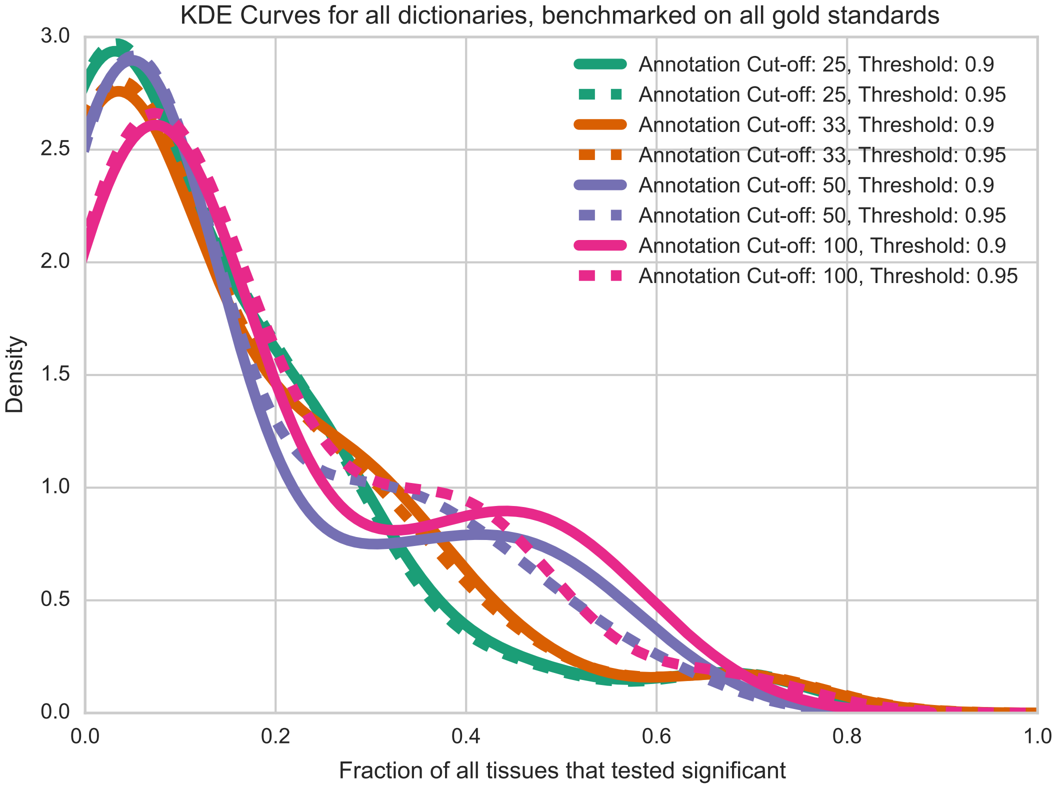

KDE Curves for all dictionaries, benchmarked on all gold standards
Density
Fraction of all tissues that tested significant
0.0
0.5
1.0
1.5
2.0
2.5
3.0
0.0
0.2
0.4
0.6
0.8
1.0
Annotation Cut-off: 25, Threshold: 0.9
Annotation Cut-off: 25, Threshold: 0.95
Annotation Cut-off: 33, Threshold: 0.9
Annotation Cut-off: 33, Threshold: 0.95
Annotation Cut-off: 50, Threshold: 0.9
Annotation Cut-off: 50, Threshold: 0.95
Annotation Cut-off: 100, Threshold: 0.9
Annotation Cut-off: 100, Threshold: 0.95
